# Investigation of Tongqiao Huashuan Granules’ effect on hippocampal neuron autophagy in vascular dementia rats via the PI3K/Akt-mTOR signaling pathway using network pharmacology and experimental validation

**DOI:** 10.3389/fneur.2025.1555411

**Published:** 2025-03-21

**Authors:** Xiaoqu Jiang, Shuyao Yu, Shuaifeng Yao, Sikai Wang, Jing Cai, Zhongsheng Tang, Shijie Zhu

**Affiliations:** ^1^First Clinical Medical College, Guizhou University of Traditional Chinese Medicine, Guiyang, China; ^2^Department of Neurology, First Affiliated Hospital of Guizhou University of Traditional Chinese Medicine, Guiyang, China; ^3^School of Basic Medical Sciences, Guizhou University of Traditional Chinese Medicine, Guiyang, China

**Keywords:** Tongqiao Huashuan Granules, vascular dementia, PI3K/AKT/mTOR, autophagy, mechanism of action

## Abstract

**Objective:**

This study aimed to apply network pharmacology to identify the active components and key targets of Tongqiao Huashuan Granules in vascular dementia (VaD) and to evaluate its effects on autophagy in hippocampal neurons of VaD rats through animal testing.

**Materials and methods:**

This study first employed network pharmacology (NP) to identify potential components and pathway targets for THg intervention in VaD. A modified two-vessel occlusion (2-VO) method was subsequently analyzed to establish a VaD rat model. Following the interventions, the spatial learning and memory abilities of the rats were assess using a water maze experiment. Morphological and structural changes in neuronal cells within the CA1 region of the rat hippocampus were examined using hematoxylin and eosin (HE) staining. Immunohistochemistry was utilized to assess the proportions of Beclin1-positive and LC3-positive cells in the CA1 region of each rat group, while performed Western blot analysis was conducted to measure protein expression levels of PI3K, p-PI3K, AKT, p-AKT, mTOR, p-mTOR, Beclin1, and LC3 in the hippocampal tissue of the rats.

**Results:**

A total of 76 active components were identified through network pharmacology analysis, with notable components including β-sitosterol, kaempferol, and cinnamophilin. In total, 825 key targets were identified, including IL1B, AKT1, JAK1, and MAPK3. THg and VaD shared 69 common genes. The Gene Ontology (GO) functional enrichment analysis yielded a total of 569 items (BP: 366, CC: 97, MF: 106). KEGG pathway enrichment analysis identified 143 signaling pathways, including TNF, MAPK, AGE-RAGE, and PI3K/Akt pathways. Subsequent validation experiments demonstrated that THg enhanced the learning and memory abilities of VaD rats, improve the morphology of neuronal cells in the CA1 region of the hippocampus, and decreasing the proportion of Beclin1-and LC3-positive cells in this region. Additionally, THg was shown to enhance the expression levels of p-PI3K, p-AKT, and p-mTOR proteins while reducing the expression levels of Beclin1 and LC3 proteins.

**Conclusion:**

This study represents the first investigation into the effects of THg intervention in VaD, indicating that its mechanism may involve inhibiting autophagy in hippocampal neurons through activation of the PI3K/Akt-mTOR signaling pathway.

## 1 Introduction

Vascular dementia (VaD) is a syndrome characterized by acquired cognitive dysfunction resulting from various cerebrovascular diseases and is regarded as the second most prevalent form of dementia in older adults, following Alzheimer’s disease (AD) (1). It represents an acquired cognitive impairment syndrome triggered by various cerebrovascular conditions. Epidemiological surveys indicate that VaD accounts for 15–20% of dementia cases in North America and Europe and approximately 30% in Asia and certain emerging nations. Similar to Alzheimer’s disease, the prevalence of VaD increases with age (2). The annual costs associated with the diagnosis and treatment of VaD exceed $200 billion (USD), placing a considerable financial burden on society and healthcare systems. Although VaD is the only form of dementia globally recognized as both preventable and treatable, no proven treatment is currently available. Therefore, it is essential to identify medications capable of targeting VaD at the molecular level.

The CA1 region of the hippocampus is one of the most widely studied areas of the cerebral cortex. It is composed of large pyramidal neurons arranged densely, making it easy to observe morphological changes in neuronal cells during experiments. As the core part of the hippocampal circuit, the CA1 region is particularly important for memory encoding, consolidation, and retrieval. It receives processed information from the hippocampal CA3 region and entorhinal cortex, and transmits it to other brain regions, such as the hypothalamus and cortex, thus participating in the formation and retrieval of long-term memories. During learning, synaptic plasticity in the CA1 region, particularly long-term potentiation (LTP), is considered one of the fundamental processes of learning and memory. This process is especially prominent in the CA1 region, directly affecting the storage and retrieval of information. Dysfunction in the CA1 region often leads to memory loss, especially in neurodegenerative diseases like Alzheimer’s disease.

The pathophysiological basis of VaD primarily involves chronic cerebral ischemia, which damages neurons by triggering cellular autophagy, inflammation, and apoptosis, ultimately leading to cognitive decline. Cellular autophagy is the process by which damaged cellular components, organelles, and proteins are encapsulated by autophagosomes, facilitating the removal of malfunctioning organelles and maintaining essential molecular pathways within cells (3). Recent studies indicate that the role of autophagy in VaD exhibits a “double-edged sword” effect. (1) VaD can be prevented by activating autophagy. During cerebral ischemia, a rapid increase in PINK1 in the outer mitochondrial membrane and the translocation of Parkin to the mitochondria trigger mitochondrial autophagy, which removes necrotic neurons (4). (2) On the other hand, autophagy may exacerbate VaD and represent a potential target for its treatment. Research indicates that WIN55,212-2, a cannabinoid receptor agonist, ameliorates learning and memory deficits in VaD mice by decreasing inflammatory markers and lowering Beclin-1 and LC3 protein levels (5). A class III phosphatidylinositol 3-kinase (PI3K) complex forms through the association of BECLIN1—an evolutionarily conserved positive regulator of autophagy—with other proteins, including Complex 1 (C1) and Complex 2 (C2). Within the PI3KIII complex, BECLIN1 functions as a scaffold, modulating the lipid kinase activity of the complex through conformational changes essential for autophagy and membrane transport. Besides maintaining autophagy levels in the body, BECLIN1 serves as a signaling hub, integrating signals from various cellular pathways (6, 7). Autophagosomes are marked by microtubule-associated protein 1 light chain 3 (LC3). The ubiquitin-proteasome system, catalyzed by Atg7 and Atg3, enables the conjugation of water-soluble LC3-I with phosphatidylethanolamine during autophagy. This process produces lipid-soluble LC3-II, a 14 kD protein that localizes to both the inner and outer membranes of autophagosomes. It is critical for elongating autophagic membranes and their fusion with other membrane structures (8).

Based on traditional Miao medicine and long-term clinical practice, the effective Tongqiao Huashuan Granules (THg) formula consists of eight herbs (the plant names have been checked with http://www.theplantlist.org, http://mpns.kew.org and www.dayi.org.cn), including *Sargentodoxa cuneata* (Oliv.) Rehd. & E. H. Wilson in C. S. Sargent (Chinese name: Daxueteng); *Mezoneuron cucullatum* (Roxb.) Wight & Arn (Chinese name: Jianxuefei); *Panax notoginseng* (Burkill) F.H.Chen (Chinese name: Sanqi); *Whitm.ania* Pigra Whitman (Chinese name: Shuizhi); *Gastrodia elata f. pallens* (Kitag.) Tuyama (Chinese name: Tianma); *Acorus calamus* var. *angustatus* Besser (Chinese name: Shichangpu); *Woodwardia japonica* (L. F.) Sm. (Chinese name: Gouji); *Astragalus membranaceus* Fisch. ex Bunge (Chinese name: Huangqi). The core therapeutic principles are to remove blood stasis, dispel wind, promote blood circulation, enhance blood flow, eliminate phlegm, and open the orifices. Previous studies have demonstrated that THg effectively reduces neurological function scores, alleviates disability, and enhances daily living abilities in the clinical treatment of ischemic stroke.

In preclinical experiments, researchers observed that THg significantly improved neurological deficits in a rat model of ischemia–reperfusion injury, increased VEGF expression in the frontal lobe and cerebellum of MCAO model rats, promote angiogenesis at the site of infarction, lower levels of ET and NO in the brain tissue of MCAO model rats, improves neurological deficits, reduce MDA levels, and increase SOD levels, thereby mitigating brain ischemic damage via antioxidative and anti-apoptotic mechanisms (9–11). The present study aimed to investigate whether THg could modulate hippocampal neuronal autophagy via the PI3K/Akt-mTOR signaling pathway, thereby ameliorating neural damage and cognitive impairment in VaD. These findings may offer novel theoretical insights for optimizing clinical prevention and treatment strategies for VaD.

## 2 Materials and methods

### 2.1 Network pharmacology analysis

#### 2.1.1 Screening of active ingredients and targets of THg

In this study, the Traditional Chinese Medicine Systems Pharmacology Database and Analysis Platform (TCMSP) was employed (TCMSP: http://tcmspw.com/tcmsp.php) to retrieve the active ingredients of four Chinese medicinal materials: “Daxueteng,” “Sanqi,” “Shichangpu,” and “Huangqi.” The selection of chemical components was based on the criteria of Drug-Likeness (DL) ≥ 0.18 and Oral Bioavailability (OB) ≥ 30 were selected. TCMSP serves as a central platform for the systemic pharmacology of traditional Chinese medicines, focusing on the in-depth exploration of complex relationships among Chinese medicines, their targets, and corresponding diseases through an consolidates pharmacological approach. The platform integrates extensive data resources, encompassing a wide range of chemical compositions (such as active compounds), detailed molecular target information, and interaction networks formed by these targets. Furthermore, TCMSP constructs association networks between drug targets and diseases (12).

For active ingredients absent in the TCMSP database, we utilized the High-Throughput Experimental Reference Database for Traditional Chinese Medicine (HERB: http://herb.ac.cn) and the Traditional Chinese Medicine Syndrome Association Database (SYMMAP: http://www.symmap.org) to identify active ingredients in “Shuizhi,” “Tianma,” “Gouji,” and “Jianxuefei. HERB is a high-throughput experimental and reference-guided TCM database that establishes data-driven linkages between traditional Chinese medicine (TCM) and modern medicine (MM), offering robust support for further TCM pharmacological research (13). The SymMap database functions as an innovative cross-referencing tool that effectively integrates the foundational philosophy of Traditional Chinese Medicine (TCM) with the empirical science of Modern Medicine (MM). The database encompasses a wide range of herbal resources, active ingredients, action targets, detailed descriptions of clinical symptoms, and comprehensive information on related diseases, making it an extensive and invaluable resource for researchers (14).

To integrate active ingredients identified from different databases, we used the Organic Small Molecule Bioactivity Database, PubChem^1^ (15), for comparative validation and saved the corresponding SDF structures. The SDF structures of the identified components were imported into the SwissTargetPrediction database^2^ (16), with the species restricted to “*Homo sapiens*,” to screen for final drug targets with a probability ≥0.

#### 2.1.2 Screening of VaD-related genes

The GeneCards database^3^ (17), the OMIM database^4^ (18), and the CTD database^5^ (19) were queried using the keyword “Vascular dementia” to identify targets with reported scores ≥20 related to VaD. After integrating the identified disease targets, the therapeutic targets for preventing and treating VaD were determined.

#### 2.1.3 Compound-target network

The Venny 2.1.0 analysis tool^6^ was used to map the component targets of THg Granules to VaD-related targets, extracting intersecting targets that represent the potential therapeutic targets of THg for treating vascular dementia (VaD). A Venn diagram was generated for visualization.

#### 2.1.4 Construction of compound-target network for THg formula

Cytoscape, a bioinformatics program, integrates various molecular state data, including genotypes, gene expression, and biological networks, into a graphical interface. Microsoft Excel 2016 was used to create network text and attribute files for the active ingredient-common target network in THg for this study. The pharmacological network diagram of THg was subsequently generated by importing these files into Cytoscape 3.7.2 (20). In the network, “edges” represent the interactions between nodes, illustrating the intricate connections between chemical constituents and potential targets, while “nodes” denote the elements and action targets of the eight Chinese medicinal herbs. To identify the essential elements of THg for treating VaD, the degree value of each node was calculated using the software’s Network Analyzer function.

#### 2.1.5 Construction of PPI network

To analyze cross-targeted genes for protein–protein interactions relevant to THg’s treatment of vascular dementia (VaD), the genes were submitted to the STRING database.^7^ To analyze cross-targeted genes for protein–protein interactions relevant to THg’s treatment of vascular dementia (VaD), the genes were submitted to the STRING database (see footnote 7) (21). The species restriction was set to “*Homo sapiens*,” the data analysis method was configured for “Multiple proteins,” and a confidence level of ≥0.9 was selected. The protein interaction data were further analyzed using Cytoscape 3.7.2 to identify the primary targets of THg for treating VaD, while the Network Analyzer module was employed to calculate the degree values of core genes and examine the network architecture.

#### 2.1.6 GO function and KEGG pathway enrichment analysis

The DAVID database^8^ was utilized to perform Gene Ontology (GO) functional and KEGG pathway analyses on the overlapping genes targeted by THg for the treatment of VaD. Biological processes (BP), cellular components (CC), and molecular functions (MF) were interpreted to clarify the potential targets of THg (22). Microbiome Informatics,^9^ an online graphing application, was employed to visualize the data.

### 2.2 Verification of animal experiments

#### 2.2.1 Experimental animals

Healthy male Sprague–Dawley (SD) rats, weighing 250–300 g and aged 6–8 weeks, were provided by Beijing HFK Bioscience Co., Ltd. [SCXK(JING)2019-0008]. The rats were housed in a temperature-controlled environment maintained at 22 ± 0.2°C and 22 ± 2% relative humidity. They were acclimatized for at least 1 week and were provided with free access to food and water prior to the start of the experiment.

#### 2.2.2 Ethical approval

This study adhered strictly to the Chinese Code of Practice for the Management and Technical Conduct of Animal Experiments. The design and methodology of this animal experiment were approved by the Animal Experiment Ethics Review Committee of Guizhou University of Traditional Chinese Medicine. (Animal ethics review approval number: 20241022002). All surgery was performed under sodium pentobarbital anesthesia, and all efforts were made to minimize suffering.

#### 2.2.3 Experimental intervention drugs

The batch number of THg used in this study was 20,221,201, and it was prepared by the First Affiliated Hospital of Guizhou University of Traditional Chinese Medicine (Guiyang, Guizhou, China). The daily dosage of THg for adults is 30 g, which is equivalent to the following herbs: Daxueteng 30 g, Jianxuefei 15 g, Sanqi 10 g, Shuizhi 10 g, Tianma 15 g, Shichangpu 12 g, Gouji 15 g, and Huangqi 15 g. In the present experiment, THg was dissolved in saline. Following the ‘Methodology of Pharmacological Experiments,’ the daily dosage was adjusted based on the body surface area of the rats, resulting in low (1.875 g/kg/day), medium (3.5 g/kg/day), and high (7 g/kg/day) doses. Donepezil Hydrochloride (trade name: Sibohai; approval number: National Drug Approval H20010723) was supplied by Chongqing Zhisi Pharmaceutical Co., Ltd.

#### 2.2.4 Main antibodies and reagents

Hematoxylin–Eosin staining solution was acquired from Wuhan Seville Biotechnology Co., Ltd. (Wuhan, China). Antibodies for PI3K (Catalog Number: AF6241, RRID: AB_2835340), AKT (Catalog Number: AF0836, RRID: AB_2834120), mTOR (Catalog Number: AF6241, RRID: AB_2835340), Beclin1 (Catalog Number: AF5128, RRID: AB_2837614), and LC3 (Catalog Number: AF5402, RRID: AB_2837886) were obtained from Affinity Biosciences (Jiangsu, China). The 12.5% PAGE Gel Rapid Preparation Kit was obtained from Yaen Biomedical Technology Co., Ltd. (Shanghai, China). The BCA Protein Concentration Determination Kit was obtained from Solarbio Technology Co., Ltd. (Beijing, China). Antibodies for p-PI3K (Catalog Number: AF3241, RRID: AB_2834667) were sourced from Affinity Biosciences (Jiangsu, China), while p-AKT (Catalog Number: 4060, RRID: AB_2936343) and p-mTOR (Catalog Number: 5536, RRID: AB_2861149) antibodies were acquired from Cell Signaling Technology (Massachusetts, USA).

#### 2.2.5 Animal grouping

A random number table was used to allocate the rats into six groups before the experiment: Sham group, Model group (VaD), Donepezil group, Low-dose THg group (L-THg), Medium-dose THg group (M-THg), and High-dose THg group (H-THg). Each group contained 12 rats.

#### 2.2.6 Animal model preparation and intervention

Prior to treatment, the animals underwent a 12-h fast with unrestricted access to water. Anesthesia was induced using intraperitoneal injections of 1.5% pentobarbital sodium (45 mg/kg). The rats were positioned in a supine posture and secured to the operating table with rubber bands around the head, limbs, and body. After trimming the hair around the neck, the area was sterilized with iodine. A 1 cm longitudinal incision was made approximately 0.5 cm lateral to the neck’s midline using a surgical blade. The carotid artery was carefully exposed between the sternohyoid and sternocleidomastoid muscles using curved forceps to gradually separate the subcutaneous tissue. After separating the carotid artery and vagus nerve, the artery was double-ligated with a 4–0 silk suture. The vessels and surrounding tissues were repositioned to their original anatomical locations, and the skin was intermittently sutured. The incision site was disinfected again. Postoperatively, iodine was used daily for 3 days to disinfect the wound and surrounding skin. On the fifth postoperative day, the opposite side of the neck’s midline underwent a similar procedure. Postoperative care followed the same procedures described above.

The success of the model was evaluated using the Zea-Longa scoring system after surgery completion and the rats’ recovery from anesthesia (23). Rats with scores of 0 or 4 were excluded as unsatisfactory models. The remaining rats were divided into groups for pharmacological intervention. The Donepezil group received 1 mg/kg/day by gavage, the L-THg solution group received 1.875 g/kg/day by gavage, the M-THg solution group received 3.75 g/kg/day by gavage, the H-THg solution group received 7.5 g/kg/day by gavage, and the VaD group and the Sham group were given an equivalent volume of regular saline. Gavage treatment was administered for 21 days.

#### 2.2.7 Morris water maze (MWM)

The rats underwent a 6-day Morris water maze trial after a 21-day treatment period to assess their spatial learning and memory abilities. To reduce stress during the experiment, the rats were allowed a two-minute acclimation period in the pool before the trial commenced. For each group of rats, escape latency, platform crossings, and the time spent in the target quadrant were measured. During the first 5 days of the experiment, the platform remained in the same fixed quadrant, and the rats were gently placed into the water from one of four starting locations (quadrants I–IV) facing the pool wall. If a rat took longer than 120 s to reach the platform, its escape latency was recorded as 120 s after being guided to the platform for a 10-s adaptation period. On the sixth day, the platform was removed for the second phase, and the rats were released into the water from a quadrant opposite the platform’s prior location. The rats were timed and the number of crossings over the previous platform location within 120 s, as well as the time spent in the target quadrant, were recorded (24).

#### 2.2.8 Hematoxylin and eosin staining (HE)

After the Morris water maze experiment, 1.5% pentobarbital sodium (45 mg/kg) was administered intraperitoneally to rats as an anesthetic. Following anesthesia, the rats were dissected to expose their hearts. Vascular clamping was performed, the right atrium was incised, and the blood vessels were flushed with 150 mL of saline solution, followed by perfusion fixation with 250 mL of 4% paraformaldehyde. The perfusion was carried out slowly over a total duration of 90 min. The brain was then removed and fixed in 4% paraformaldehyde solution for further hematoxylin and eosin (HE) staining and immunohistochemistry. The brain was embedded in paraffin wax, and 4 μm sections were cut. The sections were deparaffinized with xylene, dehydrated through a graded ethanol-to-water series, stained with hematoxylin and eosin, and mounted. Under a microscope, the morphology of the hippocampal CA1 region was examined in each group of rats (25).

#### 2.2.9 Immunohistochemistry

The paraffin sections were incubated overnight at 37°C and then baked at 60°C for 2 h before deparaffinization. The portions were sequentially placed in xylene I and II for 10 min each. After ethanol gradient and antigen retrieval, endogenous peroxidase was blocked with 3% H2O2, followed by incubation with sheep serum at room temperature for 30 min. Primary antibodies (BECLIN1, diluted 1:50; LC3, diluted 1:50 to 1:200) were added and incubated overnight at 4°C. The next day, after rewarming, the primary antibodies were washed off, and a secondary antibody conjugated with HRP was added and incubated for 60 min at 37°C, was added. After rinsing, freshly prepared DAB colour development solution was added dropwise, the sections were re-stained with hematoxylin, and observed under the microscope to control the time of colour development under the dehydrated and transparent sealing film. Afterwards, it was examined and photographed under the microscope (Dorsal CA1 region), and The ImageJ software was used to analyze and measure the average optical density of BECLIN1 and LC3-positive granules in the hippocampal CA1 region under each high-power field. In ImageJ software, import the digitized images of stained tissue sections and convert them into 8-bit grayscale images. Use the “Straight Line” tool to calibrate the spatial resolution (μm/pixel). Employ the “Freehand selections” tool to precisely outline the regions of positive expression. Subsequently, configure the measurement parameters: go to “Analyze > Set Measurements,” check “Mean gray value” and “Integrated density,” and set the measurement unit to “Gray Value.” Click “Analyze > Measure” to obtain the optical density values of the target regions (26).

#### 2.2.10 Western blot analysis (WB)

A suitable amount of rat hippocampal tissue (80 mg) was homogenized in RIPA lysis buffer using ultrasonic vibration, and centrifuged at 4°C and 12,000 rpm for 5 min. The supernatant was collected, and the protein concentration was determined using the BCA assay. After sampling and electrophoresis, the membrane was transferred and blocked with 5% nonfat milk. Primary antibodies targeting PI3K, p-PI3K, AKT, p-AKT, mTOR, p-mTOR, Beclin1, and LC3 were added at dilutions ranging from 1:500 to 1:2,000, and the membrane was gently agitated at 4°C overnight. After a final wash, the corresponding HRP-conjugated secondary antibody (1:10,000) was added and incubated at 37°C. After washing, enhanced chemiluminescence (ECL) detection was performed, and the target bands were analyzed using ImageJ (27).

#### 2.2.11 Statistical analysis

All experimental data were analyzed statistically using SPSS 26.0. For normally distributed quantitative data, the mean ± standard deviation (x̄ ± s) was used for representation. A one-way analysis of variance (ANOVA) was performed to compare the means of multiple samples. Post-hoc pairwise comparisons were carried out using the LSD test under conditions of homogeneity of variances. Statistical significance was defined as *p* < 0.05.

## 3 Results

### 3.1 Analysis based on network pharmacology

#### 3.1.1 THg active ingredient and target screening results

The TCMSP, SYMMAP, and HERB databases were utilized to identify the active components of each herb in THg. Redundant genes were excluded based on the established screening criteria. The Swiss Target Prediction and PubChem platforms were used to collect target data for the active components. After excluding six components without matching target information, 75 active components were retained in THg. Of these, the active components are as follows: three in Daxueteng, twenty-three in Jianxuefei, eight in Sanqi, five in Shuizhi, five in Gouji, ten in Tianma, four in Shichangpu, and seventeen in Huangqi (Supplementary Table 1). Following the standardization of target data from the PubChem and Swiss Target Prediction databases, 825 targets connected to the active components in THg were gathered after invalid and duplicate targets were eliminated (Supplementary Table 2).

#### 3.1.2 Intersection of compound and disease genes

**Figure 1:**
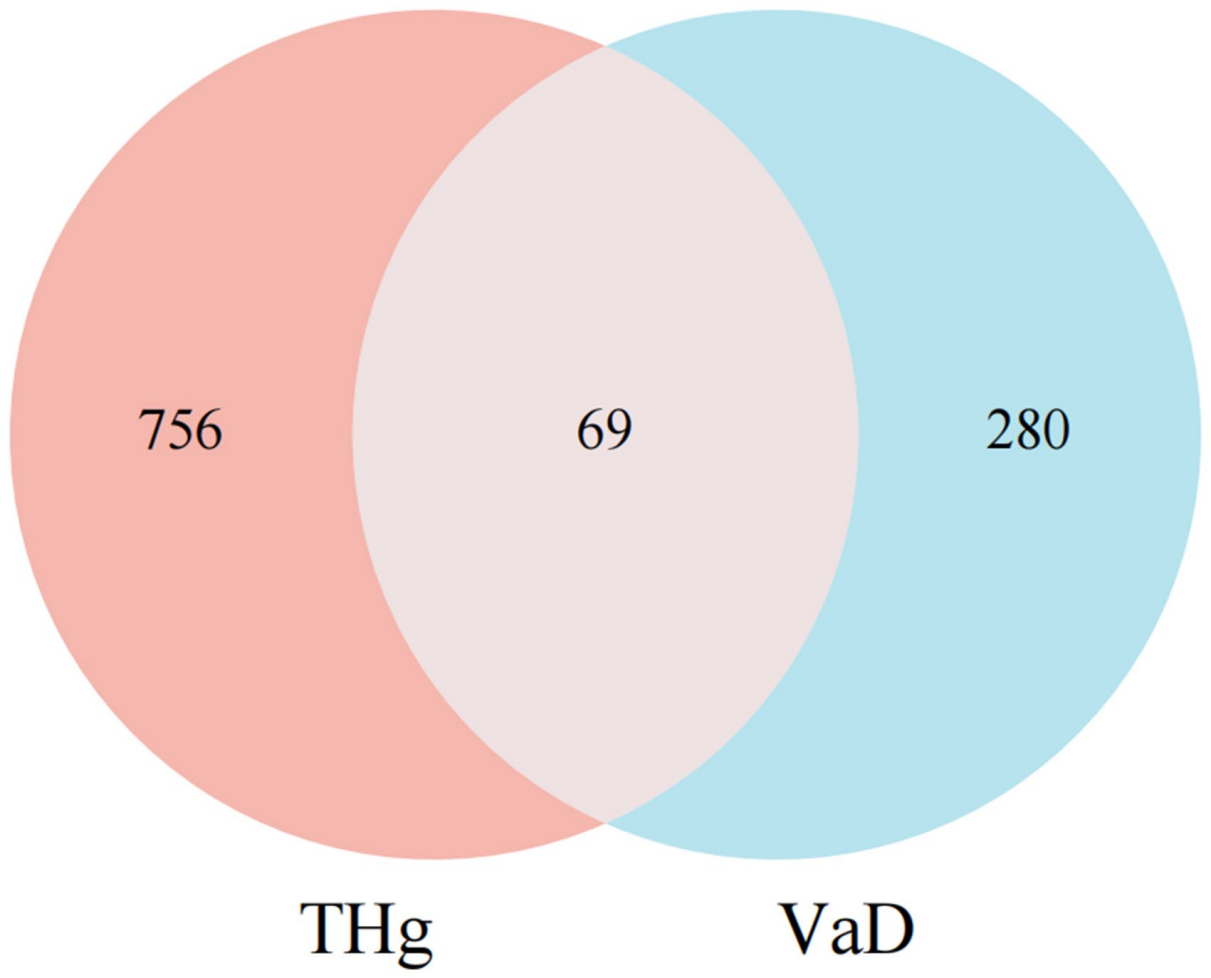
Intersection of Compound and Disease Genes. Purple circles indicate the targets of Tongqiao Huashuan Granules, and blue circles indicate the targets of Vascular Dementia Disease.

The keyword “Vascular dementia” was entered into the GeneCards, OMIM, and CTD databases to analyze gene targets associated with the disease while removing duplicate targets. A total of 349 disease targets associated with VaD were identified (Supplementary Table 3). The intersection of 825 predicted compound targets and 349 VaD-related genes identified 69 targets for THg in the treatment of VaD (Figure 1).

#### 3.1.3 Compound and network target construction results

**Figure 2:**
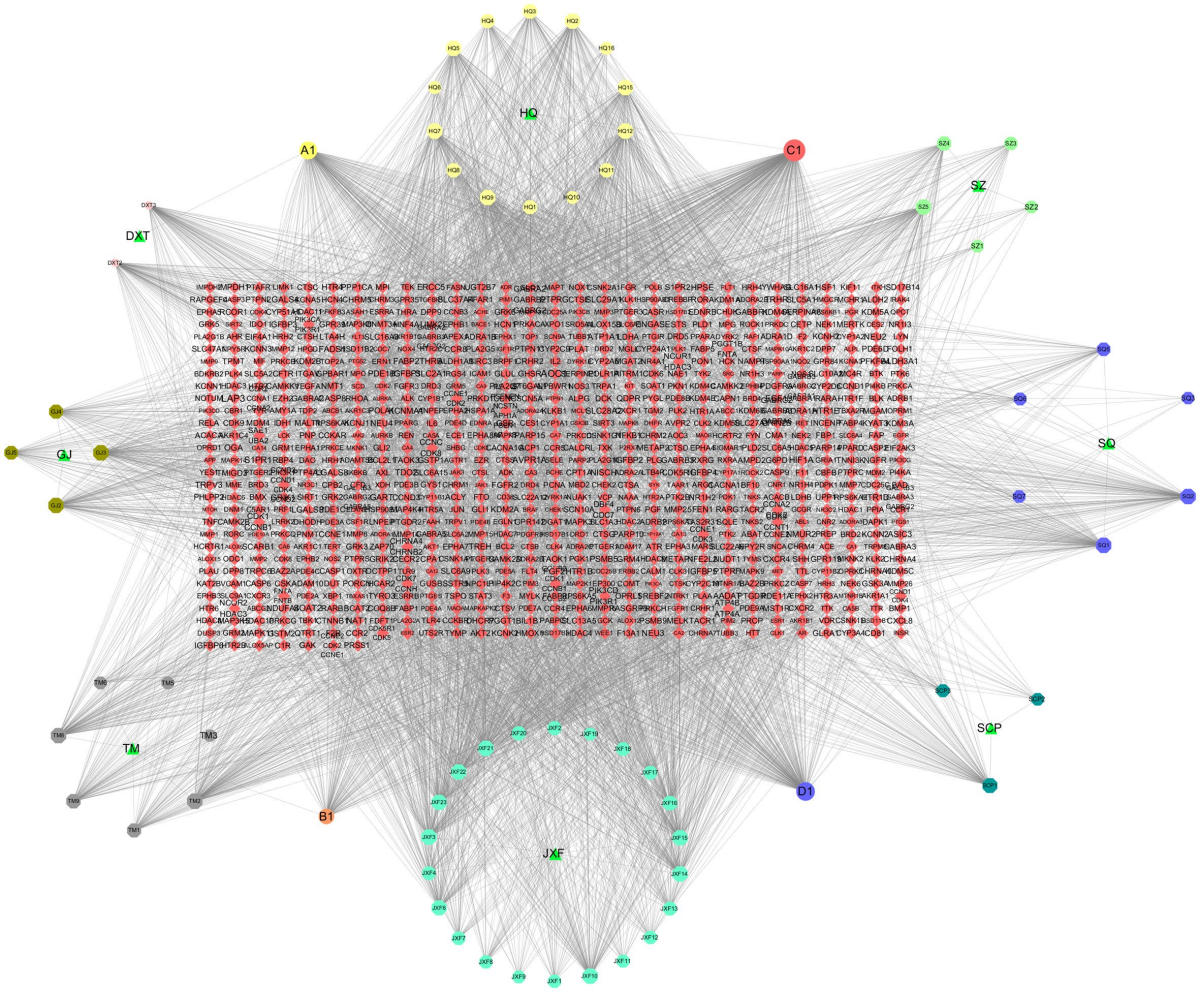
Compound and Network Target Construction Results. Included 914 nodes (8 drug nodes, 76 compound nodes, 830 target nodes) and 4,564 edges.

Cytoscape 3.7.2 was employed to construct the compound-target network, comprising 914 nodes (8 drug nodes, 76 compound nodes, and 830 target nodes) and 4,564 edges. CytoHubba technology was used to identify key active substances in the network: 1. beta-sitosterol (MOL000358): Daxueteng, Sanqi, Jianxuefei, Tianma; 2. Calycosin (MOL000417): Huangqi, Tianma; 3. Kaempferol (MOL000422): Huangqi, Shichangpu, Gouji; 4. Quercetin (MOL000098): Huangqi, Sanqi (Figure 2).

#### 3.1.4 Results of PPI network construction

**Figure 3:**
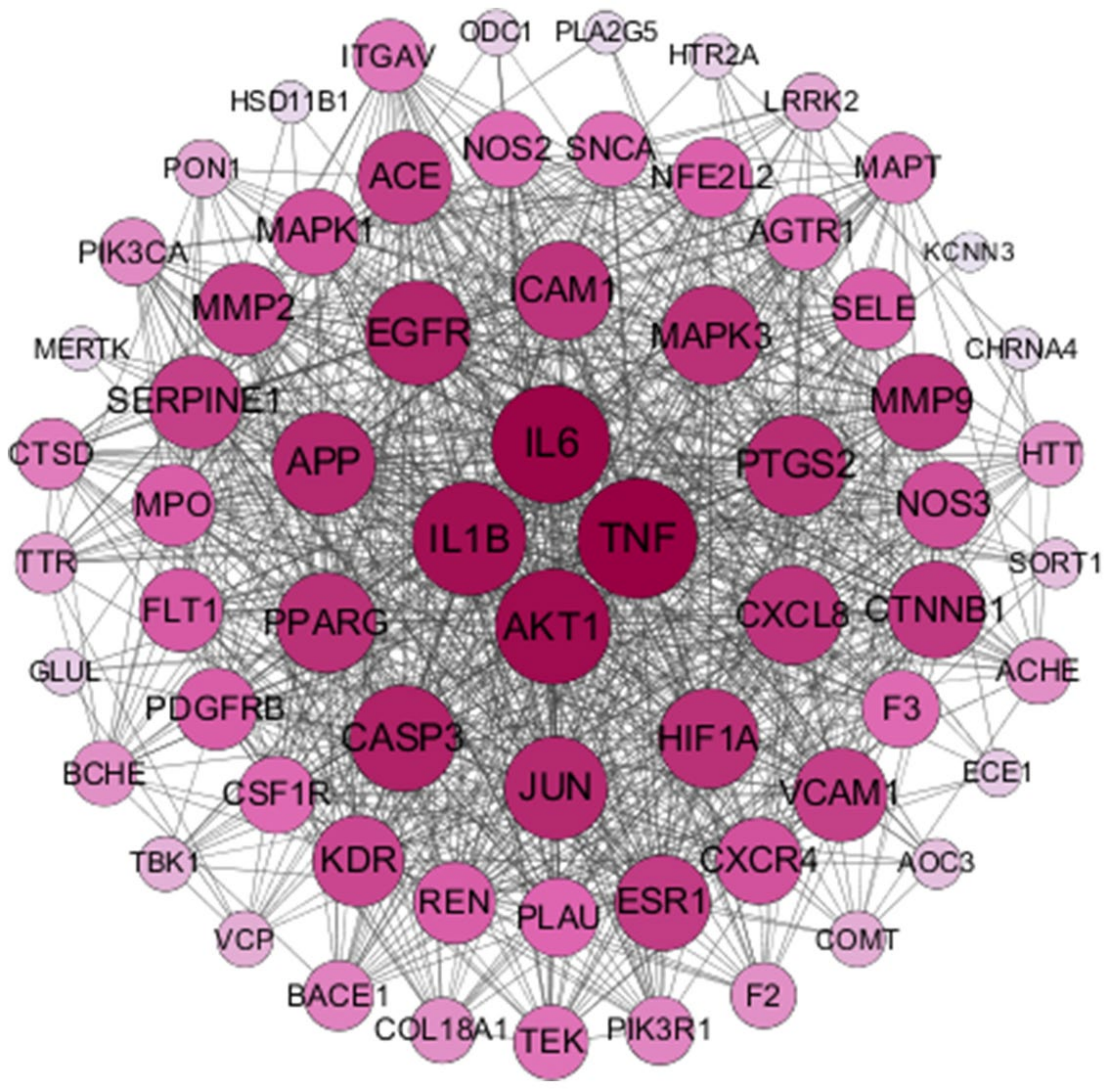
Results of PPI Network Construction. The obtained‘compound-disease-target’was analysed by the String database for PPI network construction, resulting in a total of 66 nodes and 861 edges.

To further investigate how THg interacts with disease target proteins, we constructed a protein–protein interaction (PPI) network using the “compound-disease-target” data from the STRING database, restricting the species to “*Homo sapiens*” and setting a confidence threshold of ≥0.9. A gene PPI network for THg-treated VaD was generated using Cytoscape 3.7.2, comprising 66 nodes and 861 edges (Figure 3). Core gene analysis was conducted using the cytoNCA plugin, identifying the top 10 genes ranked by degree: TNF, IL6, AKT1, IL1B, CASP3, EGFR, APP, JUN, PIGS2, and MAPK3, which may act as core targets of THg in treating VaD.

#### 3.1.5 Results of the KEGG pathway enrichment analysis and the GO function

**Figure 4:**
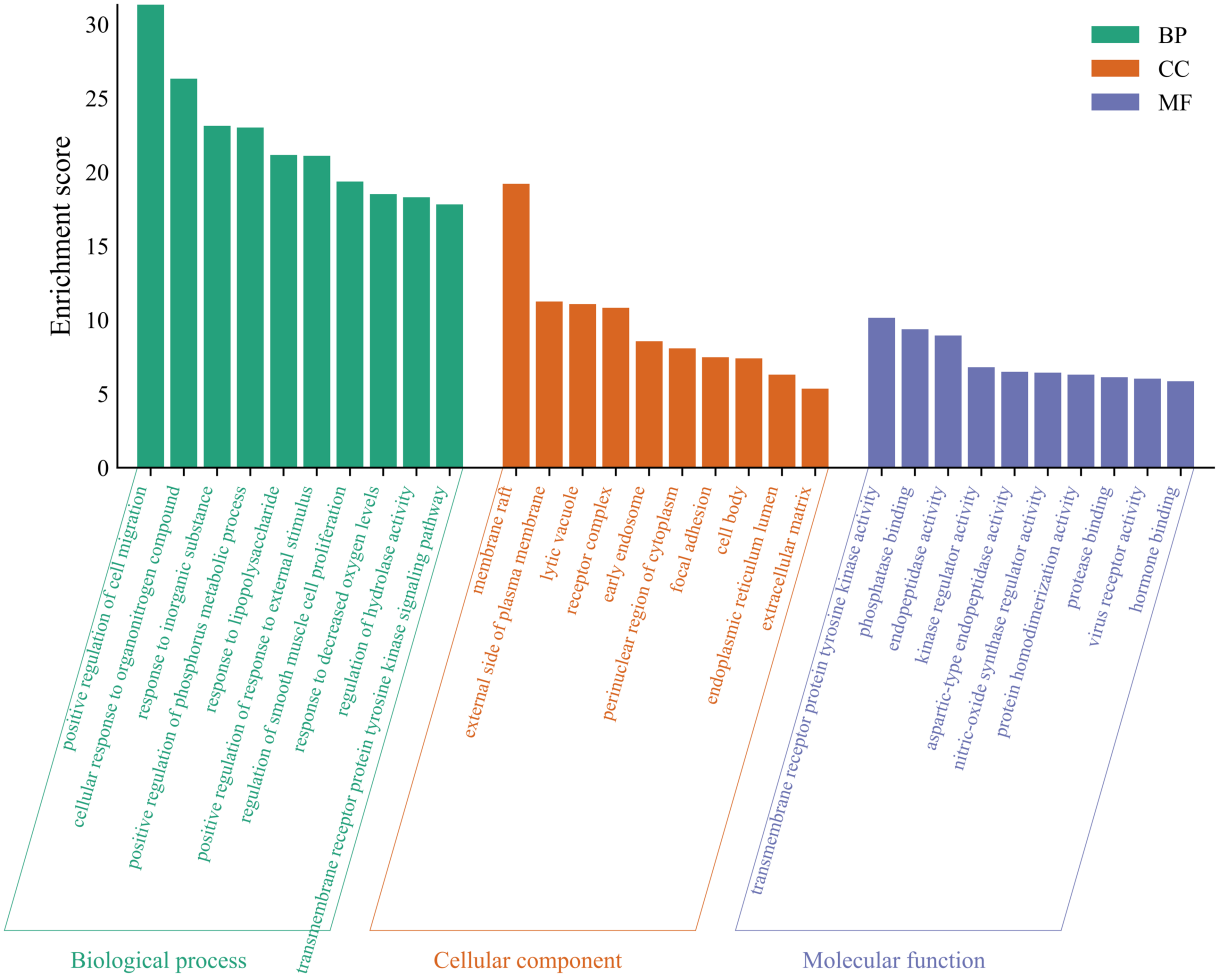
A GO analysis of 69 protein targets for THg treatment of VD. Items obtained from the GO analysis were classified and annotated according to biological process (BP), cellular component (CC) and molecular function (MF). The top 10 biological processes closely related to BP, CC, and MF were selected based on *p*-values.

**Figure 5:**
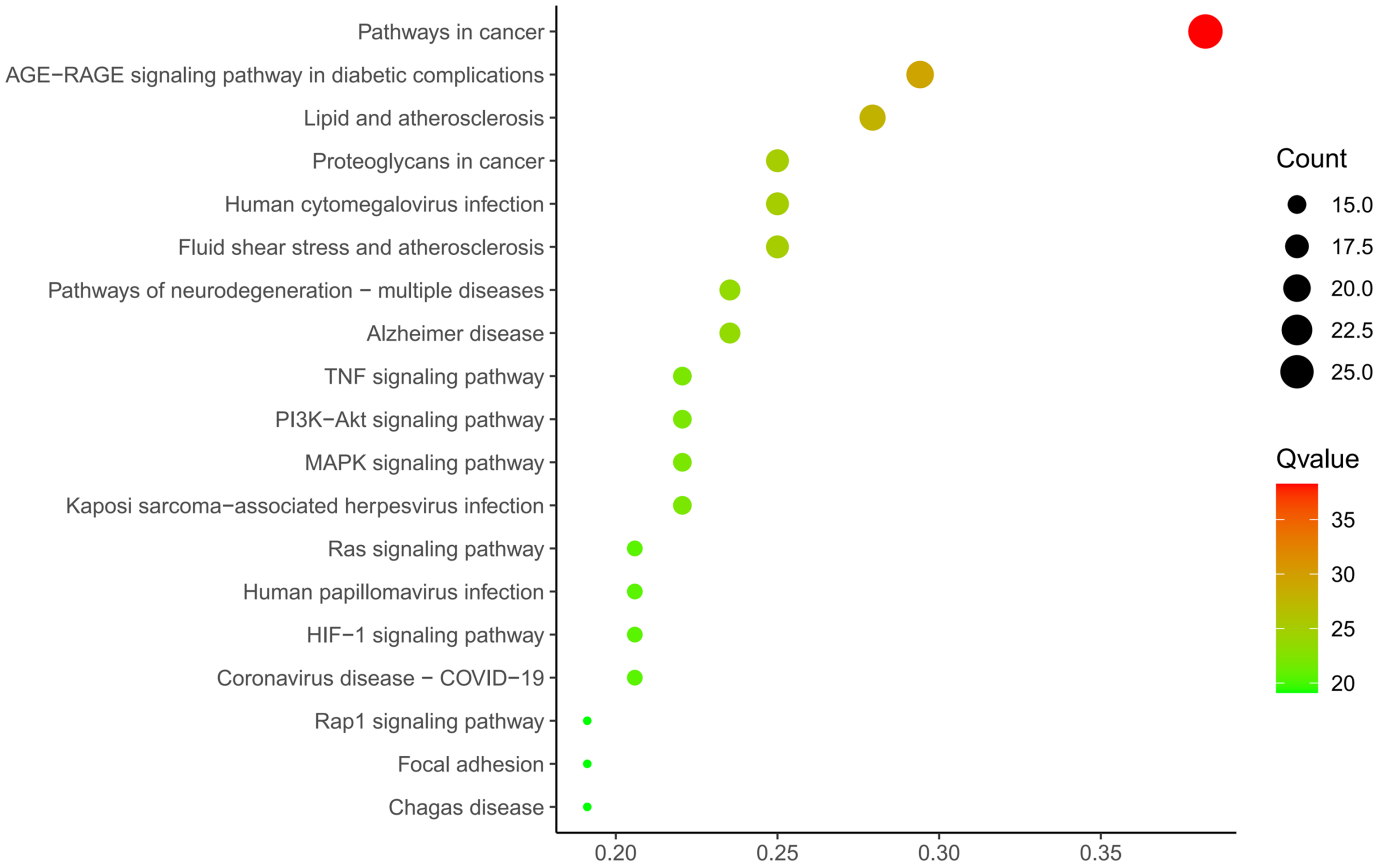
KEGG analysis of 69 protein targets for THg treatment of VD. KEGG functional enrichment analysis showed that a total of 143 relevant pathways were screened, and we selected the PI3K/AKT pathway from the first 20 pathways for study.

A total of 69 gene targets related to THg-treated VaD were analyzed using the DAVID database for Gene Ontology (GO) functional and KEGG pathway enrichment analyses. The Gene Ontology annotation library was used to classify and annotate the items obtained from the GO analysis into Biological Processes (BP), Cellular Components (CC), and Molecular Functions (MF). The top 10 biological processes related to BP, CC, and MF were selected based on *p*-values, including positive regulation of cell migration, membrane rafts, and tyrosine kinase activity of transmembrane receptor proteins (Figure 4). KEGG pathway enrichment analysis identified 143 relevant pathways, with the top 20 including the AGE-RAGE signaling pathway, TNF signaling pathway, PI3K-Akt signaling pathway, and MAPK signaling pathway (Figure 5).

### 3.2 Animal experiment results analysis

#### 3.2.1 THg enhance memory and spatial learning capabilities in VaD rats

**Figure 6:**
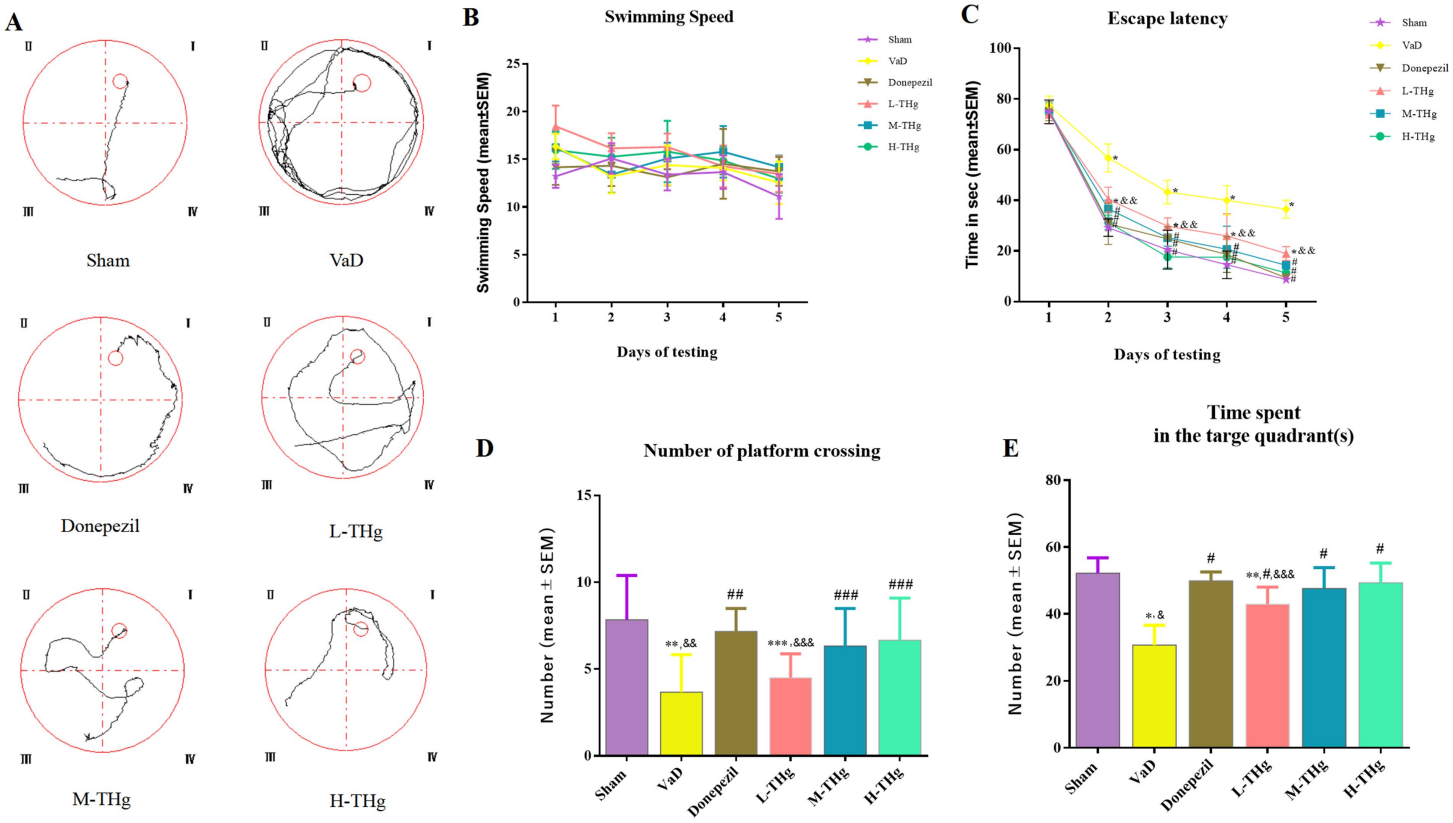
Morris water maze test results of rats in each group. **(A)** Swimming trajectories of representative samples from different groups of rats; **(B)** Swimming speed of different groups of rats; **(C)** Escape latency period from day 1 to day 5 during the training phase; **(D)** Number of plateau crossings during the testing phase in different groups of rats; **(E)** Spent time in the target quadrant during the testing phase in different groups of rats. Data were expressed as mean ± SEM. Compared with Sham group: *p < 0.001, ** *p* < 0.01, ***p < 0.05; Compared with VD group: ^#^*p* < 0.001, ^##^*p* < 0.01, ^###^*p* < 0.05; Compared with Donepezil group: ^&^*p* < 0.001, ^&&^*p* < 0.01, ^&&&^*p* < 0.05.

Spatial learning and memory were assessed in the rats using the Morris water maze test after a 21-day pharmacological intervention (Figure 6A). The groups showed no significant differences in average swimming speeds (Figure 6B). Rats in each group experienced varying degrees of escape latency shortening over the first 5 days of the water maze spatial navigation test as the number of training days increased. The VaD group rats exhibited a significantly longer escape latency compared to those in the Sham group. The escape latency of the rats in the other pharmacological intervention groups improved significantly compared to the VaD group (*p* < 0.05). Notably, the groups receiving high doses of THg and donepezil hydrochloride showed the greatest decreases in escape latency (Figure 6C). The VaD group rats demonstrated a statistically significant reduction in both the duration of stay in the target quadrant and the number of platform crossings on the last day of the water maze spatial exploration compared to the Sham group (p < 0.05). Notably, the groups receiving high-dose THg and donepezil hydrochloride had the greatest increase in the number of platform crossings and the duration of time spent in the target quadrant (Figs. 6D-E). These findings suggest that THg treatment can effectively reverse memory and spatial learning impairments in VaD rats.

#### 3.2.2 THg improve neuronal cell morphology in the hippocampal CA1 region of VaD rats

**Figure 7:**
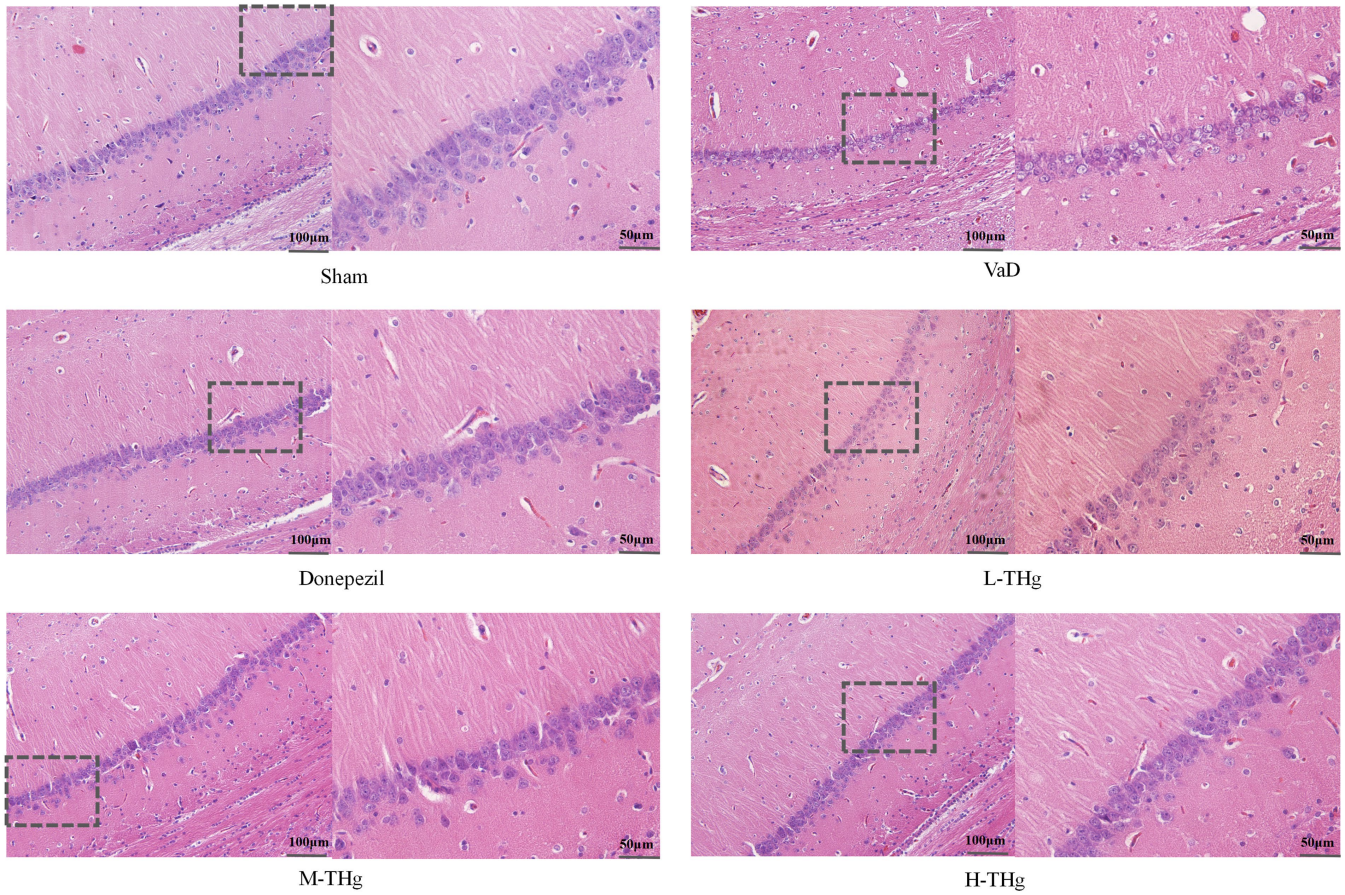
HE staining results of rats in each group. Rat brain tissue hippocampal CA1 area was stained with hematoxylin and eosin (H&E) and photographed under a microscope at 200× magnification. Scale bars represent 100 μm. typical areas were magnified at 400×. Scale bar represents 50 μm.

Following behavioral testing, histological evaluation of neuronal cells in the hippocampal CA1 region was performed on rats in each group using HE staining. The results showed that the CA1 region of the hippocampus in Sham group rats displayed a well-organized and intact neuronal cell structure, characterized by large, spherical nuclei centrally located in the cytoplasm, complete cytoplasmic structure, and distinct cell body outlines. Rats in the VaD group showed looser hippocampal tissue compared to the Sham group. In most neurons, the cytoplasm was contracted and distorted, making the neurons appear wrinkled and disorganized. The nuclei had shrunk into irregular polygons, with visible intercellular spaces, reduced cytoplasmic content, and severely damaged cells observed. Compared to the VaD group, hippocampal tissue damage was significantly alleviated following treatment with donepezil hydrochloride and different doses of THg, leading to substantial morphological restoration (Figure 7).

#### 3.2.3 THg can reduce the optical density ratio of autophagic protein-positive granules in the hippocampal CA1 region of VaD rats

**Figure 8:**
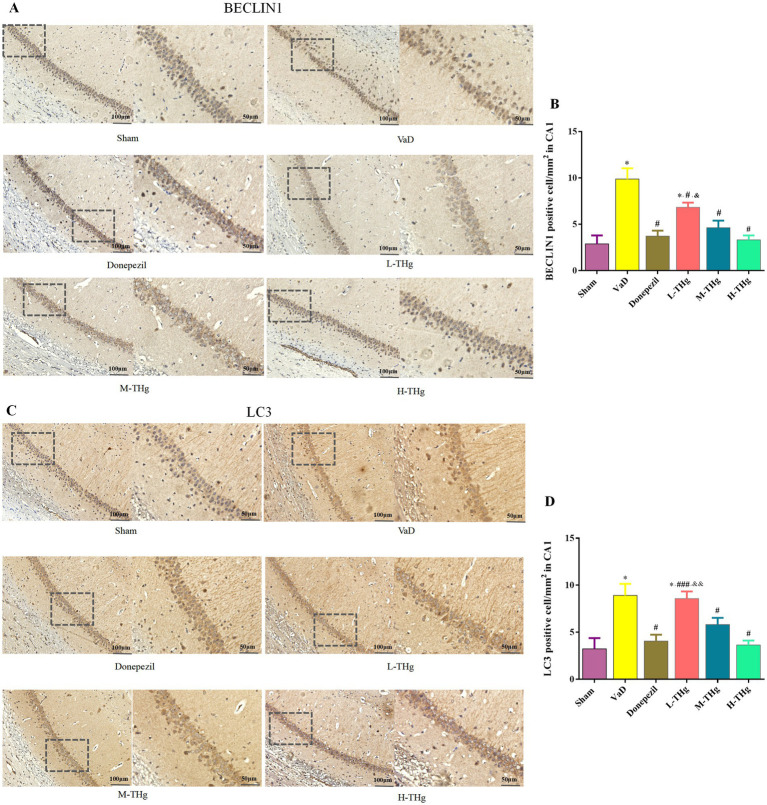
Immunohistochemistry results of rats in each group. Immunohistochemistry of autophagy proteins in the hippocampal CA1 region of rat brain tissues of each group: **(A,B)** BECLIN1, **(C,D)** LC3 were observed at the protein level and photographed under a microscope at 200× magnification. Scale bars represent 100 μm. typical areas were magnified at 400×. Scale bar represents 50 μm. Compared with Sham group: *p < 0.001, **p < 0.01, ***p < 0.05; Compared with VD group: ^#^p < 0.001, ^##^*p* < 0.01, ^###^*p* < 0.05; Compared with Donepezil group: ^&^*p* < 0.001, ^&&^*p* < 0.01, ^&&&^*p* < 0.05.

The optical density ratios of BECLIN1 and LC3 autophagic proteins in the hippocampal CA1 region of rats in each group were measured using immunohistochemistry. Rats in all groups exhibited the expression of BECLIN1 and LC3, primarily localized to the cytoplasm of neural cells. Positive cells exhibited cytoplasmic staining in light yellow or brown. The results showed that rats in the VaD group had significantly higher average optical density values (*p* < 0.05) and more intense cytoplasmic staining in the hippocampal CA1 region compared to the Sham group. Following pharmacological intervention, rats treated with donepezil hydrochloride and different doses of THg showed a marked reduction in autophagic protein expression in the hippocampal CA1 region. Notably, the H-THg group showed the most significant reduction in autophagic protein expression (*p* < 0.05) among the traditional Chinese medicine treatment groups. (Figures 8A-D).

#### 3.2.4 THg can activate the PI3K/Akt-mTOR signaling pathway

**Figure 9:**
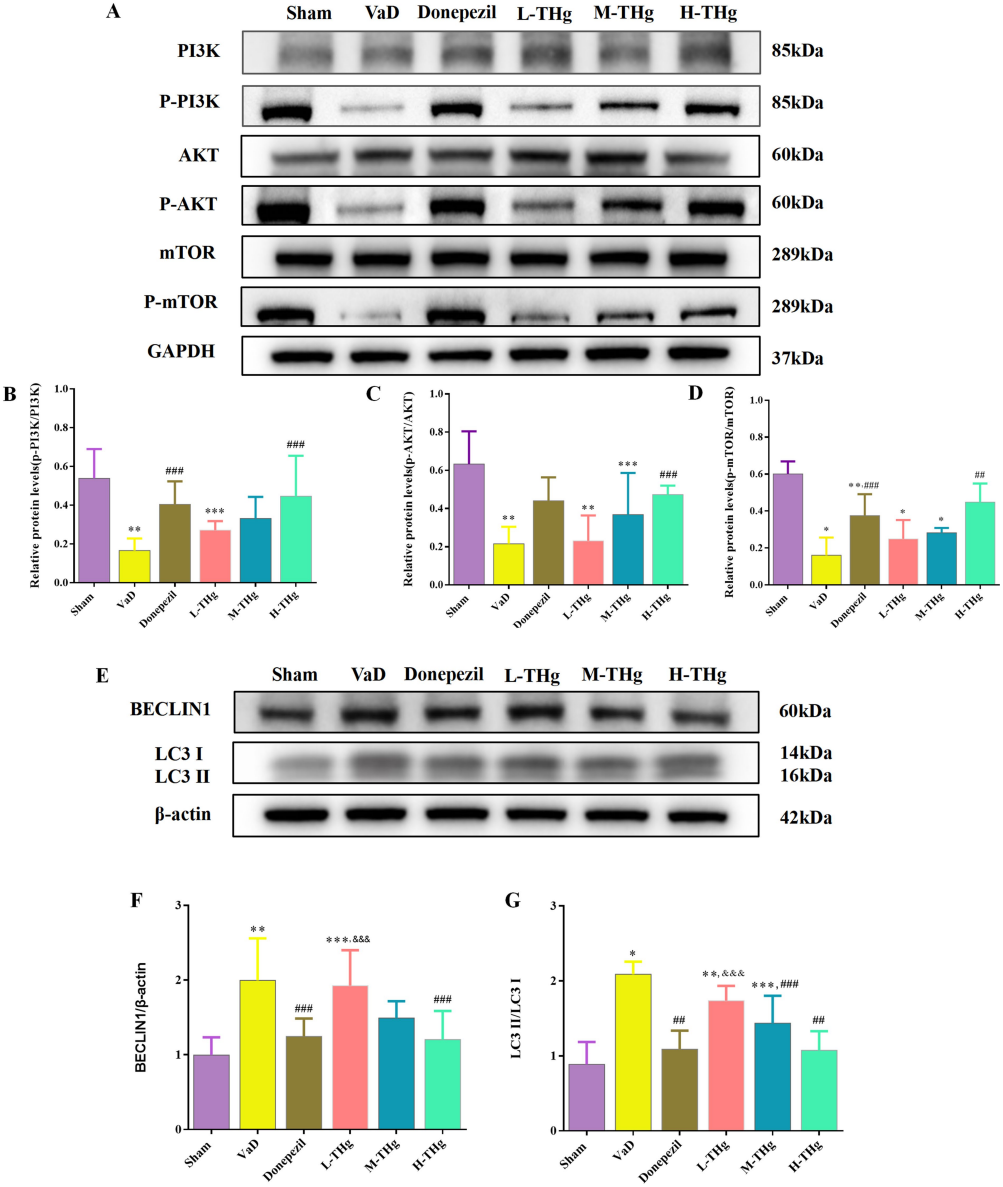
Effect of THg on protein expression of PI3K/AKT/mTOR signaling pathway. **(A)** Representative western blot of PI3K/AKT/mTOR signaling pathway related proteins. **(B–D)** Measurement of the optical intensity of p-PI3K, p-AKT, p-mTOR. **(E)** Representative western blot of classical autophagy proteins: BECLIN1, LC3. **(F,G)** Measurement of the optical intensity of BECLIN1, LC3. Compared with Sham group: *p < 0.001, **p < 0.01, ***p < 0.05; Compared with VD group: ^#^*p* < 0.001, ^##^*p* < 0.01, ^###^*p* < 0.05; Compared with Donepezil group: ^&^*p* < 0.001, ^&&^*p* < 0.01, ^&&&^*p* < 0.05.

To further investigate the mechanism by which the Tongtiao and Thrombolysis granules regulate autophagy levels in the hippocampal neuronal cells of VaD rats, we assessed the expression of proteins associated with the PI3K/Akt-mTOR signaling pathway using Western blot analysis (Figures 9A-D). The results showed that autophagic protein expression levels were elevated in the hippocampus of VaD group rats compared to the Sham group, accompanied by a significant decrease in p-PI3K, p-Akt, and p-mTOR protein expression and a marked increase in BECLIN1 and LC3 protein expression. In the medication groups after intervention with donepezil hydrochloride and THg, we found that the levels of p-PI3K, p-Akt, and p-mTOR proteins in the hippocampal region of the rats were markedly elevated (p < 0.05). Concurrently, we observed that the expressions of BECLIN1 and LC3 proteins were also significantly reduced (p < 0.05) (Figures 9E-G). Notably, the high-dose THg group demonstrated the most pronounced therapeutic effect. These findings suggest that THg may regulate autophagy in brain tissue by activating the PI3K/Akt-mTOR signaling pathway.

## 4 Discussion

Vascular dementia (VaD) is a form of cognitive impairment resulting from various cerebrovascular injuries, including large-vessel stroke and microvascular dysfunction. VaD accounts for approximately 20% of dementia cases, making it the second most prevalent form of dementia after Alzheimer’s disease (28, 29). The incidence of VaD increases with age and the rising prevalence of cerebrovascular risk factors, driven by the accelerated aging of the Chinese population in recent years. Consequently, early diagnosis and effective interventions for VaD are essential. Recent studies suggest that THg reduces MDA levels in the brain tissue of VaD rats, increases SOD activity, scavenges free radicals, and promotes VEGF expression in infarcted brain tissue, thereby supporting its role in promoting angiogenesis to alleviate and repair cerebral ischemia. THg alleviates brain edema after cerebral ischemia by inhibiting thrombus formation, promoting Bcl-2 expression, and suppressing Bax expression in brain tissue, thereby enhancing brain cell tolerance to ischemia and hypoxia and facilitating brain repair (30, 31). However, the exact pharmacological mechanisms of THg in treating VaD remain unclear.

This study systematically demonstrated the pharmacological effects of THg on VaD and its potential therapeutic mechanisms and explored animal experiments and network pharmacology for the first time, emphasizes the interactions between active compounds, chemical targets, and signaling pathways. Seventy-six active compounds in THg and 825 direct target genes were identified in this study, suggesting that THg plays a multifaceted pharmacological role in the treatment of VaD. Beta-sitosterol, a naturally occurring plant sterol, is one of the primary components of THg in the treatment of VaD. Research indicates that beta-sitosterol prevents ischemic stroke by inhibiting signaling pathways associated with neuronal cell death, endoplasmic reticulum stress, and cholesterol overload (32).

Calycosin, extracted from Astragalus root, is a well-known phytoestrogen with diverse pharmacological effects. Studies have shown that calycosin improves neurological function and reduces neuronal death in rats with cerebral artery occlusion (33). Furthermore, studies have shown that calycosin mitigates damage caused by brain ischemia/reperfusion by suppressing ACSL4-mediated ferroptosis (34). Kaempferol, a major bioflavonoid found in various fruits, vegetables, and medicinal plants, exhibits antioxidant and neuroprotective properties that help prevent stroke and Alzheimer’s disease (35, 36). Studies have demonstrated that it enhances cell viability and reduces cell death in OGD/R-treated neurons by activating the Nrf2/SLC7A11/GPX4 signaling pathway. Quercetin, a natural flavonoid abundant in fruits, vegetables, and herbs, may offer protective effects against various diseases (37). Studies suggest that quercetin’s anti-inflammatory, anti-apoptotic, and antioxidant properties provide therapeutic potential for metabolic, cardiac, and neurological disorders (38). Additionally, recent studies propose that quercetin may alleviate brain ischemia/reperfusion injury by promoting microglia/macrophage M2 polarization via regulation of the PI3K/Akt/NF-κB signaling pathway (39).

Using the analysis tool Venny 2.1.0, we identified 69 overlapping targets of THg for the treatment of VaD. To further explore the potential targets of THg in VaD treatment, we conducted a protein–protein interaction (PPI) network analysis. Based on the results, we identified 14 core targets (degree ≥40), including TNF, IL-6, AKT1, IL-1β, CASP3, EGFR, JUN, APP, PTGS2, HIF1A, PPARG, MAPK3, CXCL8, and ICAM1. Following this, KEGG and GO enrichment analyses were performed.

GO functional analysis identified that responses to lipopolysaccharides and reduced oxygen levels are key biological processes involved in THg therapy for VaD. Lipopolysaccharide (LPS) is an endotoxin that plays a critical role in the outer membrane of Gram-negative bacteria and impacts human immune function. When bacteria enter the body, they release lipopolysaccharide (LPS), which enhances bacterial resistance to external toxins, such as antibiotics, while promoting inflammation and the release of harmful substances. Oxidative stress (OS), a hallmark of neurodegenerative diseases, results from an imbalance in the internal environment caused by the uncontrolled production of reactive oxygen species (ROS) (40, 41). Excessive ROS production leads to molecular oxidative damage, accelerating aging and contributing to conditions such as cancer, neurological disorders, and cardiovascular diseases (42).

.The therapeutic mechanism of THg in treating VaD is closely linked to signaling pathways, as revealed by the KEGG pathway enrichment analysis. The AGE-RAGE, TNF, PI3K-Akt, and MAPK signaling pathways, along with several other related pathways, were among the top 10 identified out of 143 pathways discovered through KEGG enrichment analysis. Consequently, the PI3K-Akt signaling pathway was selected for further investigation to explore the mechanism through which THg intervenes in VaD. The PI3K/Akt-mTOR signaling pathway is present in most eukaryotic cells and plays a critical role in regulating a wide range of biological processes, including autophagy, cell division, proliferation, apoptosis, and synaptic plasticity. As a key regulator of autophagy, activation of this pathway inhibits the autophagic process (43). Phosphoinositide 3-kinase (PI3K) is the primary initiator of the PI3K/Akt-mTOR signaling pathway. The PI3K family is composed of intracellular kinases specialized in inositol lipids, capable of generating the second messenger phosphoinositides. This family regulates various cellular processes, including cell growth, proliferation, metabolism, migration, and secretion. Abnormal PI3K signaling is frequently implicated in common human diseases, including cancer, immunological disorders, neurological conditions, and cardiovascular diseases (44). Various neurotrophic factors and growth stimuli can trigger PI3K activation. The PI3K/Akt pathway is activated when PI3K generates the second messenger PIP3 on the plasma membrane, which subsequently activates downstream AKT signaling molecules. Activated AKT triggers downstream cascades, phosphorylating a series of substrates that mediate cell growth, proliferation, cell cycle progression, and glucose metabolism. One of the key substrates is mTOR (45). mTOR, a member of the PIKK (PI3K-related kinase) family, forms two distinct complexes: mTOR complex 1 (mTORC1) and mTOR complex 2 (mTORC2). Once activated, mTORC1 regulates various biological processes, such as cell division and protein synthesis, and catalyzes the phosphorylation of autophagy-initiating proteins, thereby inhibiting autophagy formation (46). Among the other pathways revealed by KEGG results, we found that the MAPK and TNF signaling pathways are also associated with the therapeutic mechanisms of THg in treating VaD. The MAPK pathway consists of extracellular signal-regulated kinase (ERK), c-Jun N-terminal kinase (JNK), and p38 MAPK, which play a central regulatory role in maintaining neuronal autophagy balance and cell survival. Among these, the ERK signaling transduction pathway positively regulates the key molecular mechanisms of autophagosome formation through the cascade activation of its downstream effectors, mTORC1 and ULK1.The TNF (tumor necrosis factor) signaling pathway is a key regulator of cellular processes, including inflammation, apoptosis, and autophagy. By binding to TNF receptor 1 (TNFR1) or TNFR2, it exerts its effects. Under pathophysiological conditions such as nutrient deprivation, it activates the JNK signaling cascade downstream and initiates the autophagy cascade by specifically phosphorylating the Bcl-2 protein, thereby releasing Beclin-1 to initiate autophagosome formation. The p38 MAPK signaling module exhibits dual regulatory characteristics in autophagy regulation: on one hand, it can promote autophagy by activating autophagy-related genes (ATGs); on the other hand, it may suppress autophagy activity through the mTORC1-dependent pathway.

This study utilized a modified 2-vessel occlusion (2-VO) method to establish a vascular dementia (VaD) model. The 2-VO method, involving bilateral carotid artery occlusion in rats, is a well-established and widely used model for studying the mechanisms of chronic cerebral hypoperfusion (47). The modified 2-VO method differs from the traditional approach primarily in the timing of vessel occlusion. Seven days after ligation and suturing of one carotid artery, the other artery is occluded. The modified 2-VO method significantly improves the success and survival rates of animals compared to the original method. Therefore, the modified 2-VO method was employed to model vascular dementia (48).

Rats that recovered well post-surgery and regained consciousness were selected for further evaluation using the Zea-Longa grading system. The rats were divided into groups, and their spatial learning and memory abilities were assessed using the Morris water maze. The data indicated that compared to the Sham group, rats in the VaD group exhibited significantly longer escape latencies, fewer platform crossings, and reduced time spent in the target quadrant. Rats treated with high doses of THg, notably, showed significant improvement in spatial learning ability.

Located between the thalamus and the medial temporal lobe, the hippocampus serves as a crucial framework for linking experiences of location and time, which are critical for episodic memory and spatial navigation (49, 50). The hippocampus consists of three main structures based on area and function: the CA3 region, the CA1 region, and the dentate gyrus (DG) (51, 52). Pyramidal neurons in the CA1 region of the hippocampus act as crucial output nodes in its memory circuitry, exhibiting various projection sequences. Chronic cerebral hypoperfusion-induced ischemic damage to hippocampal neurons can result in significant impairments in memory and learning. Because CA1 pyramidal neurons are susceptible to ischemic injury, this study employed hematoxylin and eosin (HE) staining to observe pathological abnormalities in the CA1 region of the hippocampus across different groups of rats. HE staining revealed that compared to the Sham group, rats in the VaD group exhibited neuronal degeneration in the CA1 region of the hippocampus after prolonged cerebral underperfusion. THg treatment significantly improved the structure of hippocampal neurons in VaD rats, including an increased number of cells and largely intact cellular architecture. There was a significant improvement in nerve integrity. These results were consistent with those obtained from the Morris water maze (MWM) evaluation. These findings suggest that THg can improve hippocampal neuronal cell damage, contributing to improved cognitive function in VaD rats. This emphasizes the potential of THg therapy for VaD and highlights its therapeutic effectiveness.

Our experiments focused on autophagy by validating the expression of two classical autophagy proteins, BECLIN1 and LC3. Immunohistochemistry was employed to detect the autophagy proteins. BECLIN1, an evolutionarily conserved protein, plays a crucial role as a key regulator in the development of autophagosomes (7). When autophagy is activated, cytoplasmic LC3 (LC3-I) binds to phosphatidylethanolamine, undergoing lipidation to form LC3-II. LC3-II serves as a critical molecular marker for autophagy initiation and directly correlates with the number of autophagic vesicles. Autophagy levels are typically assessed by the LC3-II/LC3-I ratio. The degree of autophagy is correlated with the number of autophagic vesicles, as indicated by the expression ratio of LC3-II to LC3-I (53). THg significantly reduced the expression levels of LC3 and BECLIN1 proteins in the hippocampal CA1 region of VaD model rats, with the most pronounced effect observed in the high-dose THg group. These results suggest that THg effectively inhibits autophagy in the hippocampal neurons of VaD model rats, as demonstrated by reduced BECLIN1 and LC3 expression.

**Figure 10:**
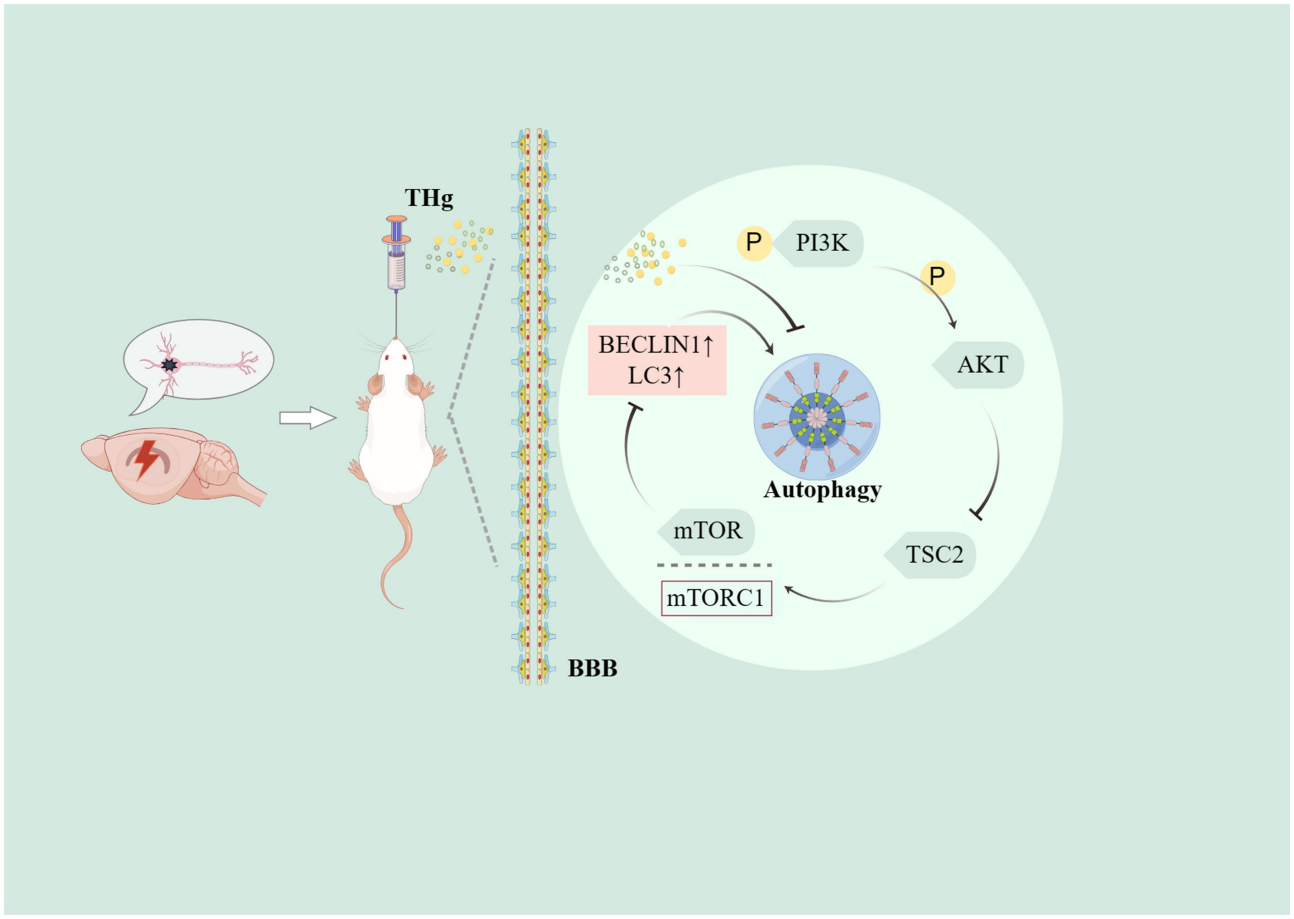
The diagram illustrates that THg inhibit autophagy in hippocampal neuronal cells of VD rats by activating the PI3K/AKT/mTOR signalling pathway, thereby ameliorating cognitive dysfunction in VD rats.

This study focused on the PI3K/Akt-mTOR signaling pathway, which was identified through network pharmacology analysis. The PI3K/AKT pathway regulates proliferation, differentiation, autophagy, and apoptosis in neural cells, making it a widely studied target for neuroprotective mechanisms in brain ischemia (54). Phosphorylated PI3K directly or indirectly activates AKT, resulting in the formation of p-AKT. AKT activation is crucial for neuronal survival in brain ischemia/reperfusion injury (55), driving the activation of the downstream core component mTOR complex 1 (mTORC1). Cell growth and division rely on complex metabolic regulatory mechanisms, enhancing the synthesis of proteins, lipids, and nucleic acids while suppressing catabolic pathways like autophagy. The catalytic subunit of the mTOR complex, mTORC1, serves as a central hub regulating these metabolic processes (46). Western blotting results showed that THg significantly activated the PI3K/AKT/mTOR signaling pathway and inhibited BECLIN1 and LC3 expression compared to the model group, with the most pronounced effects observed in the high-dose THg group. These findings suggest that THg inhibits the expression of autophagy proteins BECLIN1 and LC3 in the hippocampal CA1 region of VaD rats, likely via activation of the PI3K/AKT/mTOR signaling pathway (Figure 10).

The above results suggest that THg may regulate neuronal autophagy in the hippocampal region induced by chronic cerebral hypoperfusion through activation of the PI3K/AKT/mTOR signaling pathway, thereby alleviating neuronal damage and improving cognitive dysfunction in VaD rats.

## 5 Conclusion

According to the network pharmacology analysis in this study, the main active ingredients of THg for VaD include quercetin, kaempferol, β-sitosterol, and tyrosine. Among the 69 identified targets of THg for VaD treatment, TNF (Tumor Necrosis Factor), IL-6 (Interleukin-6), IL-1β (Interleukin-1 beta), and AKT were the four primary targets. The KEGG enrichment analysis revealed that The PI3K/AKT signaling pathway plays a central role in the mechanism of action of THg for VaD treatment. Animal experiments demonstrated that THg eenhances cognitive function, improves spatial learning, and memory in VaD rats by reducing neuronal necrosis, inhibiting autophagy-related proteins BECLIN1 and LC3, and repairing cellular damage in hippocampal CA1 neurons. These effects may be associated with activation of the PI3K/Akt-mTOR signaling pathway.

## Data Availability

The original contributions presented in the study are included in the article/Supplementary material, further inquiries can be directed to the corresponding author.

## Footnotes

^1^ https://pubchem.ncbi.nlm.nih.gov/

^2^ http://www.swisstargetprediction.ch/

^3^ https://www.genecards.org

^4^ https://www.omim.org

^5^ https://ctdbase.org

^6^ https://bioinfogp.cnb.csic.es/tools/venny

^7^ https://cn.string-db.org

^8^ https://david.ncifcrf.gov

^9^ https://www.bioinformatics.com.cn/en

## Glossary

VaD: Vascular Dementia
Sham: Pseudosurgery group
THg: Tongqiao Huashuan Granules
L-THg: Low-dose Tongqiao Huashuan Granules Group
M-THg: Medium-dose Tongqiao Huashuan Granules Group
H-THg: High-dose Tongqiao Huashuan Granules Group
TCMSP: Traditional Chinese Medicine Systems Pharmacology Database and Analysis Platform
OB: Bioavailability
DL: Drug-likeliss
OMIM: Online Mendelian Inheritance in Man
CTD: Comparative Toxicogenomics Database
STRING: Search Tool for the Retrieval of Interacting Genes/Proteins
DAVID: The Database for Annotation, Visualization and Integrated Discovery
GO: Gene ontology
KEEG: Kyotoencyclopedia of genes and genome
PI3K: Phosphatidylinositide 3-kinase
AKT: Protein kinase B
mTOR: Mammalian target of rapamycin
LC3: Microtubule-associated proteins light chain 3
TNF: Tumor Necrosis Factor
IL-6: Interleukin-6
IL-1β: Interleukin-1 beta
