# Supplementary figures and images for: Investigation of Tongqiao Huashuan Granules’ effect on hippocampal neuron autophagy in vascular dementia rats via the PI3K/Akt-mTOR signaling pathway using network pharmacology and experimental validation
Source: Front Neurol. 2025 Mar 21;16:1555411. doi: 10.3389/fneur.2025.1555411 (PMC11968393; doi:10.3389/fneur.2025.1555411)

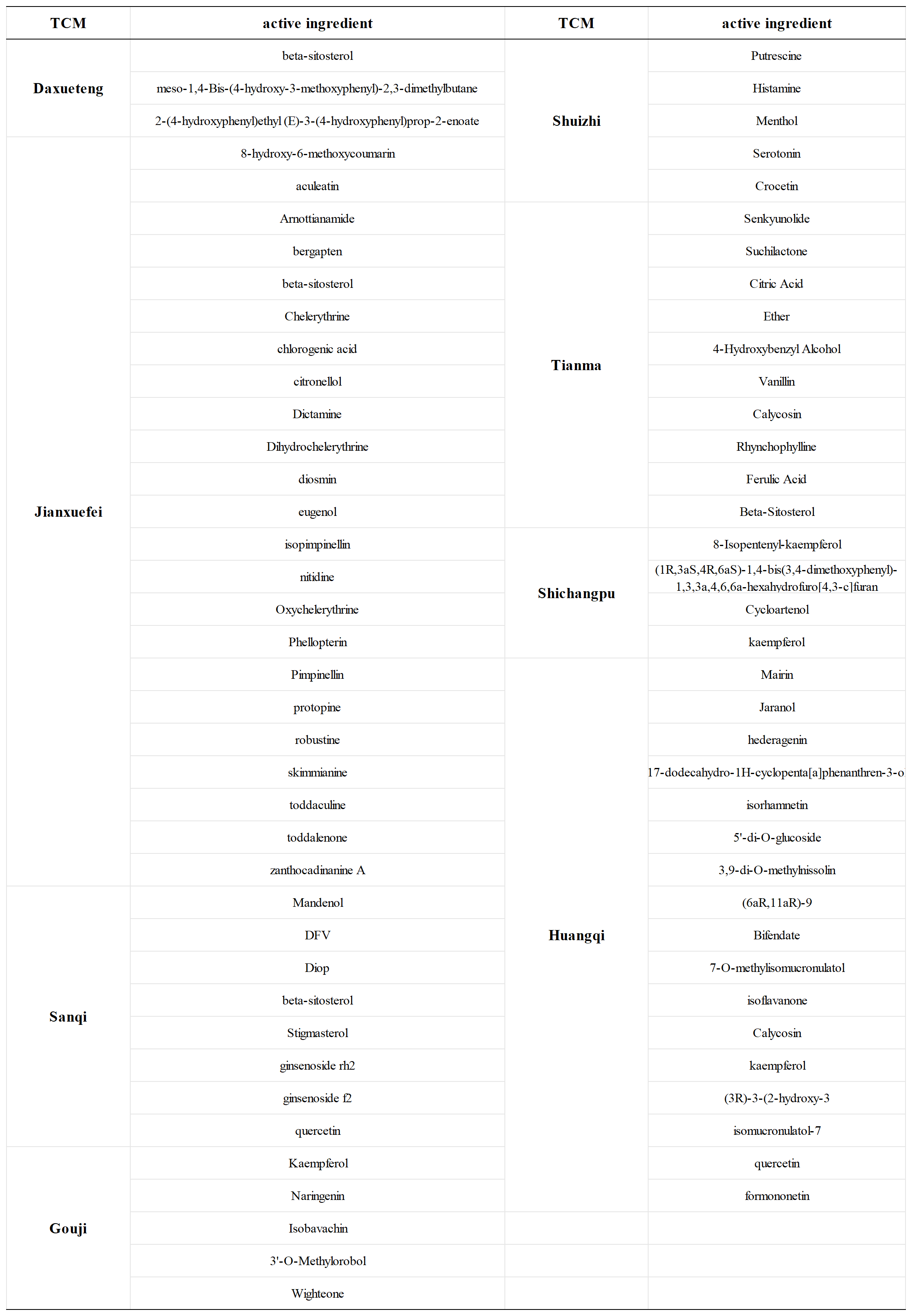

Supplement: Supplementary file 1 [file Image_1.JPEG]

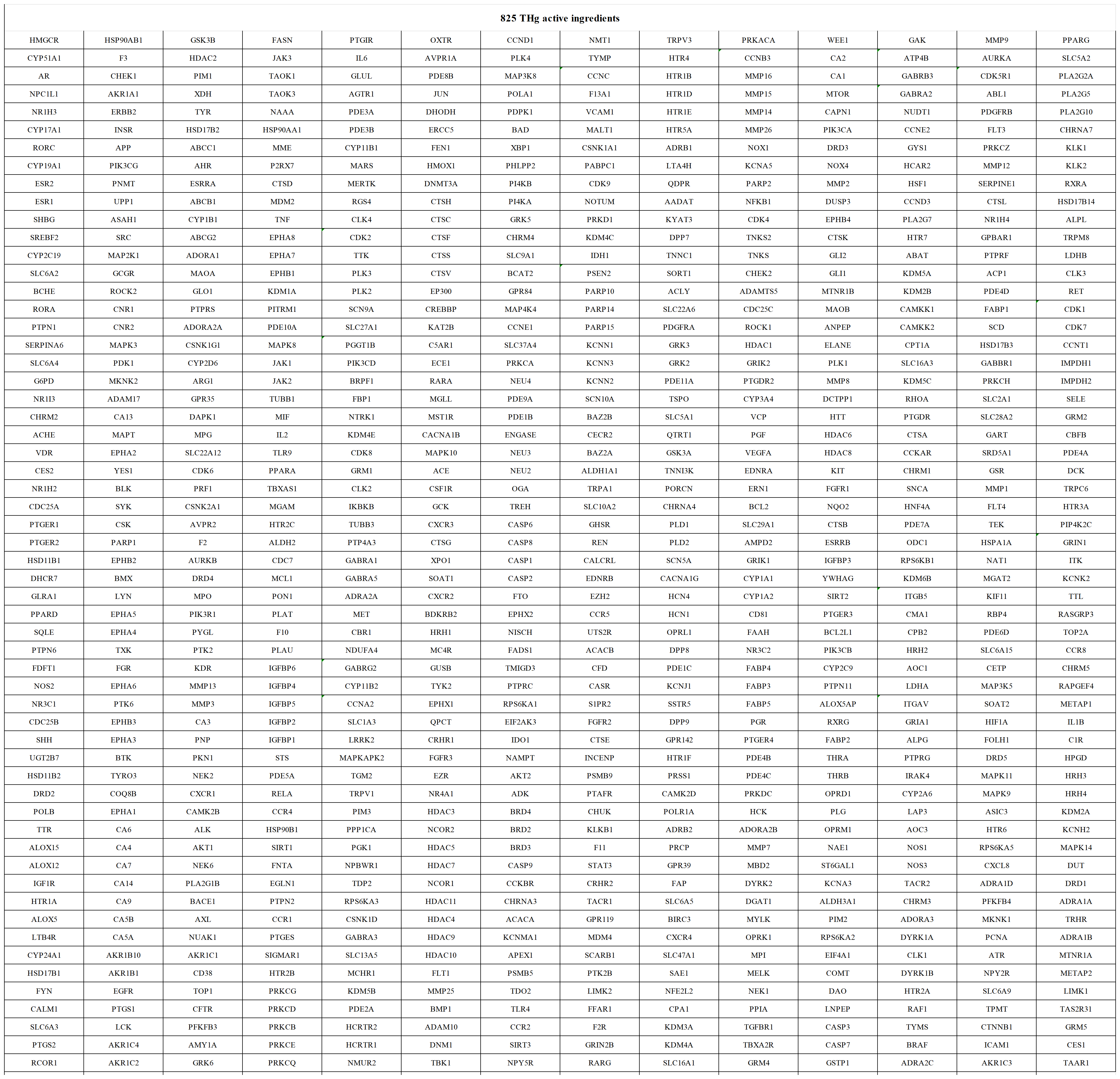

Supplement: Supplementary file 2 [file Image_2.JPEG]

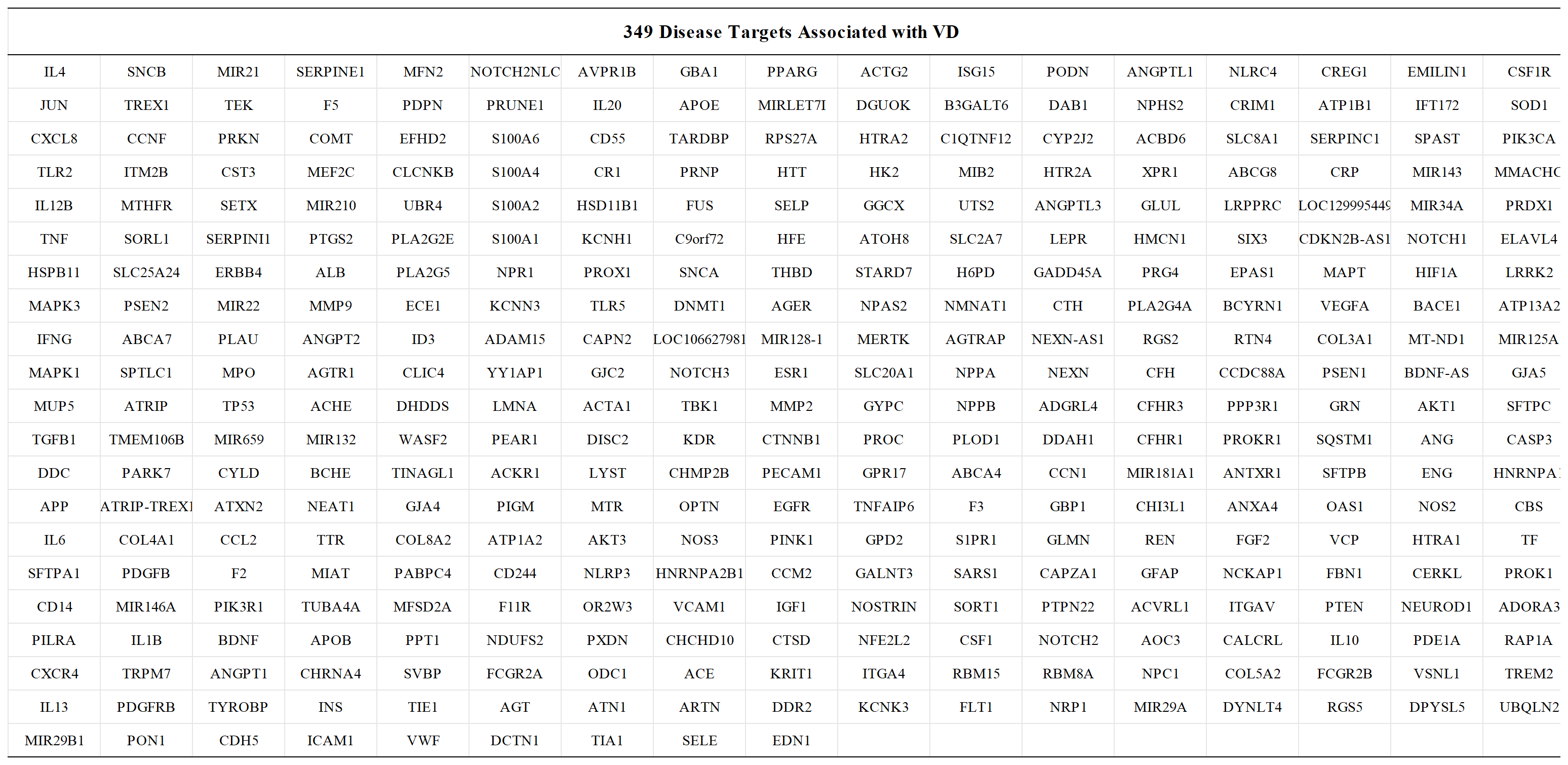

Supplement: Supplementary file 3 [file Image_3.JPEG]

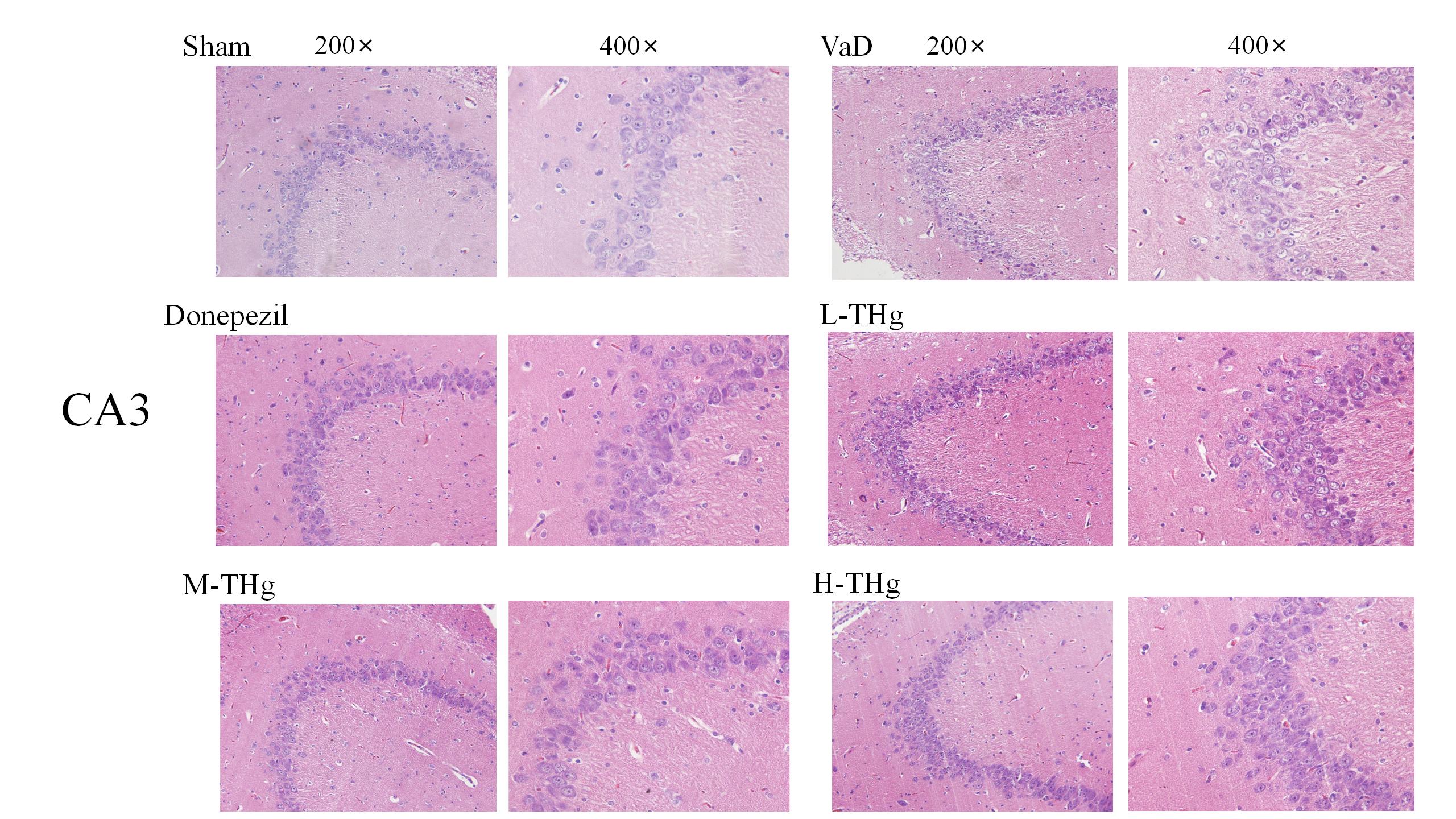

Supplement: Supplementary file 4 [file Image_4.JPEG]

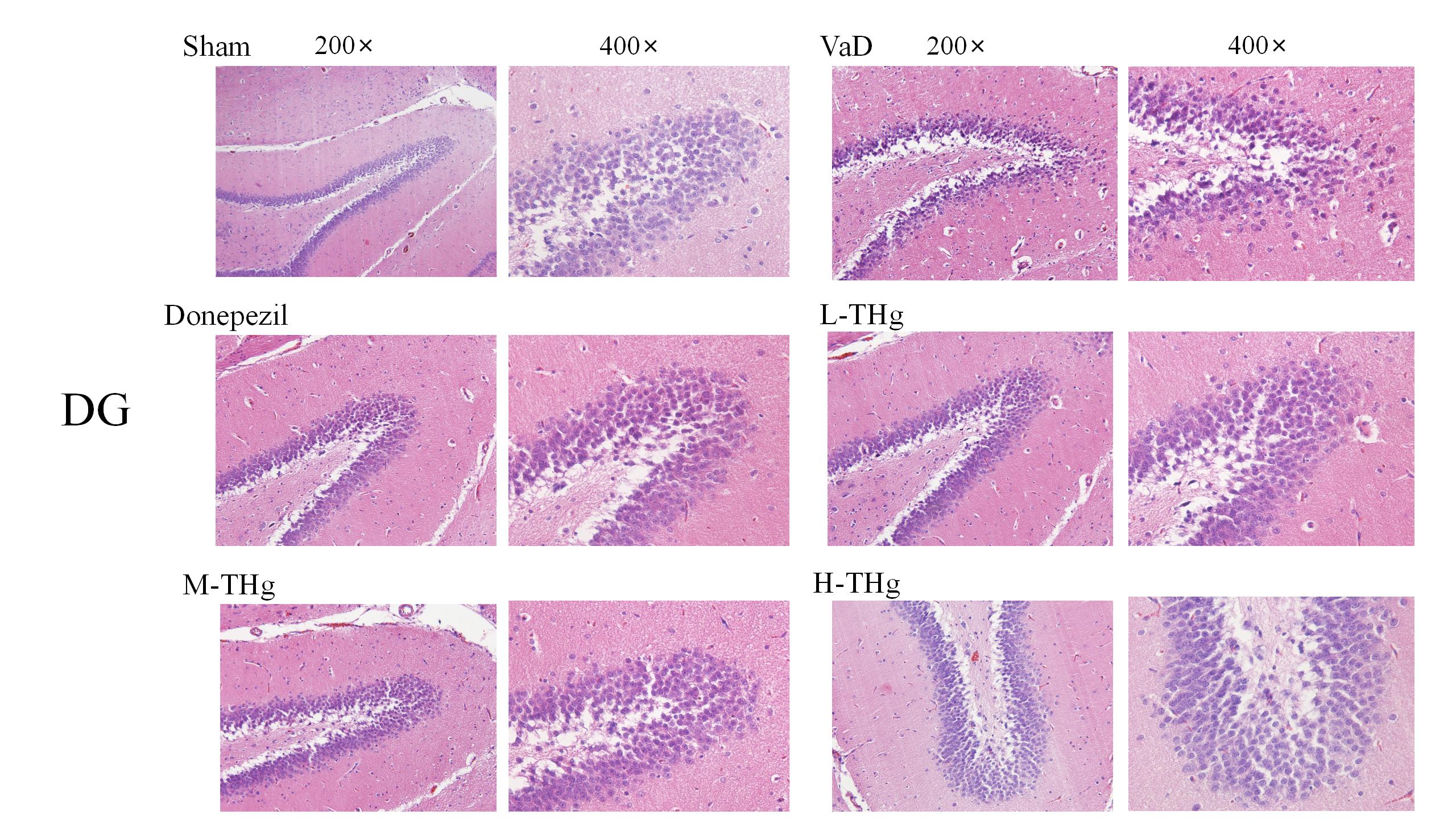

Supplement: Supplementary file 5 [file Image_5.JPEG]

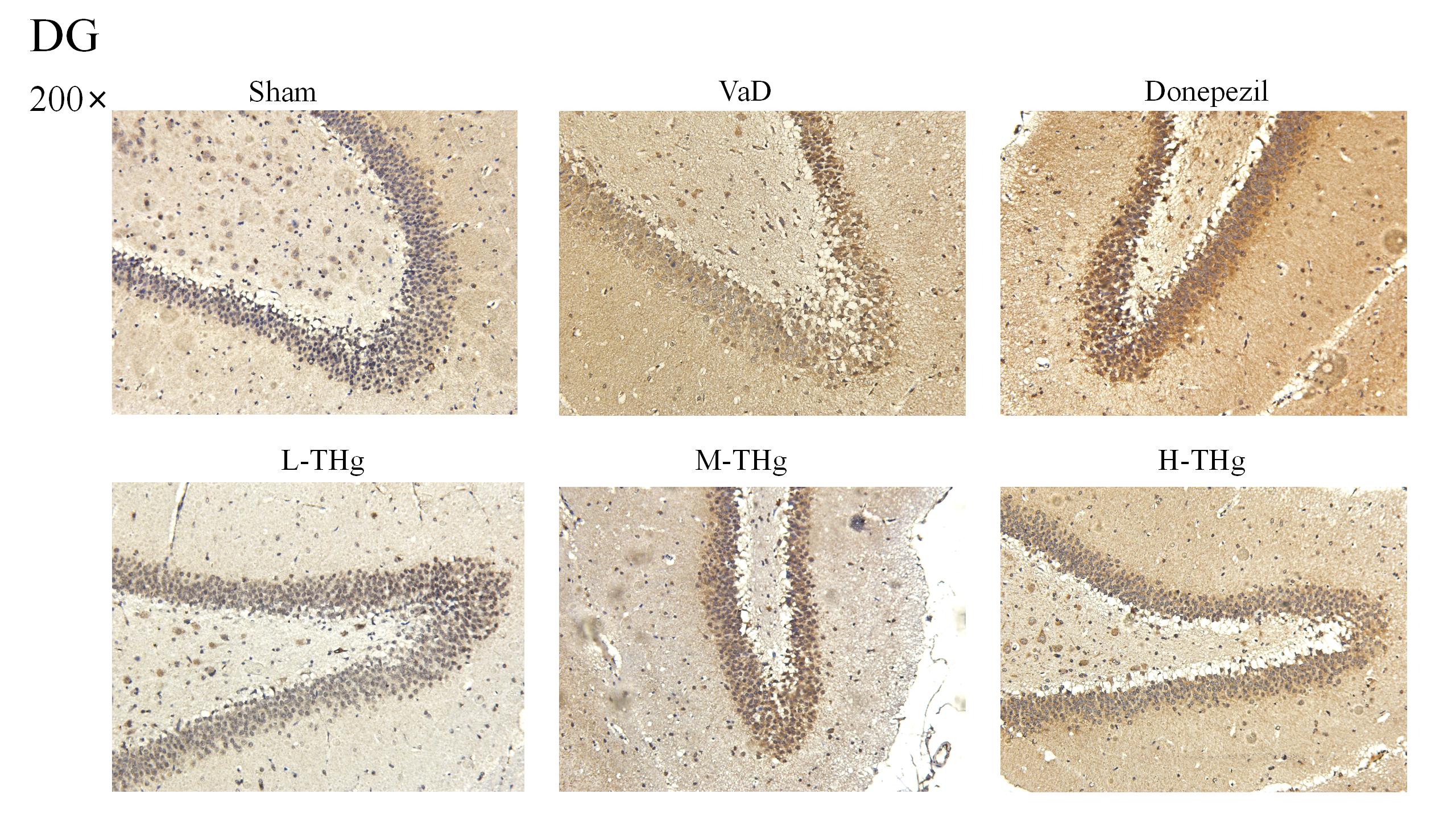

Supplement: Supplementary file 6 [file Image_6.JPEG]

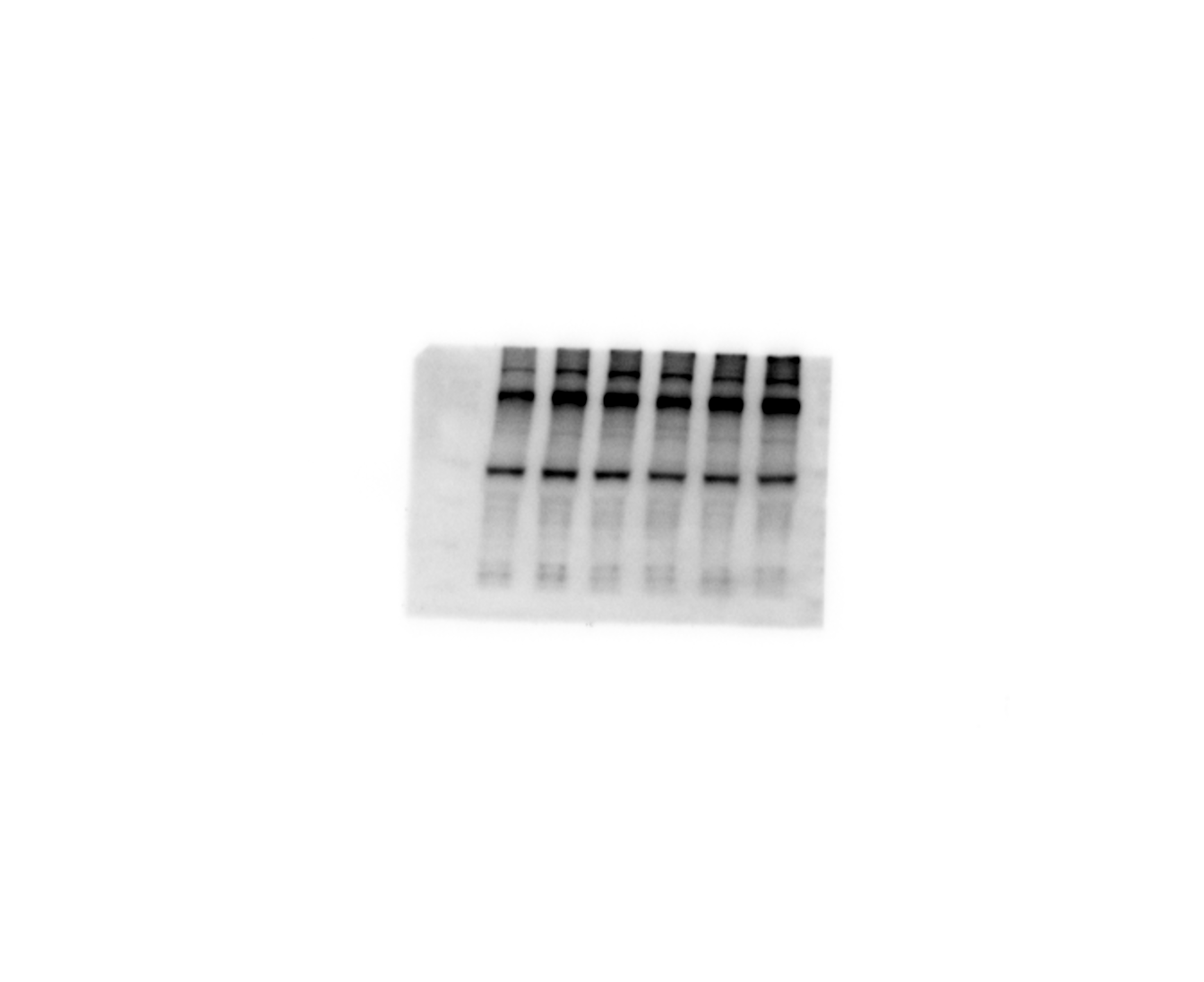

Supplement: Supplementary file 7 [file Data_Sheet_1.ZIP › BECLIN1,LC3/beclin1/1.Tif]

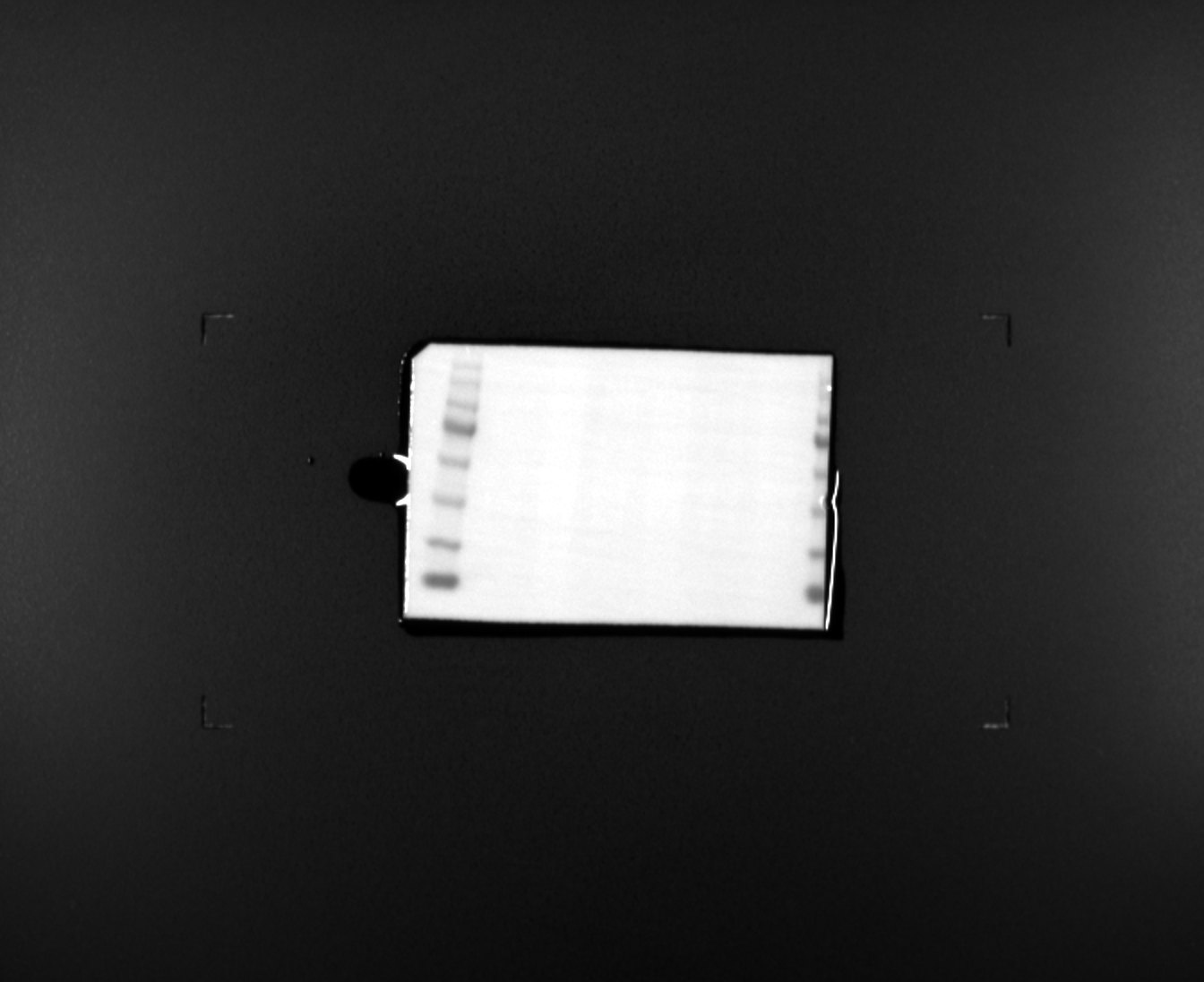

Supplement: Supplementary file 7 [file Data_Sheet_1.ZIP › BECLIN1,LC3/beclin1/1-t.Tif]

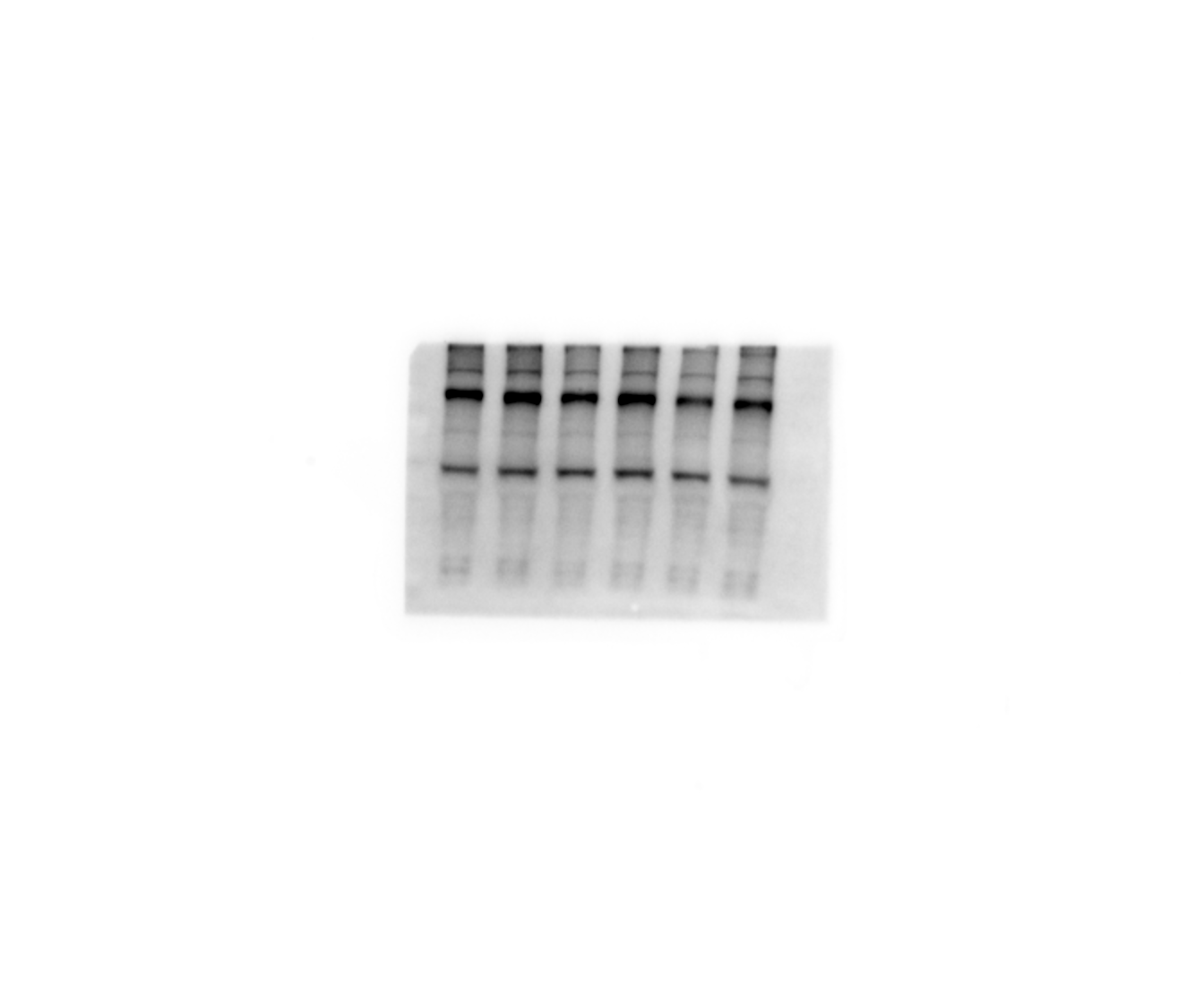

Supplement: Supplementary file 7 [file Data_Sheet_1.ZIP › BECLIN1,LC3/beclin1/2.Tif]

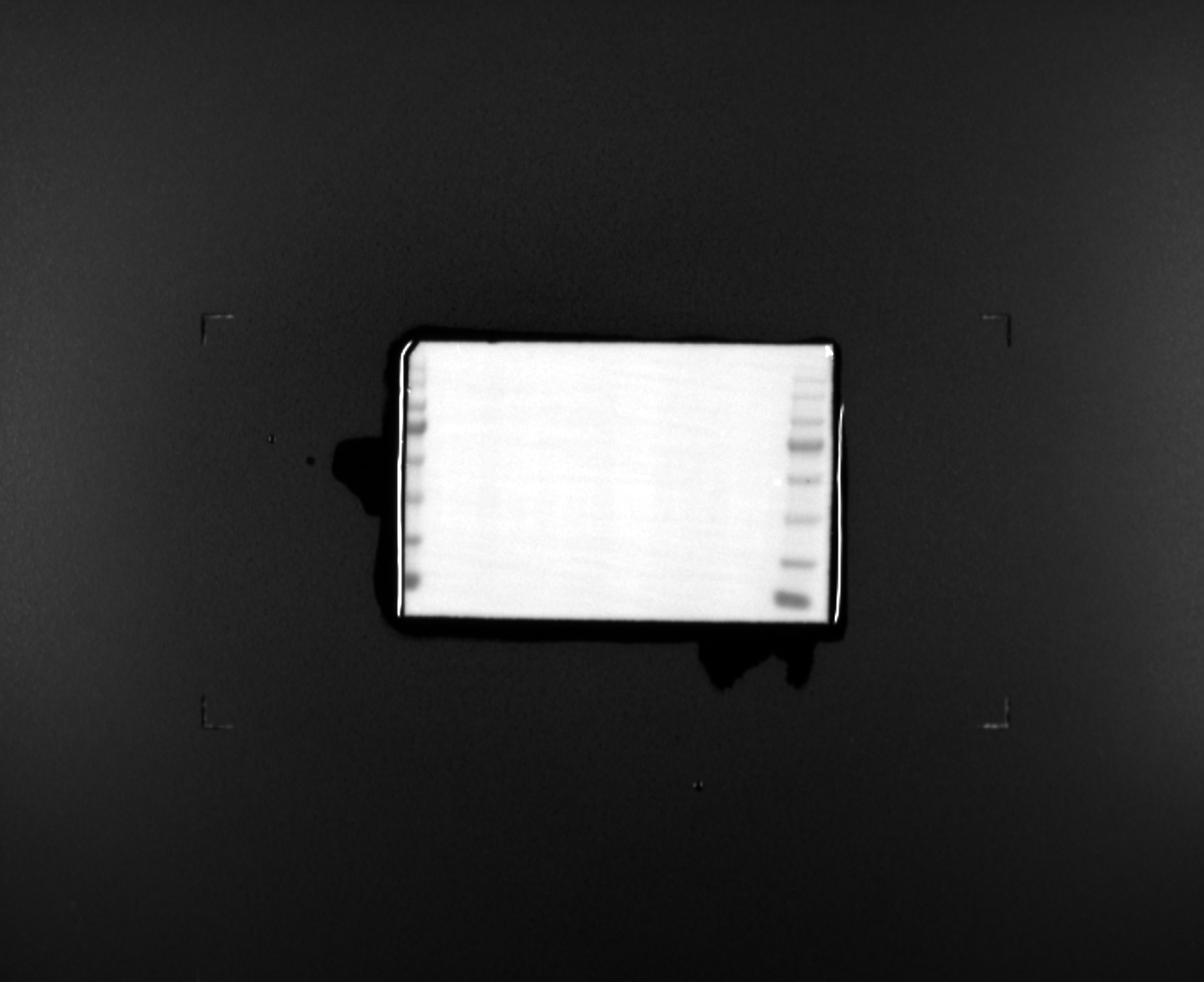

Supplement: Supplementary file 7 [file Data_Sheet_1.ZIP › BECLIN1,LC3/beclin1/2-t.Tif]

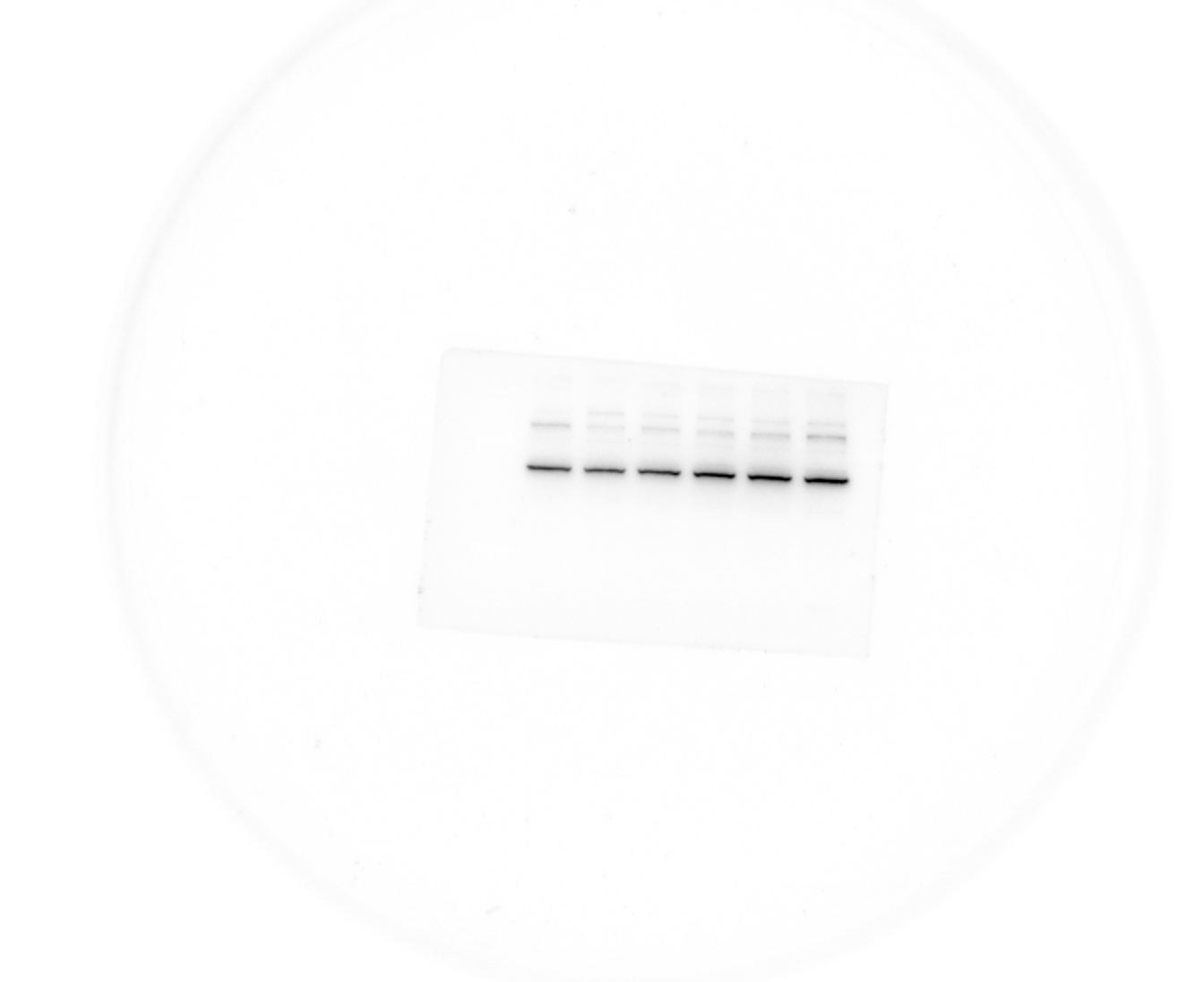

Supplement: Supplementary file 7 [file Data_Sheet_1.ZIP › BECLIN1,LC3/beclin1/3.Tif]

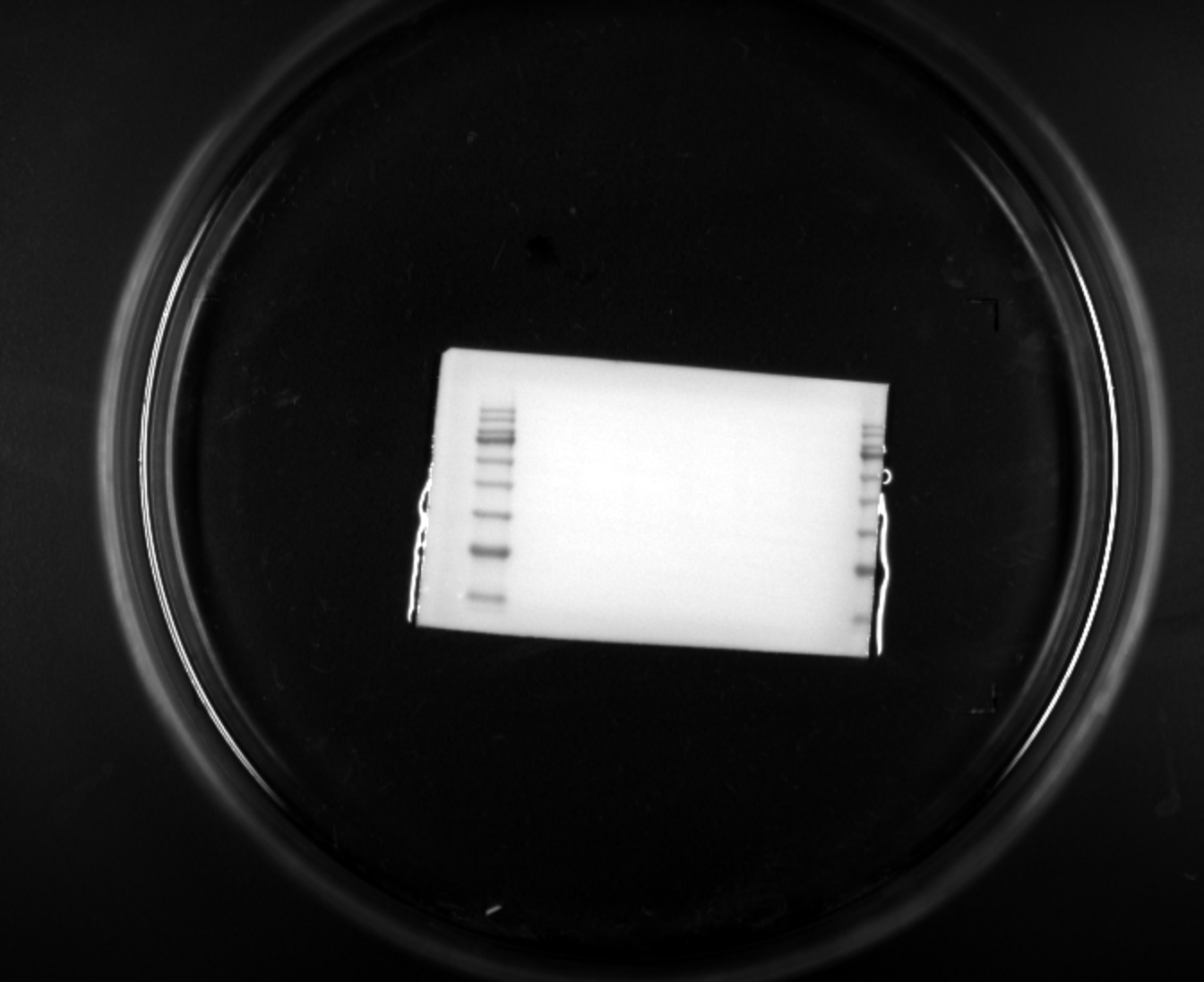

Supplement: Supplementary file 7 [file Data_Sheet_1.ZIP › BECLIN1,LC3/beclin1/3-t.Tif]

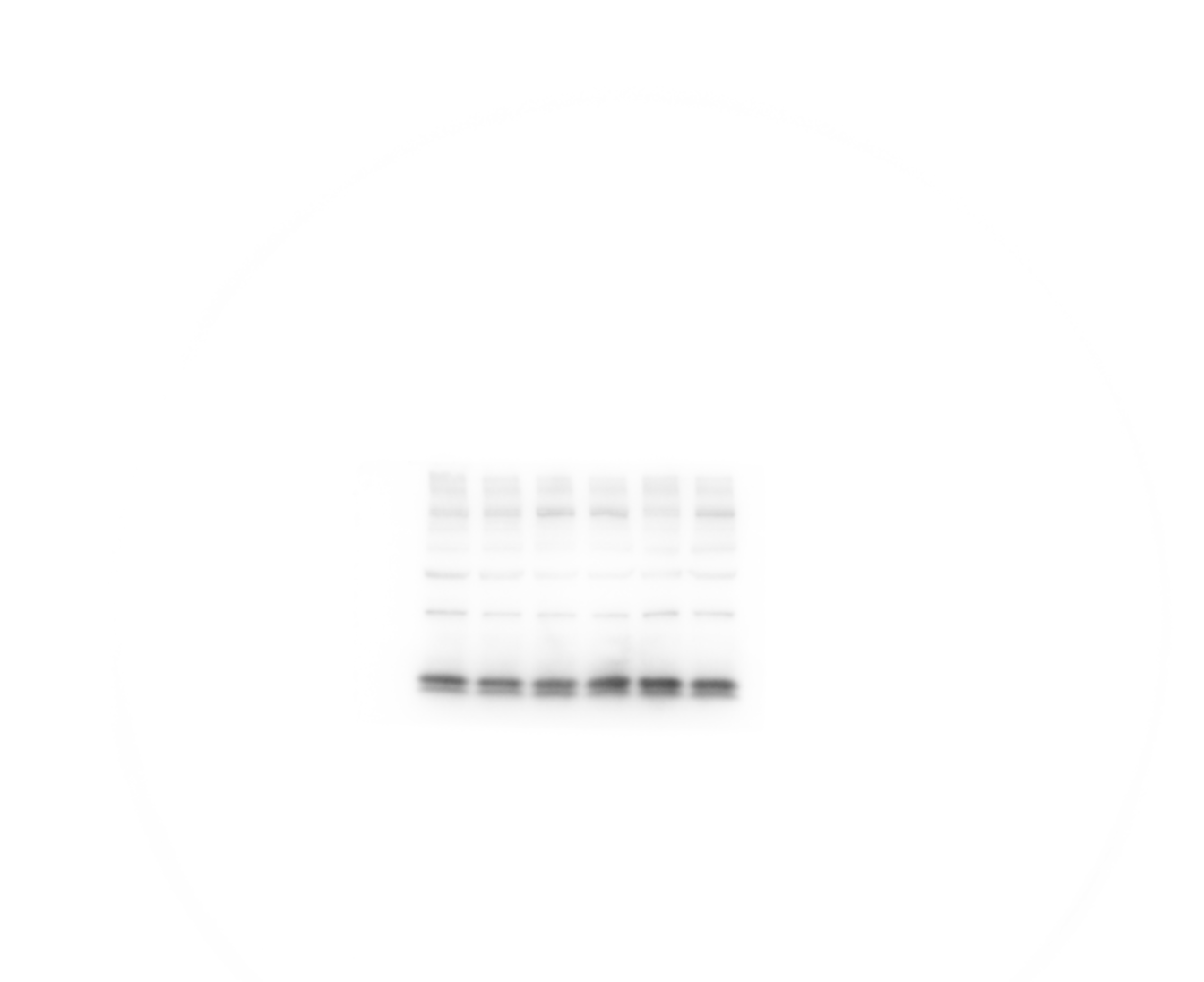

Supplement: Supplementary file 7 [file Data_Sheet_1.ZIP › BECLIN1,LC3/lc3/1.Tif]

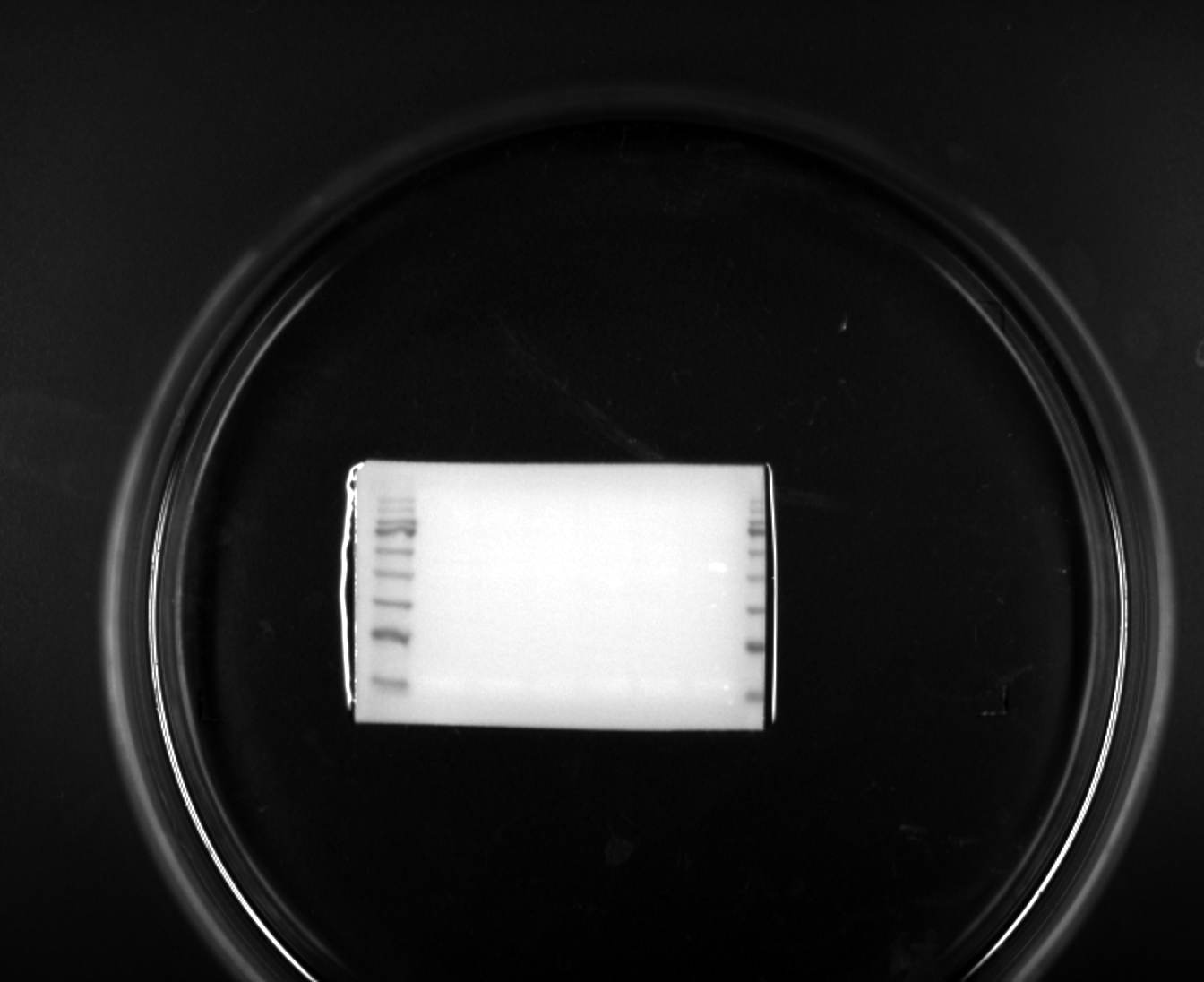

Supplement: Supplementary file 7 [file Data_Sheet_1.ZIP › BECLIN1,LC3/lc3/1-t.Tif]

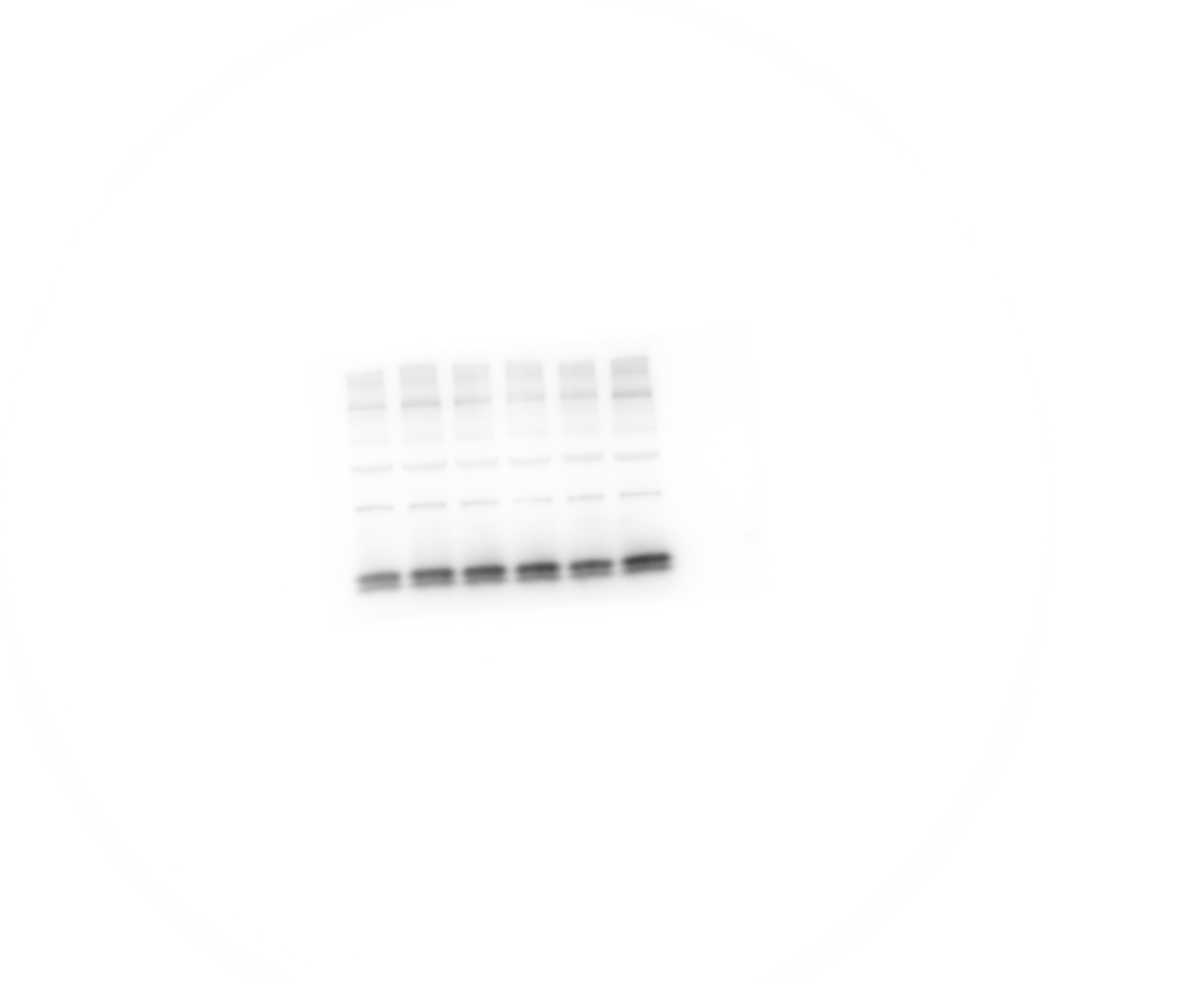

Supplement: Supplementary file 7 [file Data_Sheet_1.ZIP › BECLIN1,LC3/lc3/2.Tif]

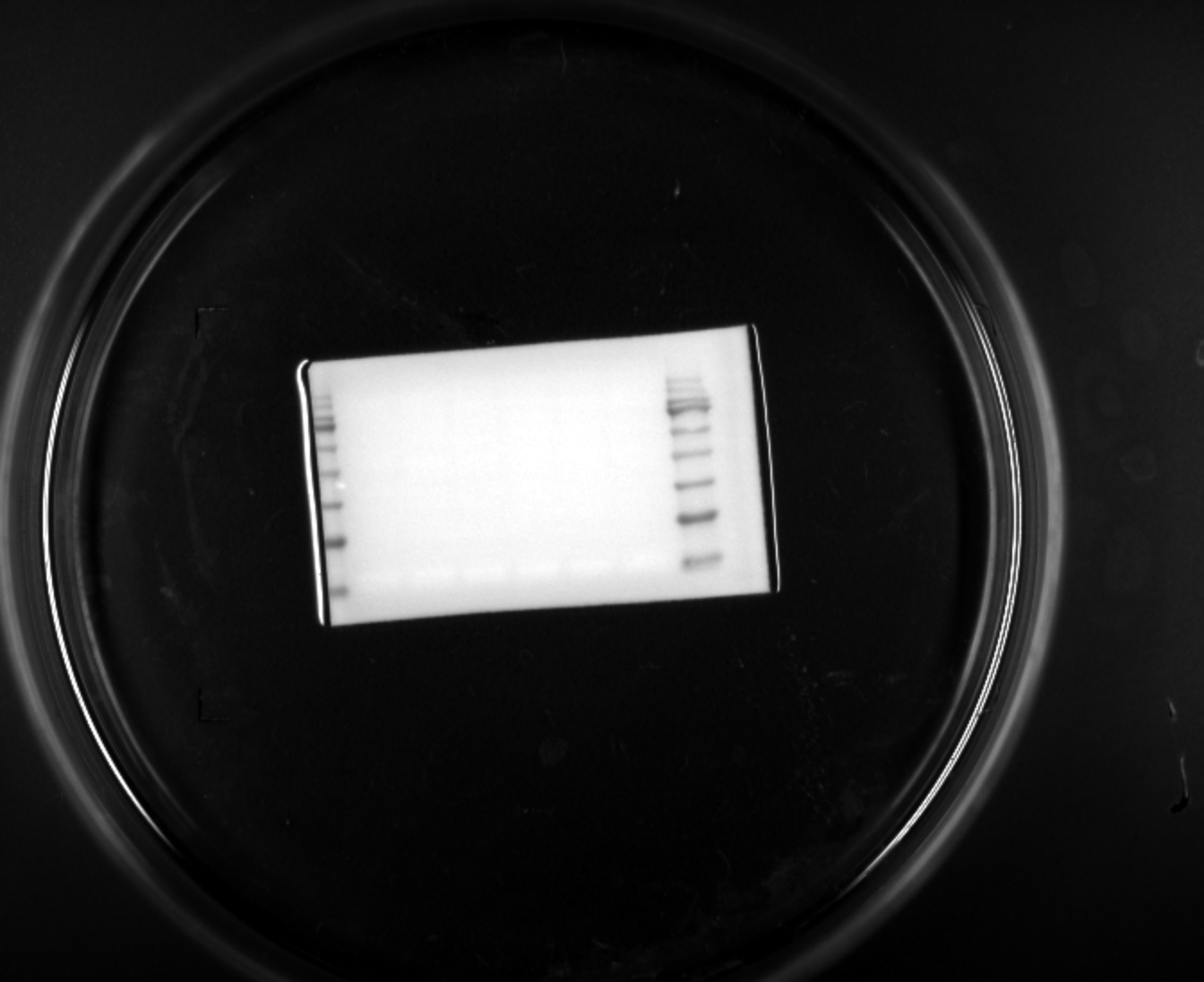

Supplement: Supplementary file 7 [file Data_Sheet_1.ZIP › BECLIN1,LC3/lc3/2-t.Tif]

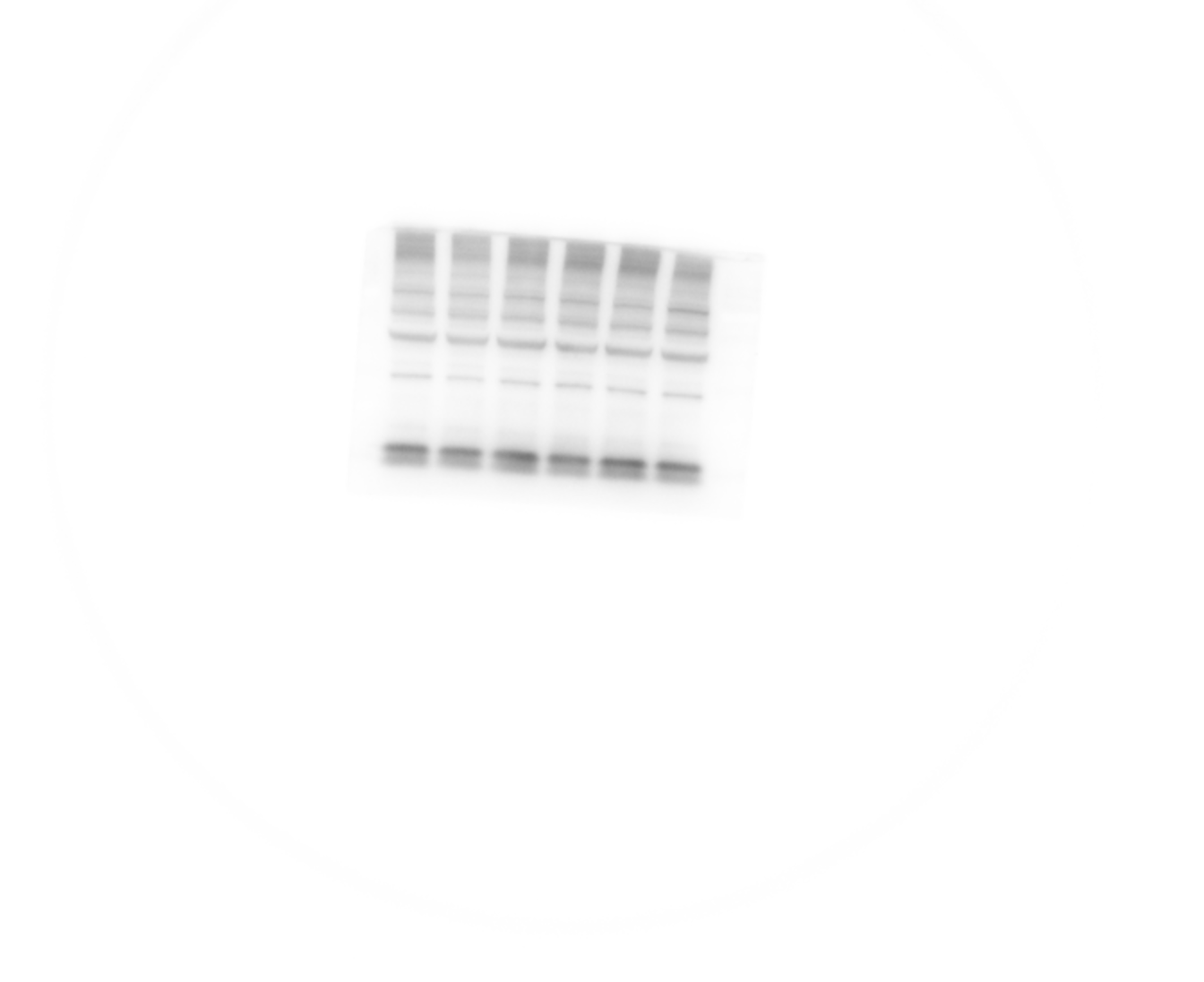

Supplement: Supplementary file 7 [file Data_Sheet_1.ZIP › BECLIN1,LC3/lc3/3.Tif]

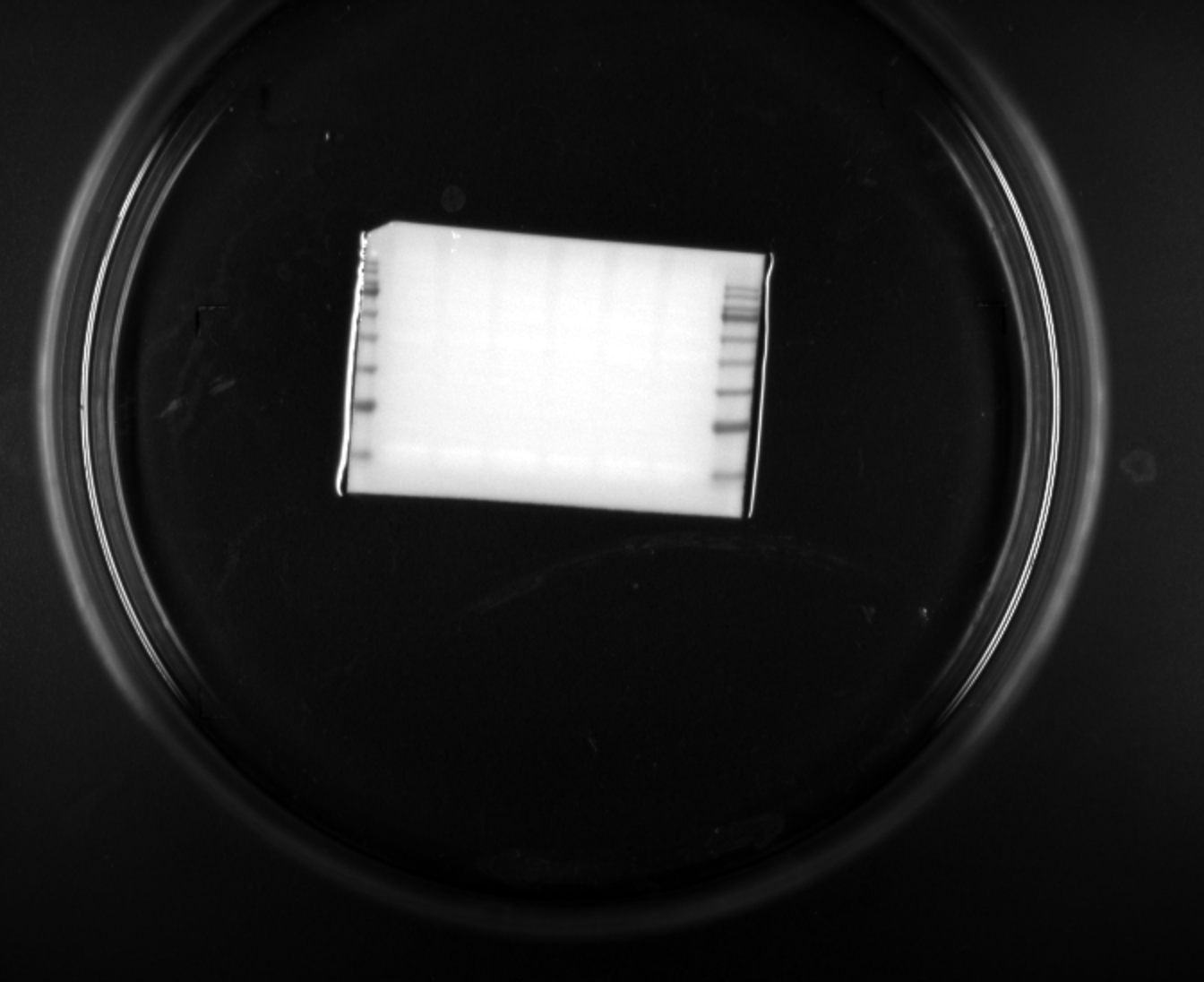

Supplement: Supplementary file 7 [file Data_Sheet_1.ZIP › BECLIN1,LC3/lc3/3-t.Tif]

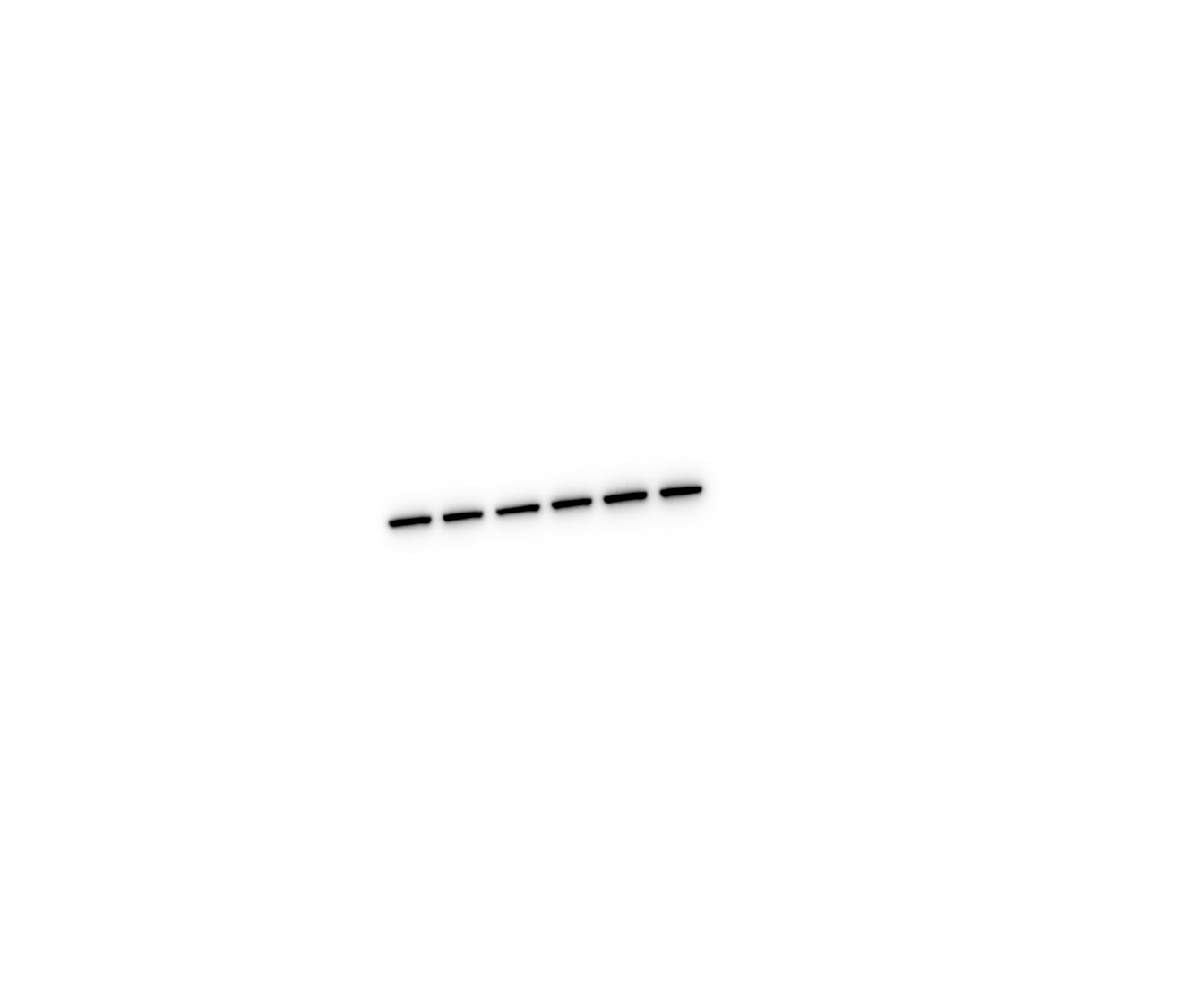

Supplement: Supplementary file 7 [file Data_Sheet_1.ZIP › BECLIN1,LC3/a┬-actin/1.Tif]

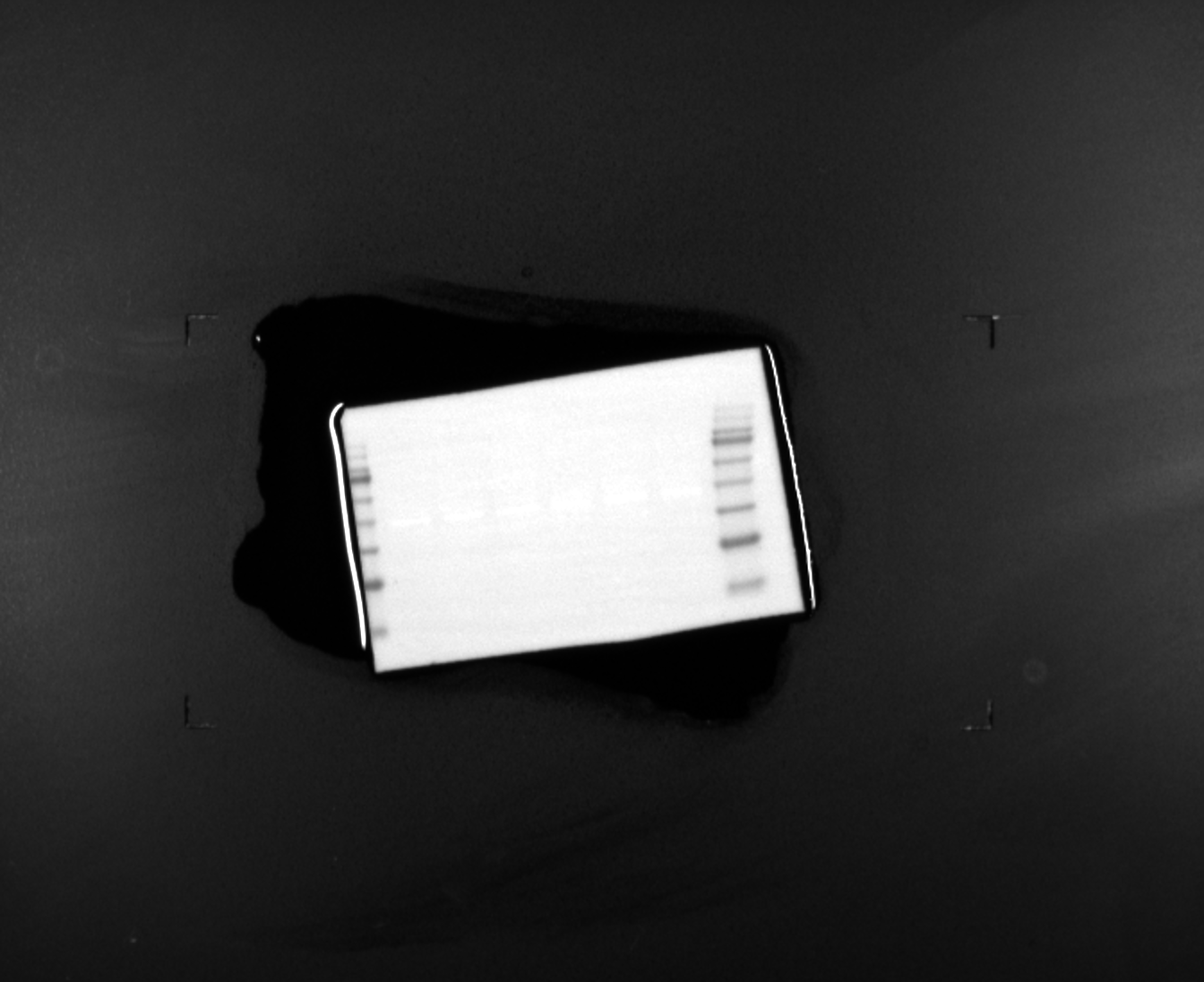

Supplement: Supplementary file 7 [file Data_Sheet_1.ZIP › BECLIN1,LC3/a┬-actin/1-t.Tif]

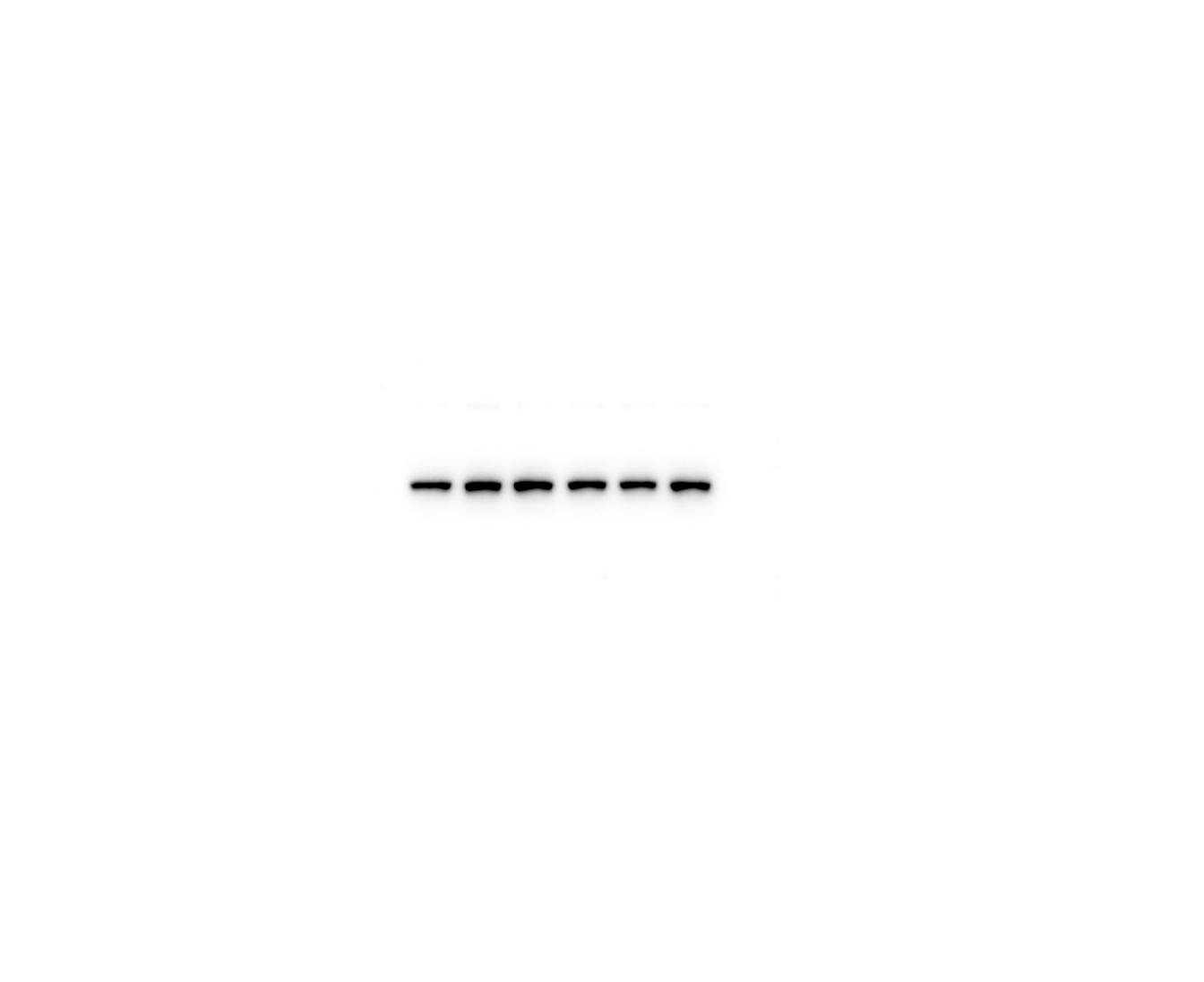

Supplement: Supplementary file 7 [file Data_Sheet_1.ZIP › BECLIN1,LC3/a┬-actin/2.Tif]

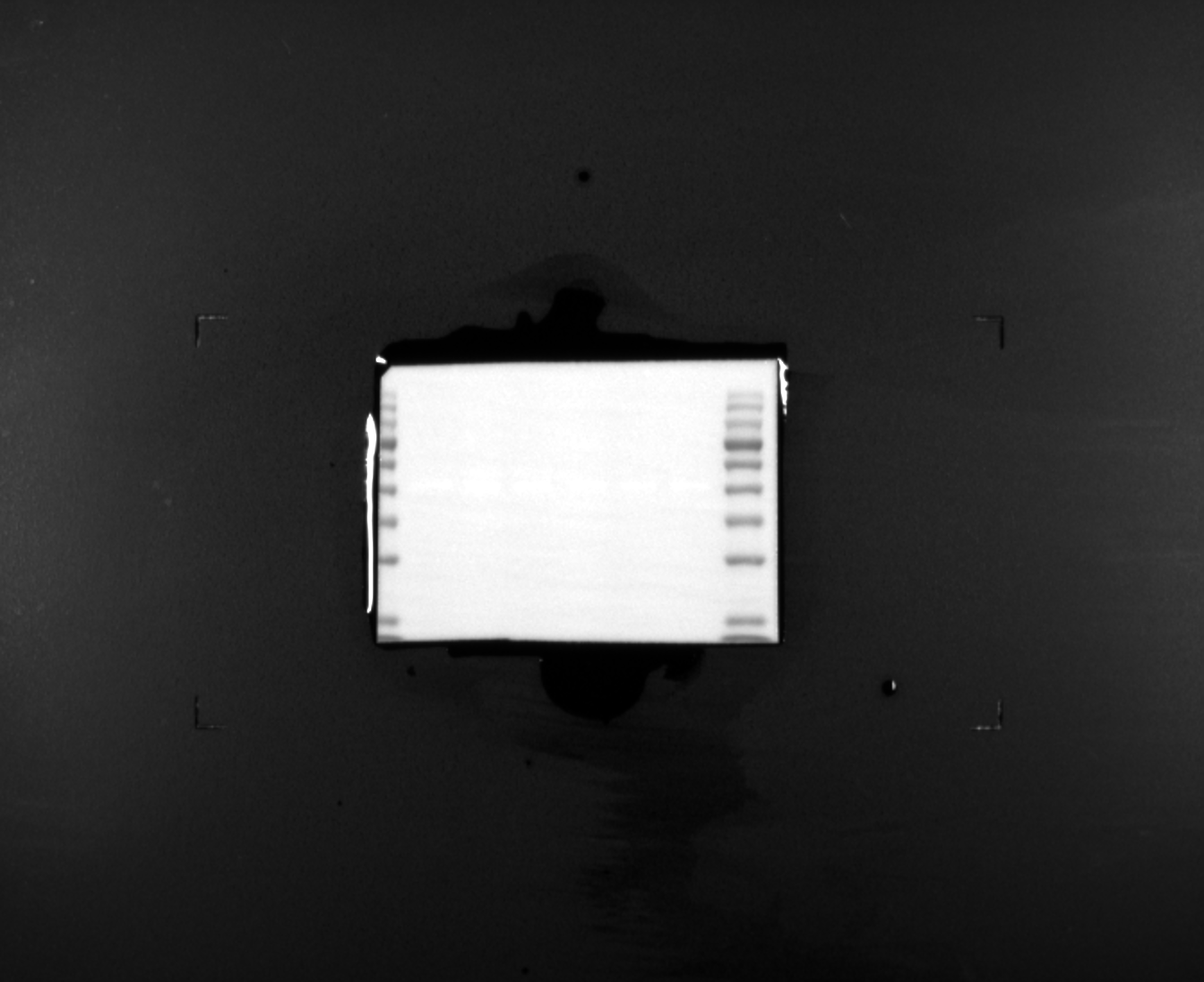

Supplement: Supplementary file 7 [file Data_Sheet_1.ZIP › BECLIN1,LC3/a┬-actin/2-t.Tif]

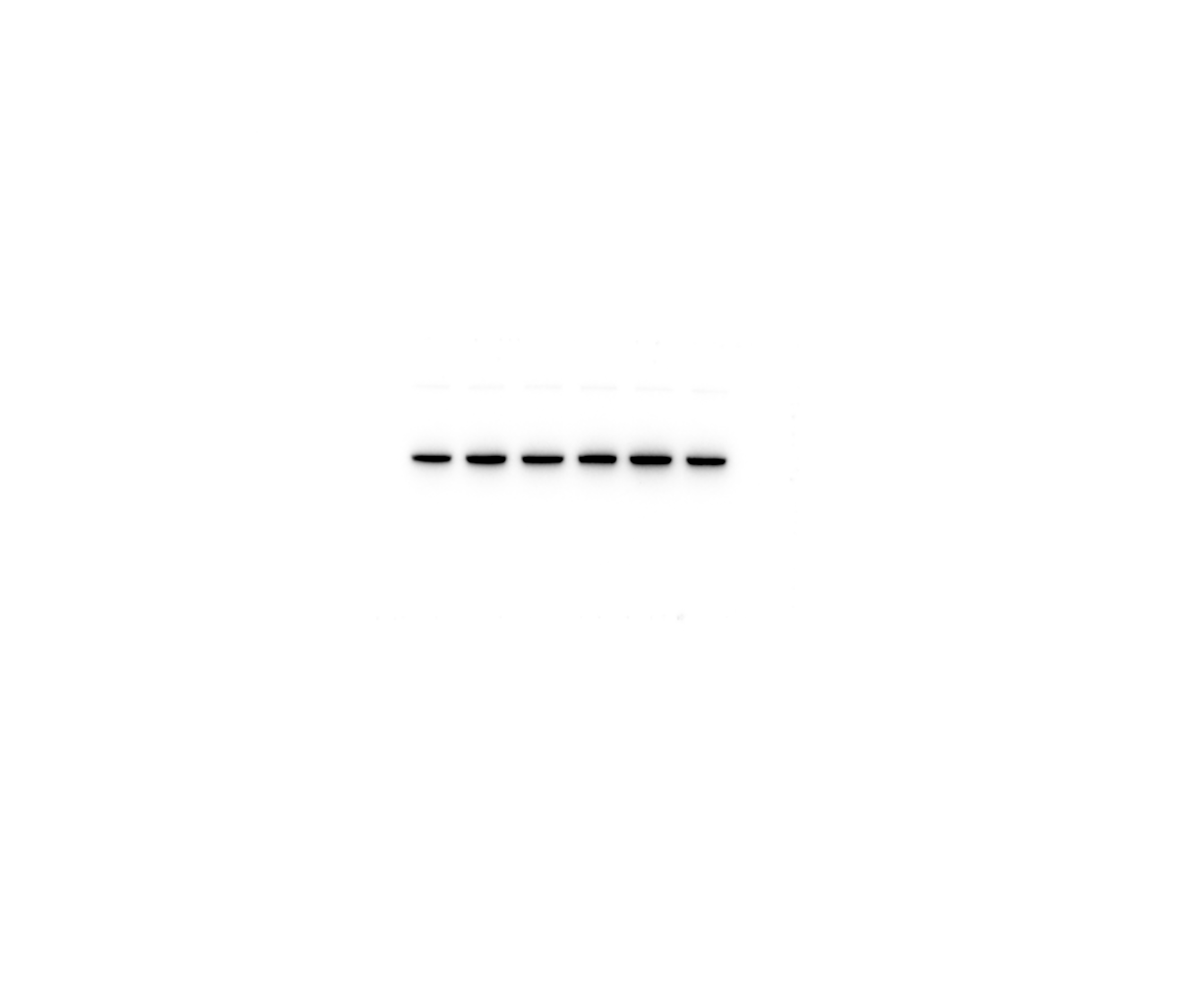

Supplement: Supplementary file 7 [file Data_Sheet_1.ZIP › BECLIN1,LC3/a┬-actin/3.Tif]

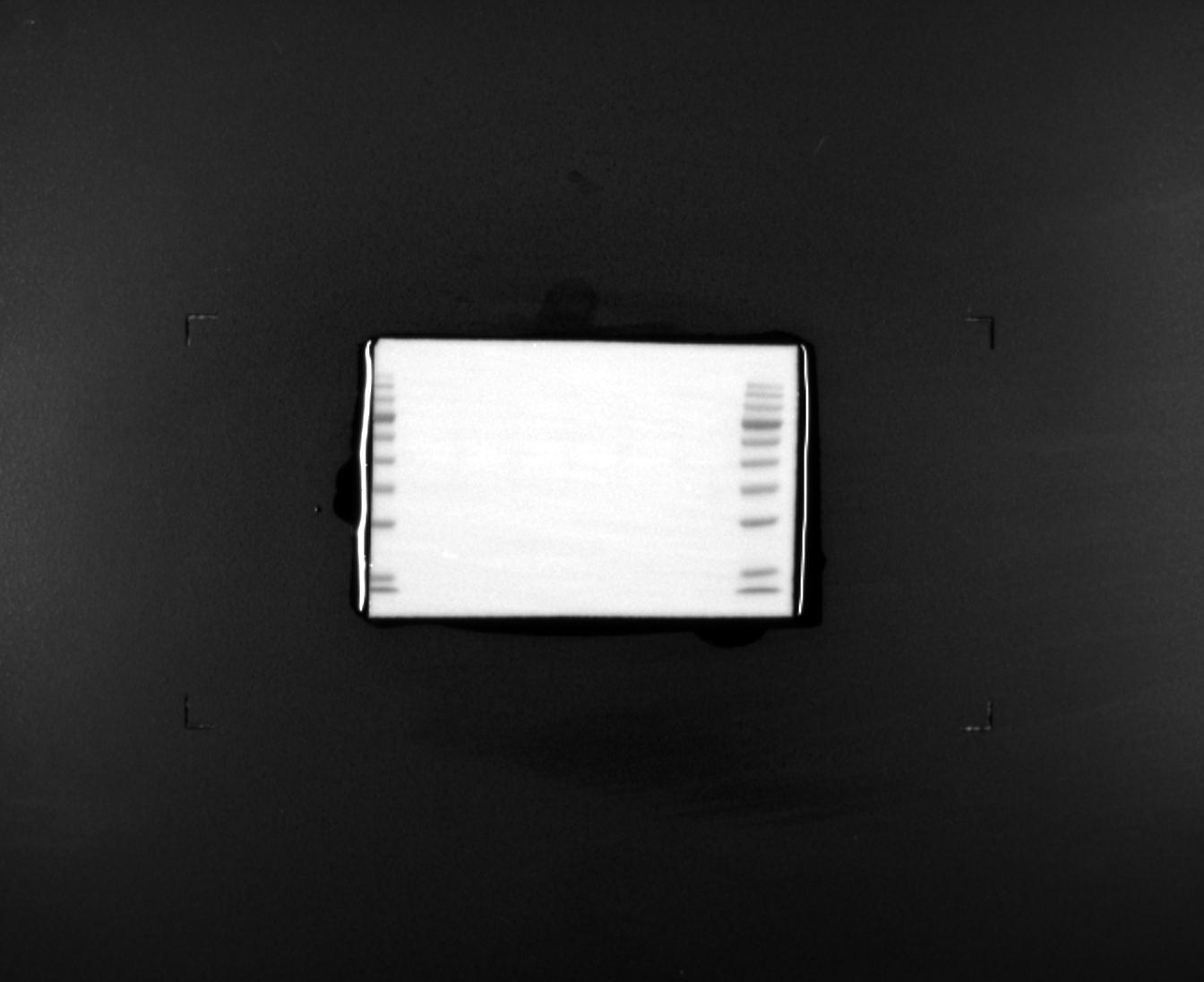

Supplement: Supplementary file 7 [file Data_Sheet_1.ZIP › BECLIN1,LC3/a┬-actin/3-t.Tif]

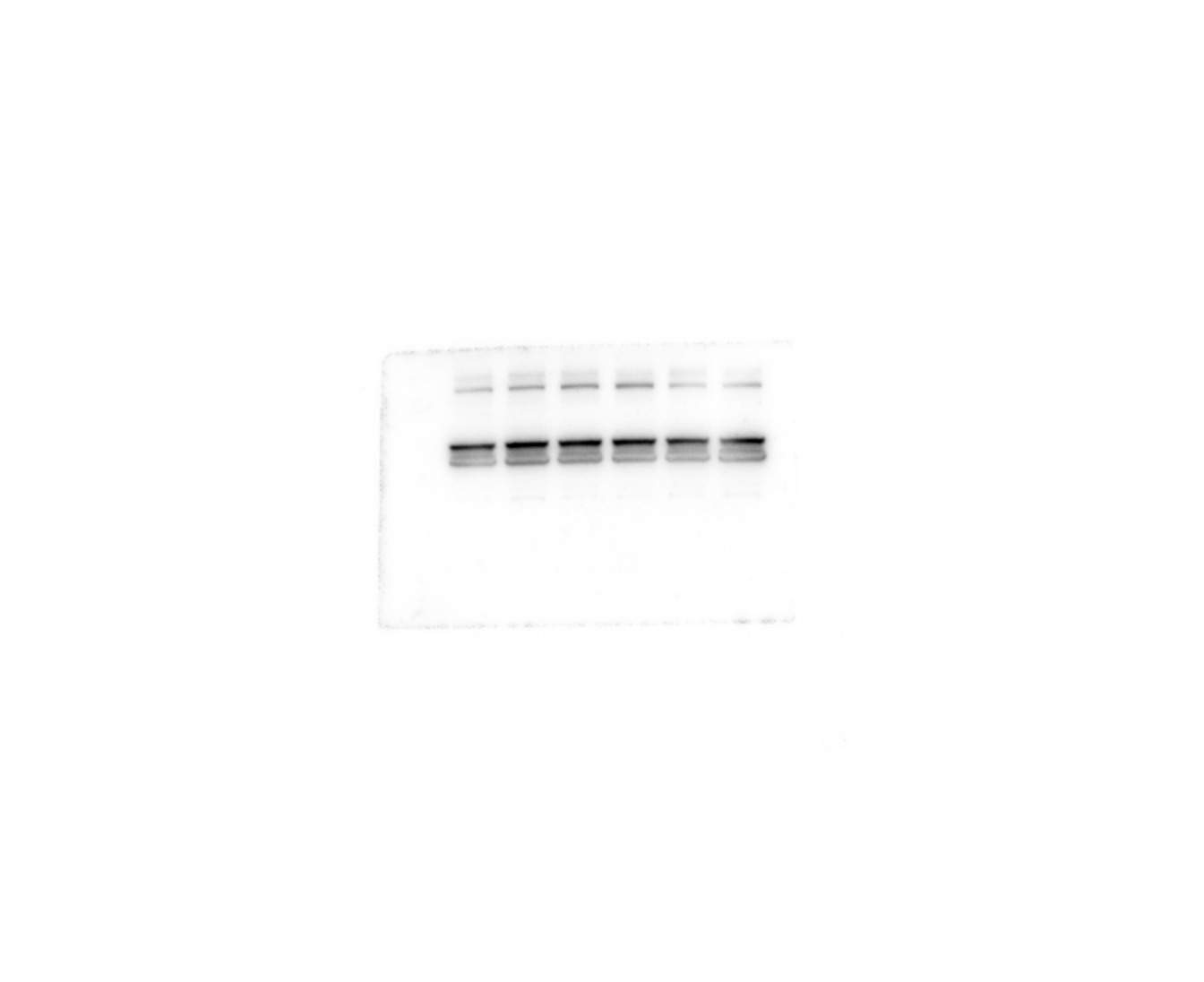

Supplement: Supplementary file 7 [file Data_Sheet_1.ZIP › PI3K-AKT-mTOR/akt/1.Tif]

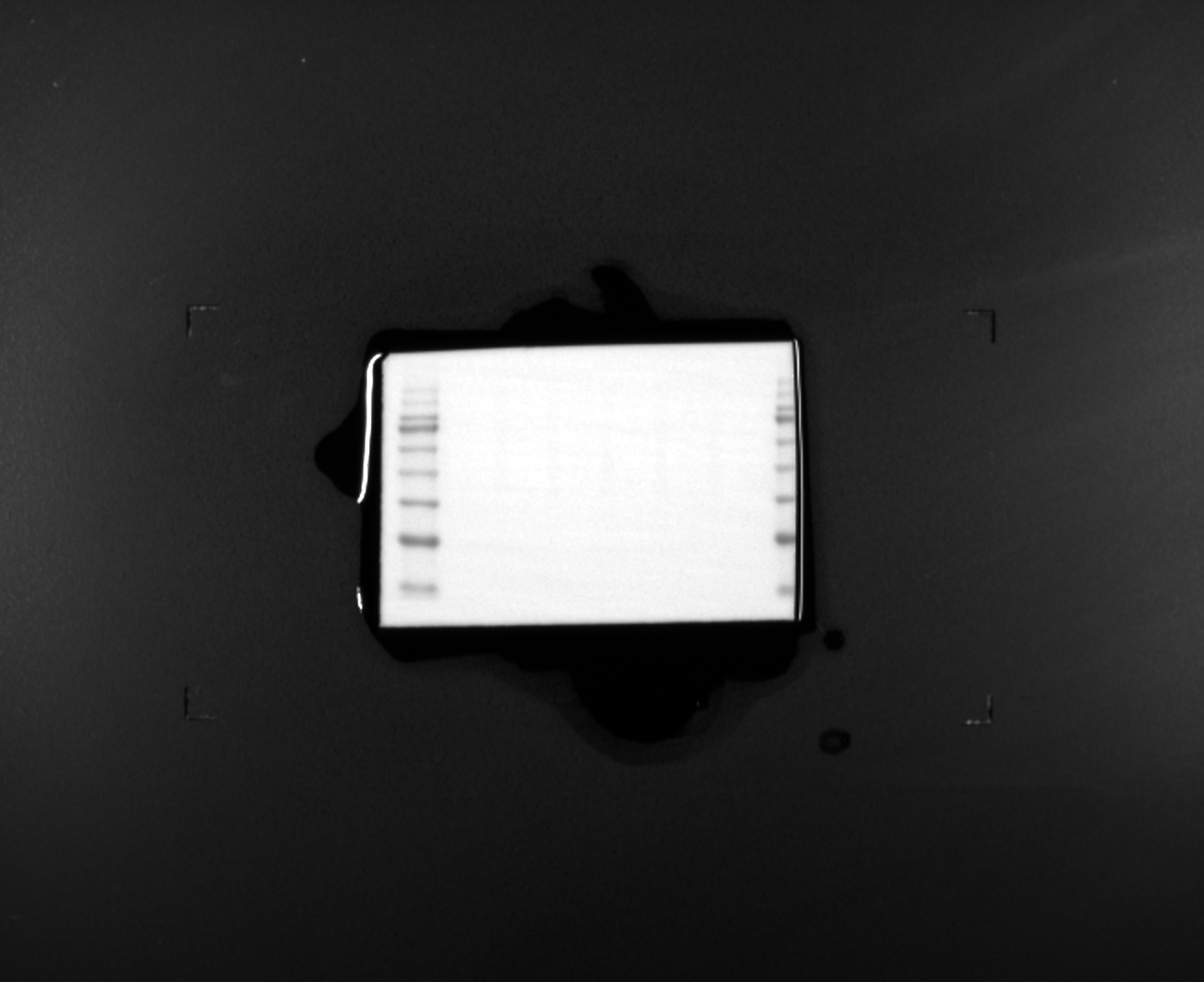

Supplement: Supplementary file 7 [file Data_Sheet_1.ZIP › PI3K-AKT-mTOR/akt/1-t.Tif]

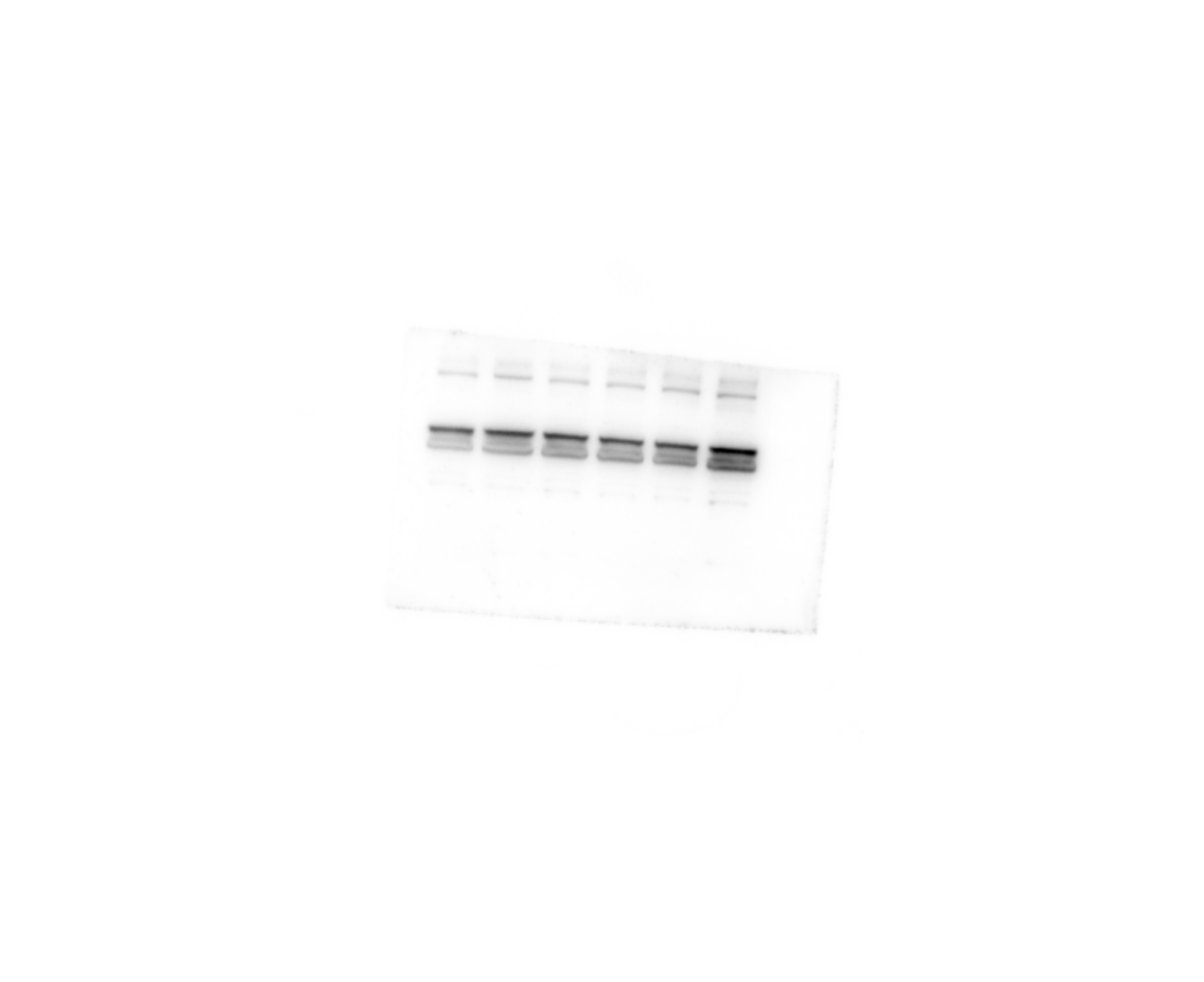

Supplement: Supplementary file 7 [file Data_Sheet_1.ZIP › PI3K-AKT-mTOR/akt/2.Tif]

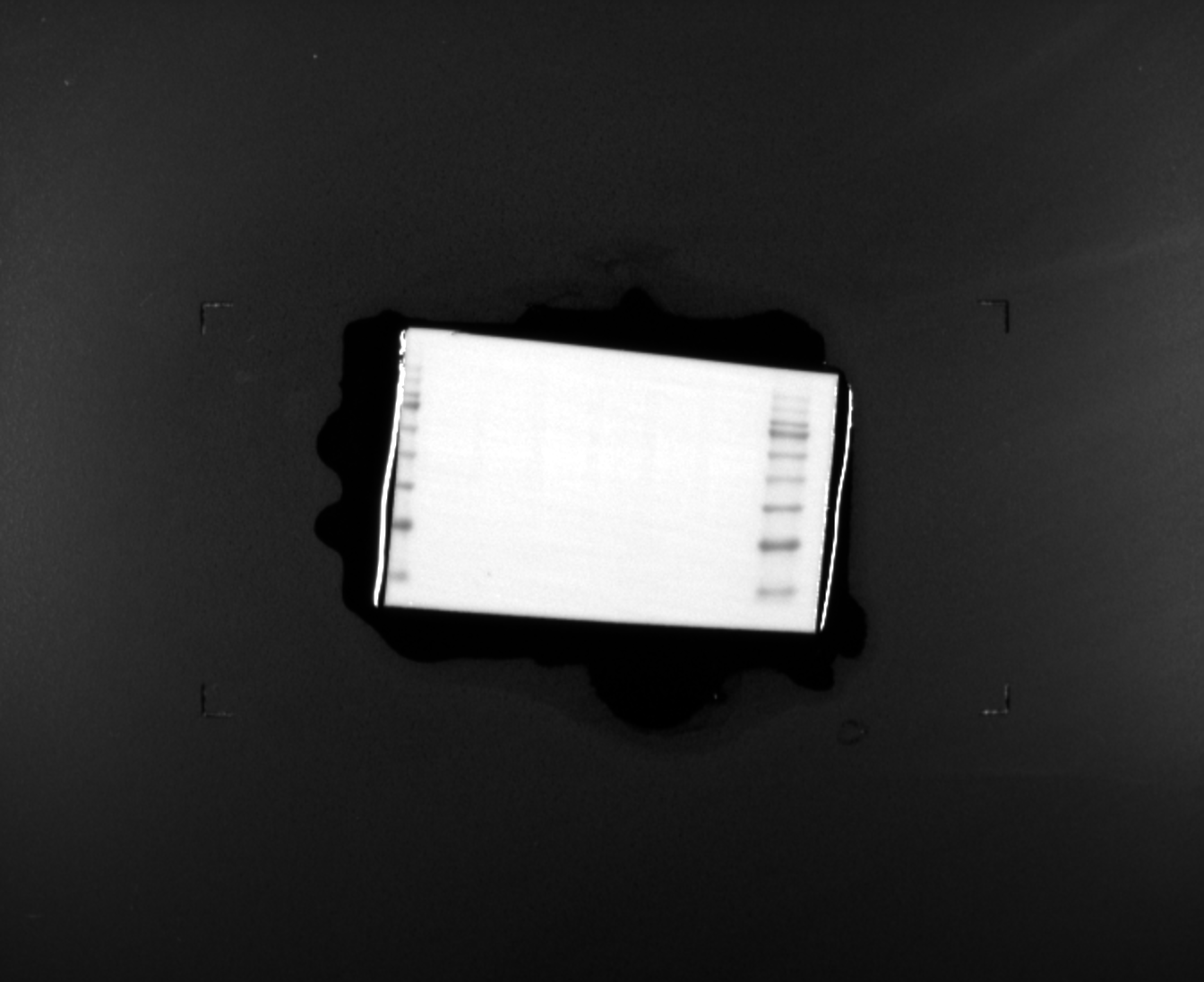

Supplement: Supplementary file 7 [file Data_Sheet_1.ZIP › PI3K-AKT-mTOR/akt/2-t.Tif]

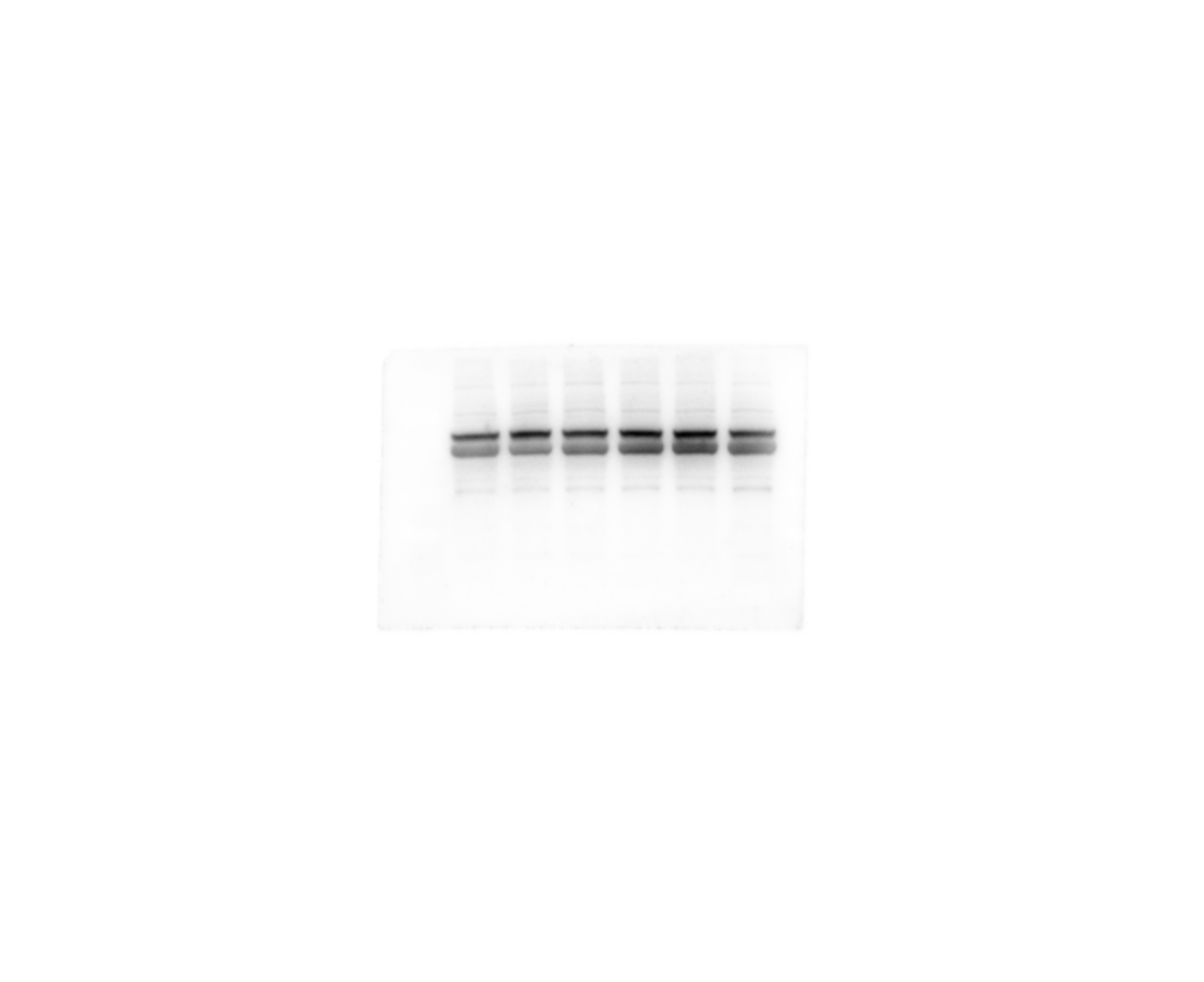

Supplement: Supplementary file 7 [file Data_Sheet_1.ZIP › PI3K-AKT-mTOR/akt/3.Tif]

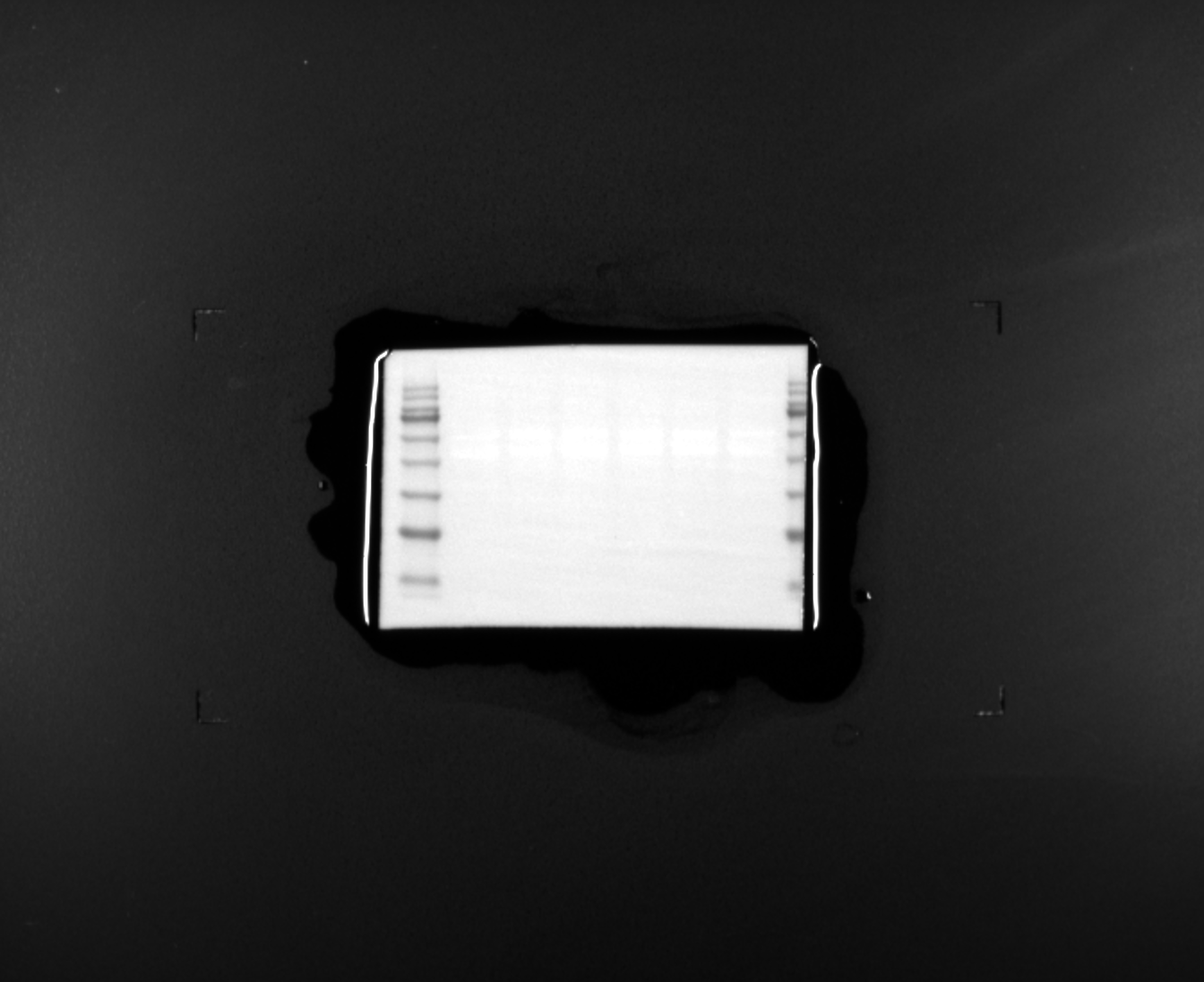

Supplement: Supplementary file 7 [file Data_Sheet_1.ZIP › PI3K-AKT-mTOR/akt/3-t.Tif]

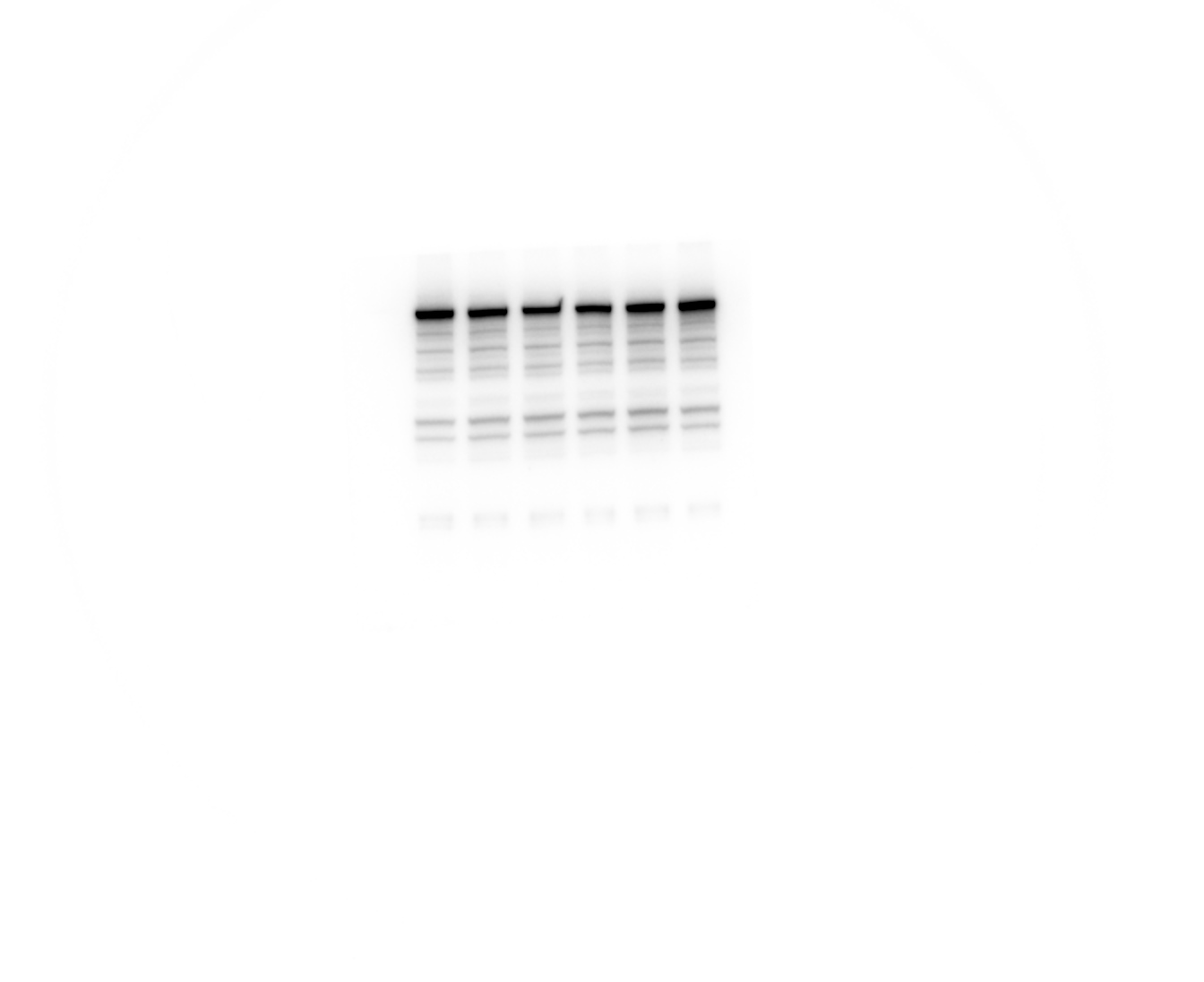

Supplement: Supplementary file 7 [file Data_Sheet_1.ZIP › PI3K-AKT-mTOR/mtor/1.Tif]

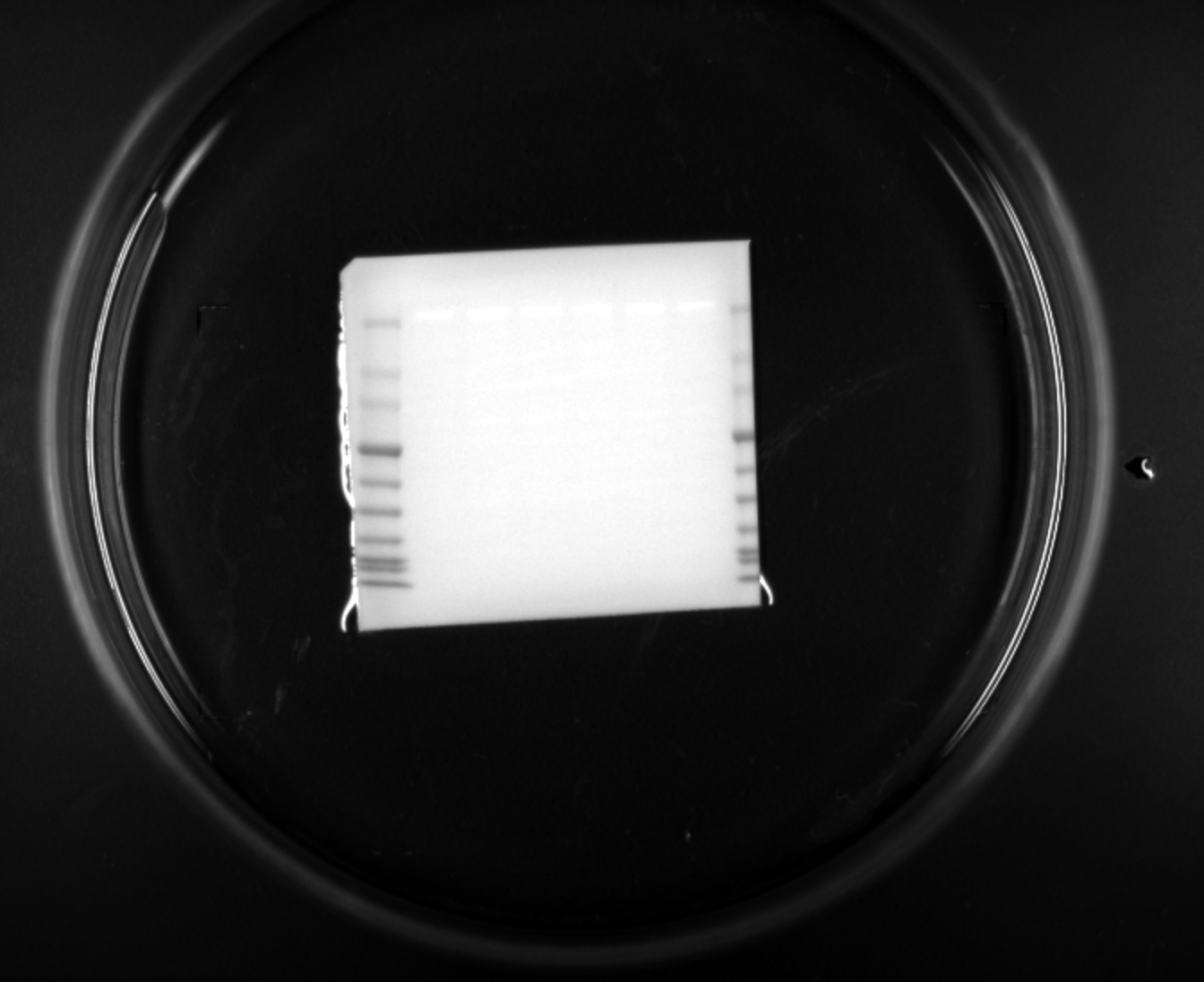

Supplement: Supplementary file 7 [file Data_Sheet_1.ZIP › PI3K-AKT-mTOR/mtor/1-t.Tif]

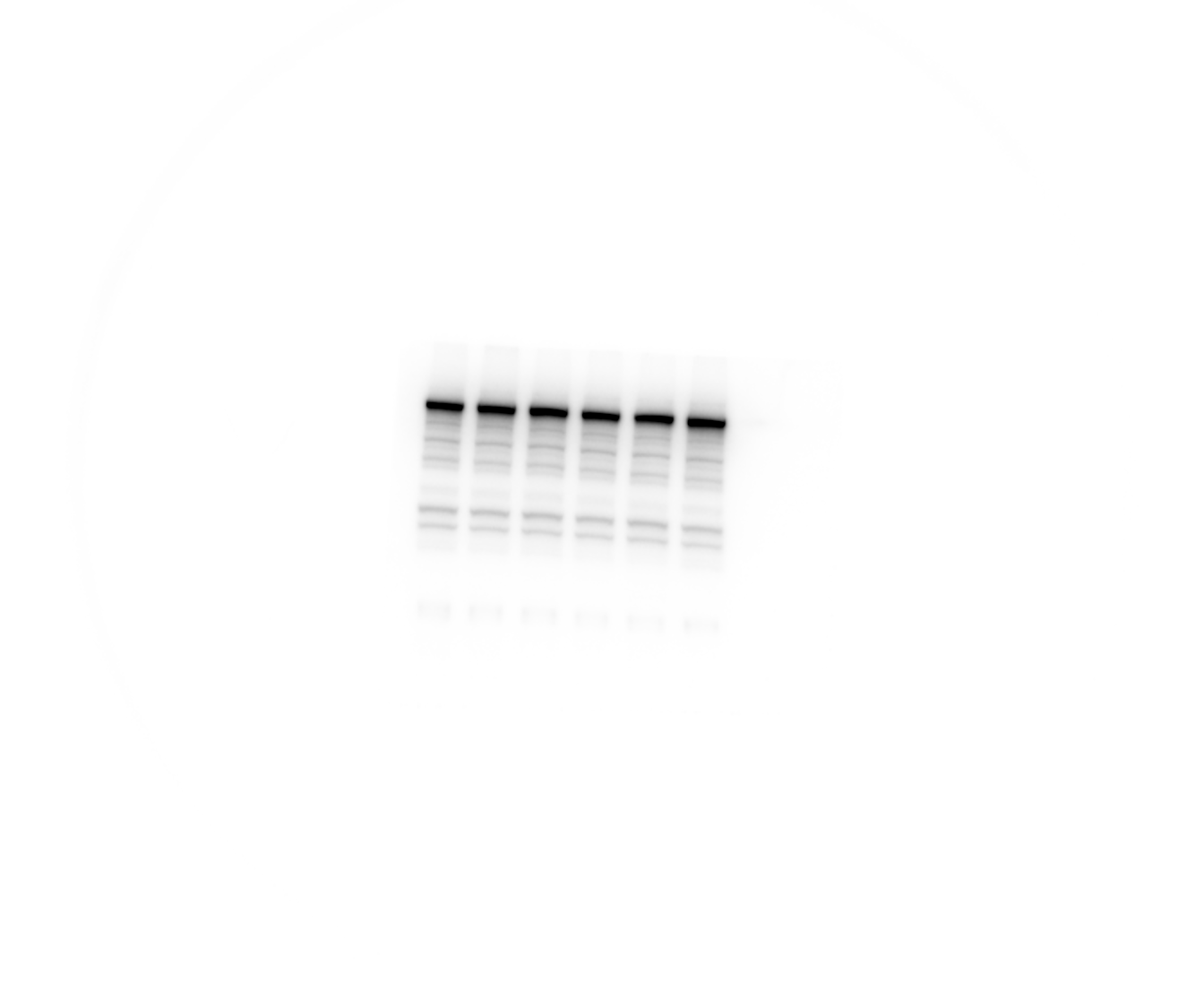

Supplement: Supplementary file 7 [file Data_Sheet_1.ZIP › PI3K-AKT-mTOR/mtor/2.Tif]

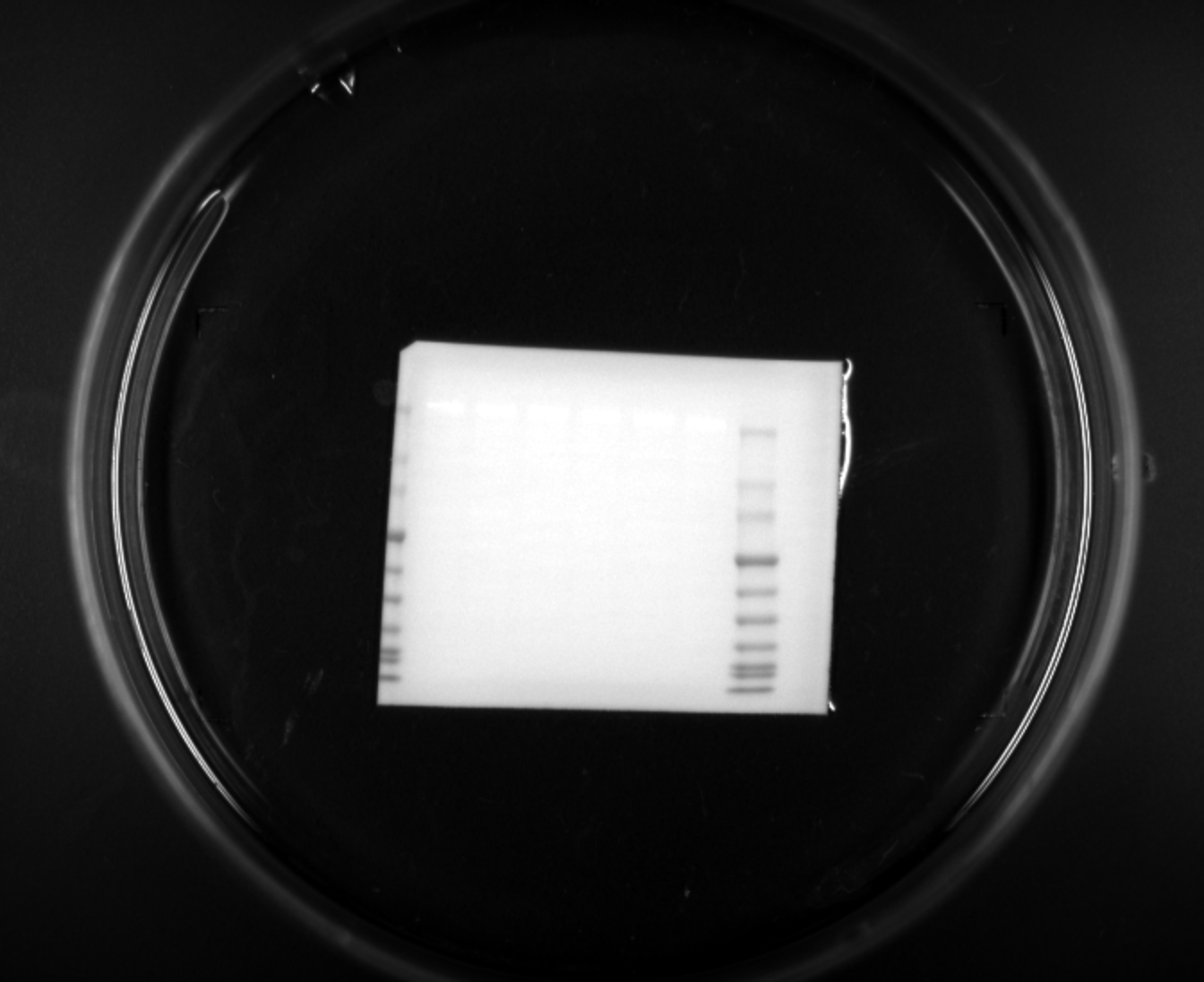

Supplement: Supplementary file 7 [file Data_Sheet_1.ZIP › PI3K-AKT-mTOR/mtor/2-t.Tif]

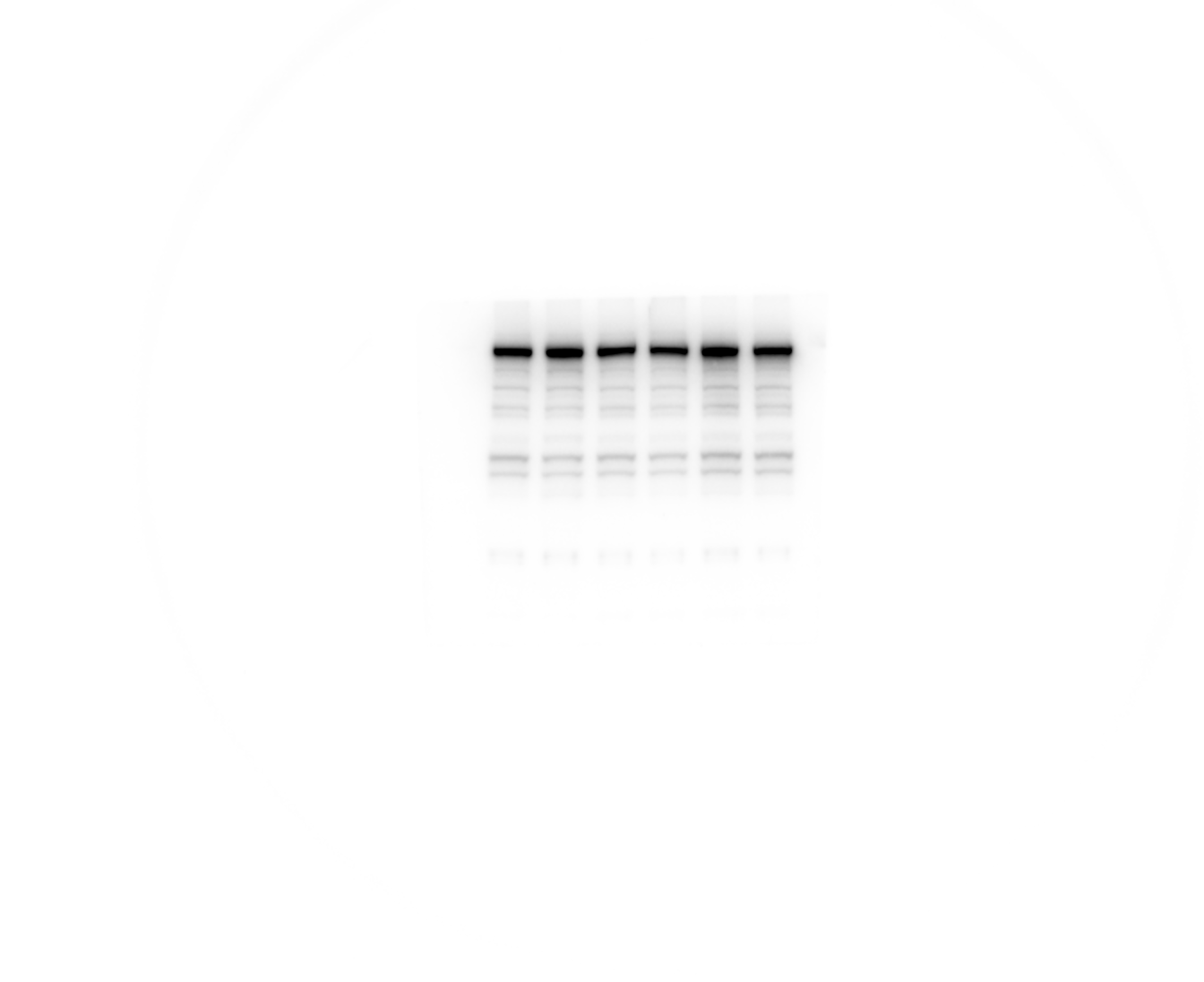

Supplement: Supplementary file 7 [file Data_Sheet_1.ZIP › PI3K-AKT-mTOR/mtor/3.Tif]

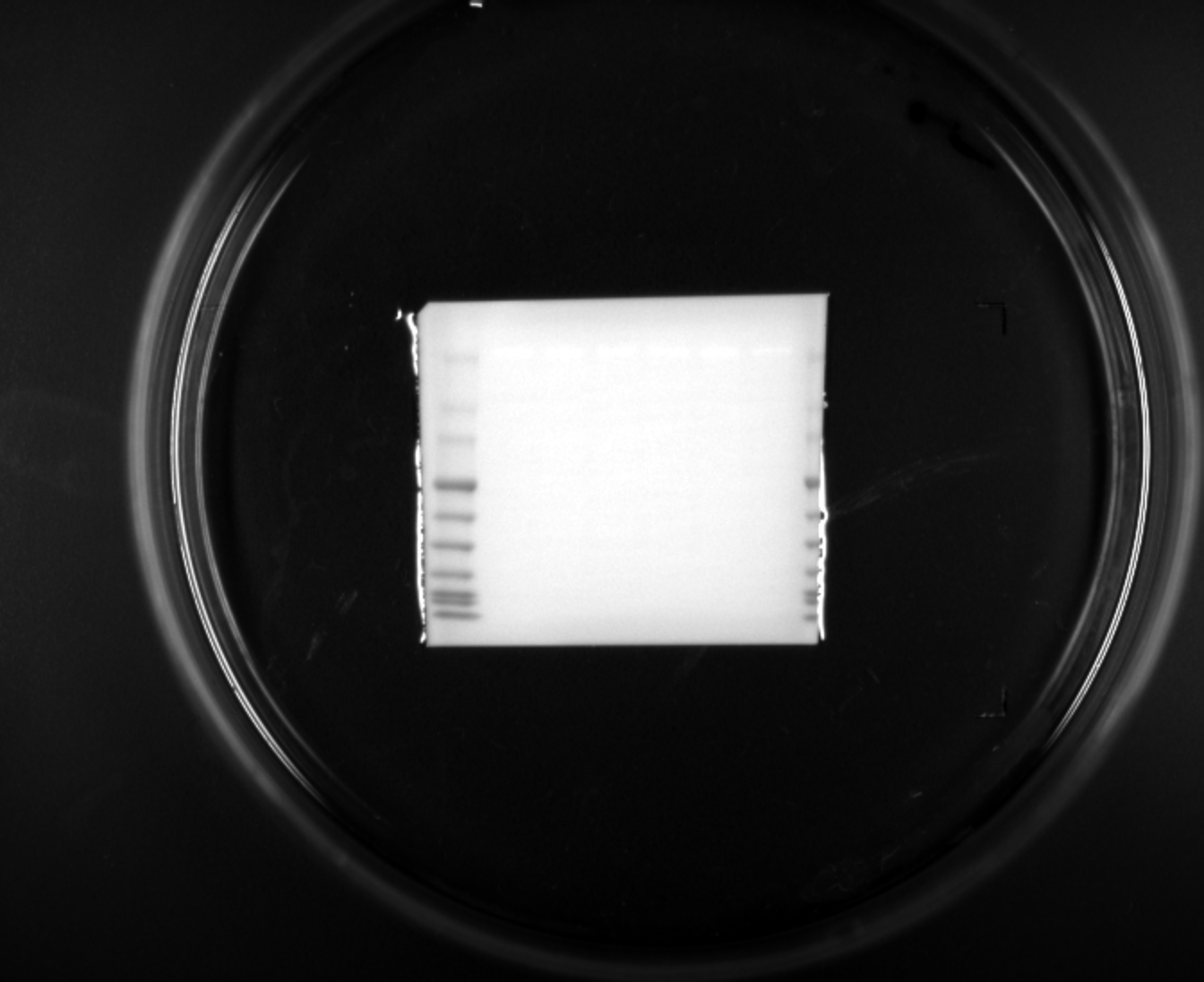

Supplement: Supplementary file 7 [file Data_Sheet_1.ZIP › PI3K-AKT-mTOR/mtor/3-t.Tif]

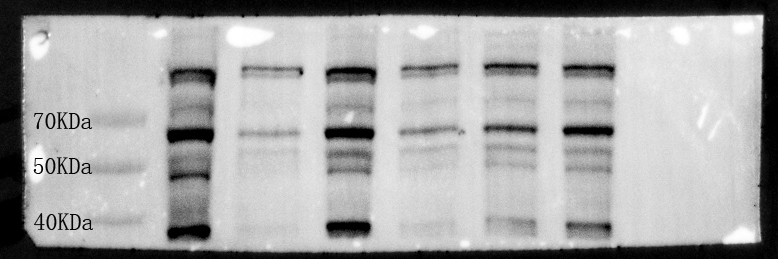

Supplement: Supplementary file 7 [file Data_Sheet_1.ZIP › PI3K-AKT-mTOR/P-AKT/P-AKT-1-╘¡═╝(┤°▒Ω╫ó).jpg]

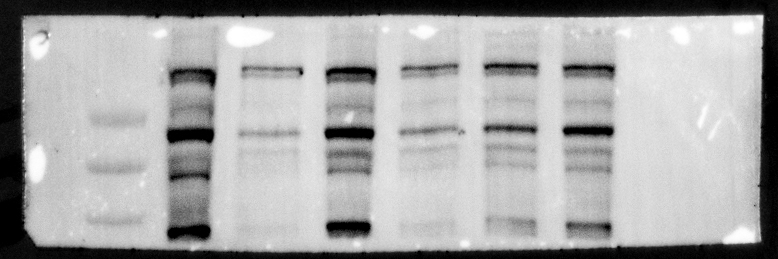

Supplement: Supplementary file 7 [file Data_Sheet_1.ZIP › PI3K-AKT-mTOR/P-AKT/P-AKT-1-╘¡═╝.jpg]

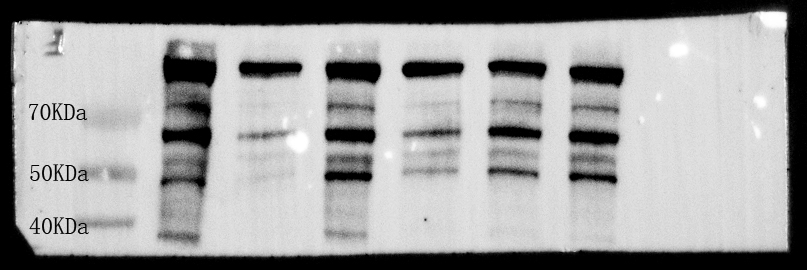

Supplement: Supplementary file 7 [file Data_Sheet_1.ZIP › PI3K-AKT-mTOR/P-AKT/P-AKT-2-╘¡═╝(┤°▒Ω╫ó).jpg]

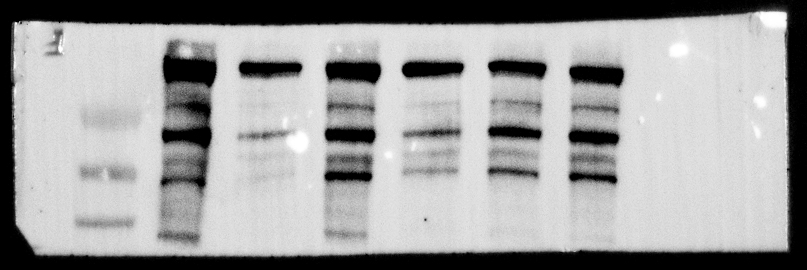

Supplement: Supplementary file 7 [file Data_Sheet_1.ZIP › PI3K-AKT-mTOR/P-AKT/P-AKT-2-╘¡═╝.jpg]

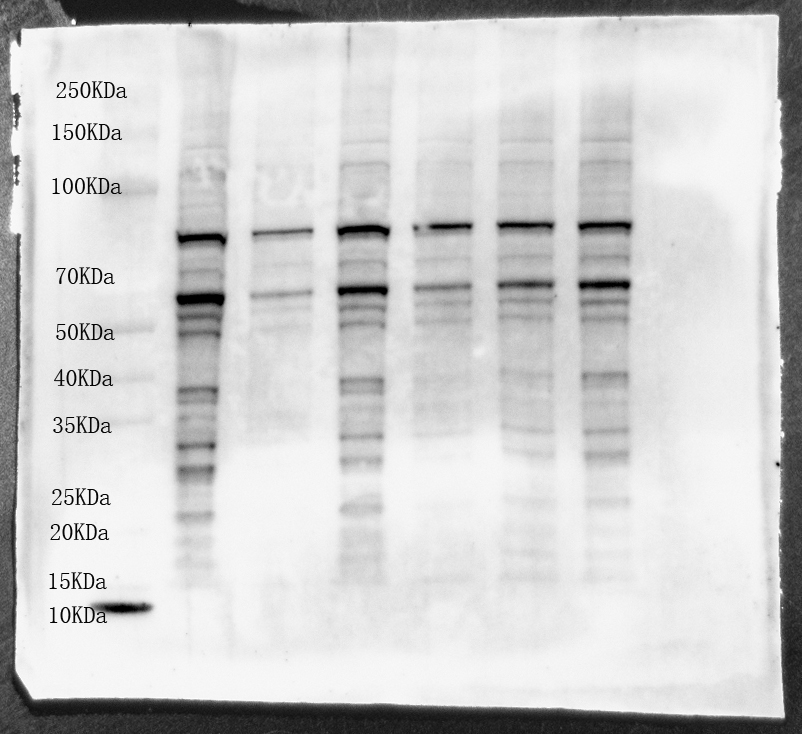

Supplement: Supplementary file 7 [file Data_Sheet_1.ZIP › PI3K-AKT-mTOR/P-AKT/P-AKT-3-╘¡═╝(┤°▒Ω╫ó).jpg]

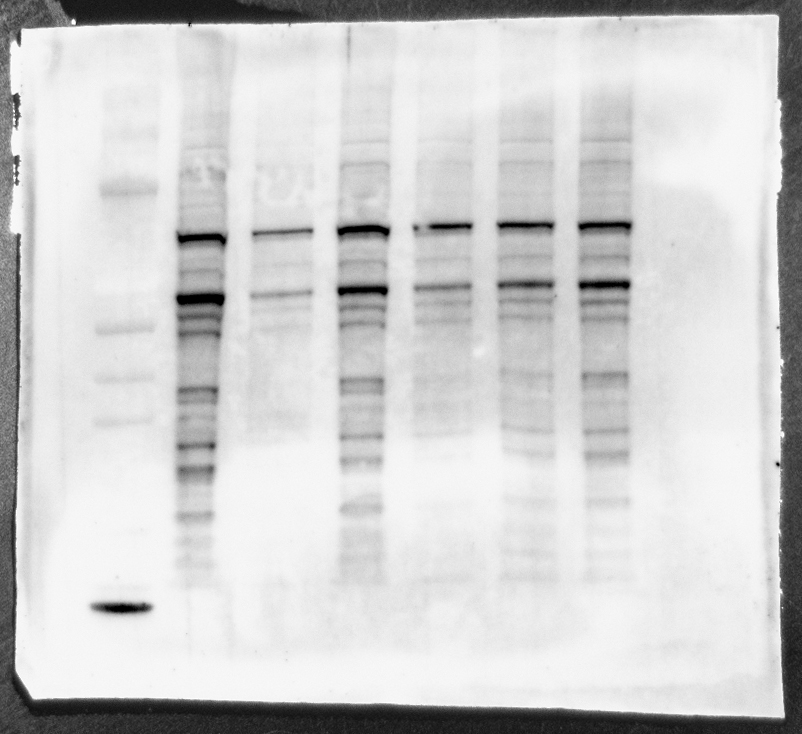

Supplement: Supplementary file 7 [file Data_Sheet_1.ZIP › PI3K-AKT-mTOR/P-AKT/P-AKT-3-╘¡═╝.jpg]

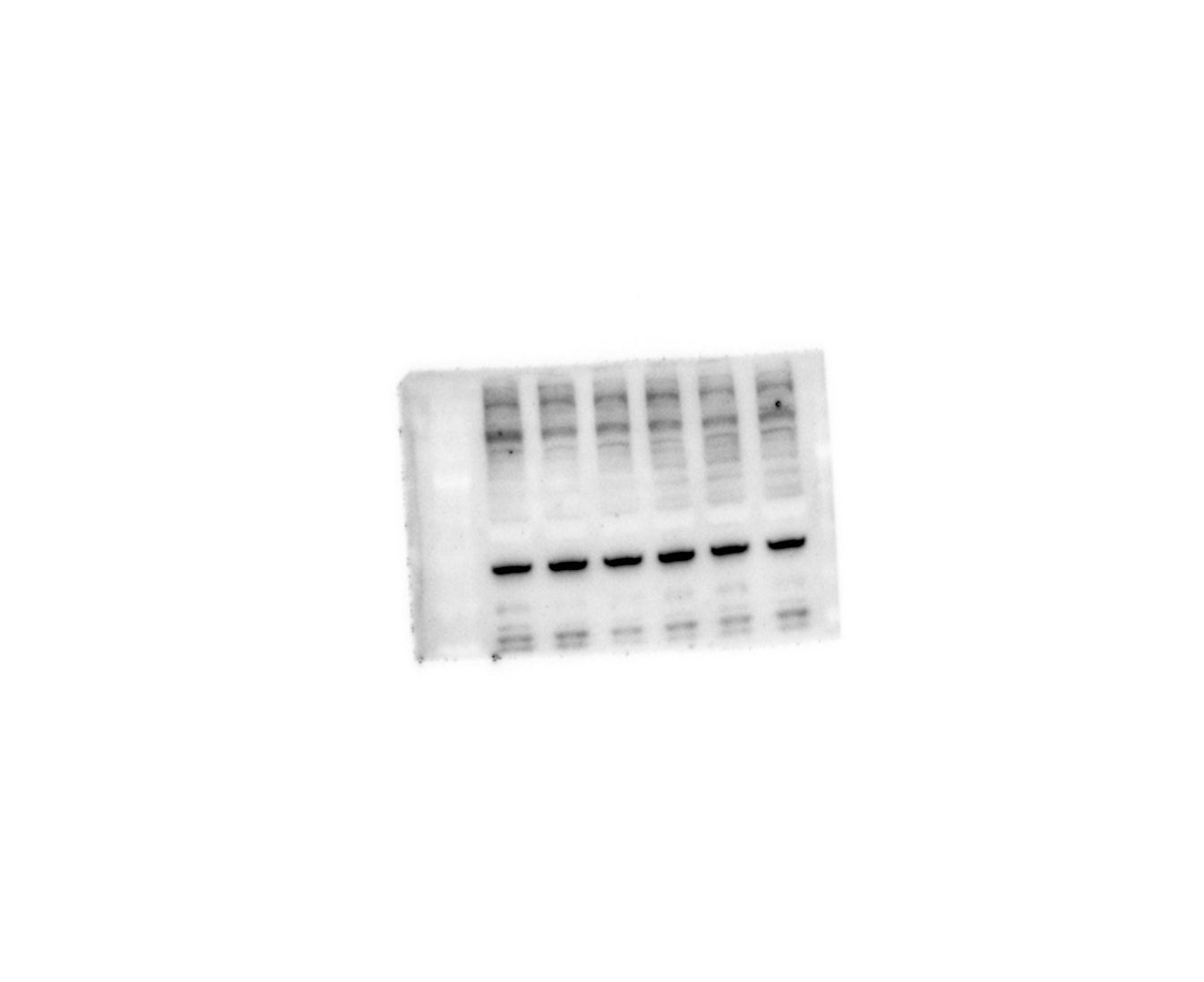

Supplement: Supplementary file 7 [file Data_Sheet_1.ZIP › PI3K-AKT-mTOR/PI3K/1.Tif]

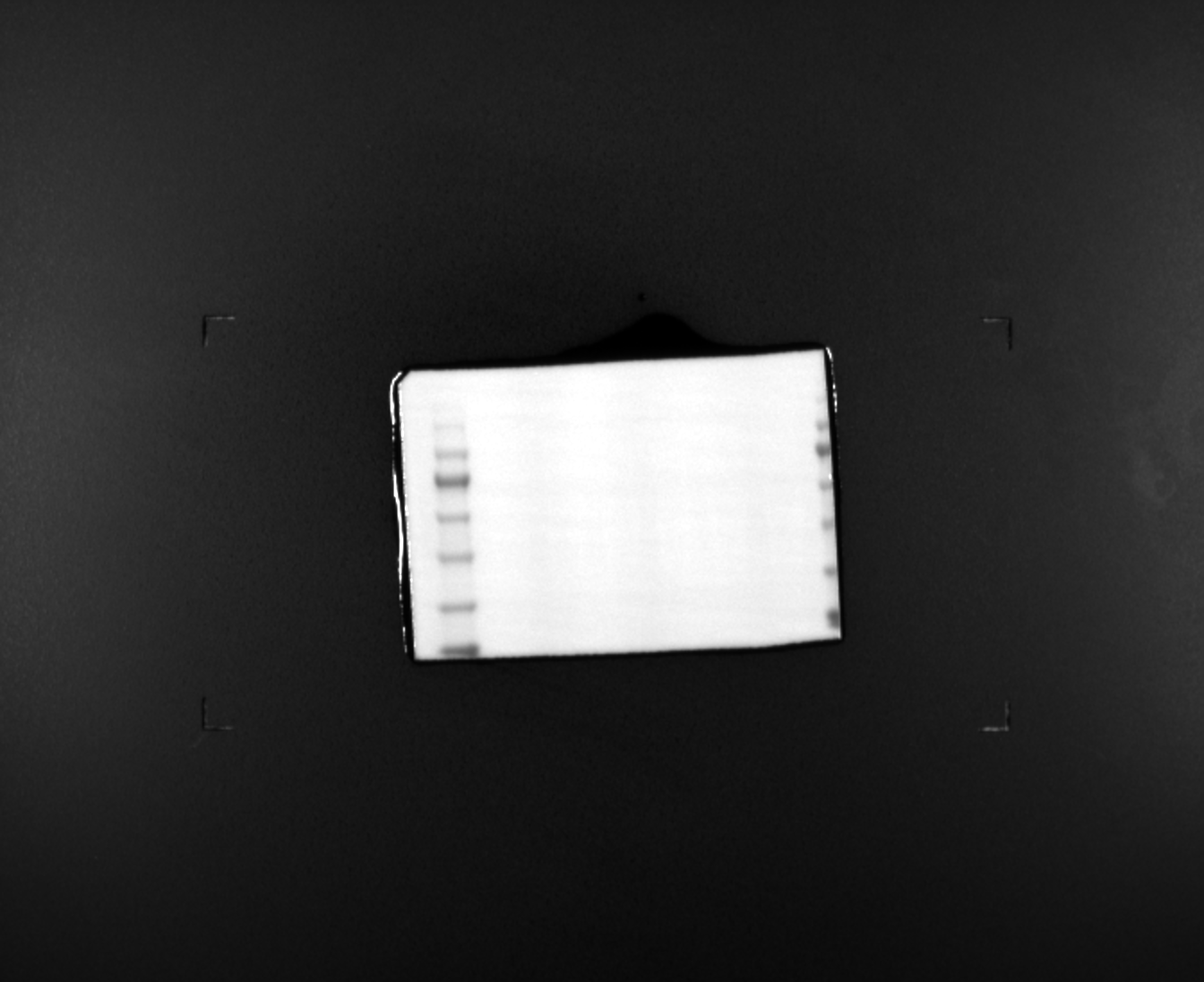

Supplement: Supplementary file 7 [file Data_Sheet_1.ZIP › PI3K-AKT-mTOR/PI3K/1-t.Tif]

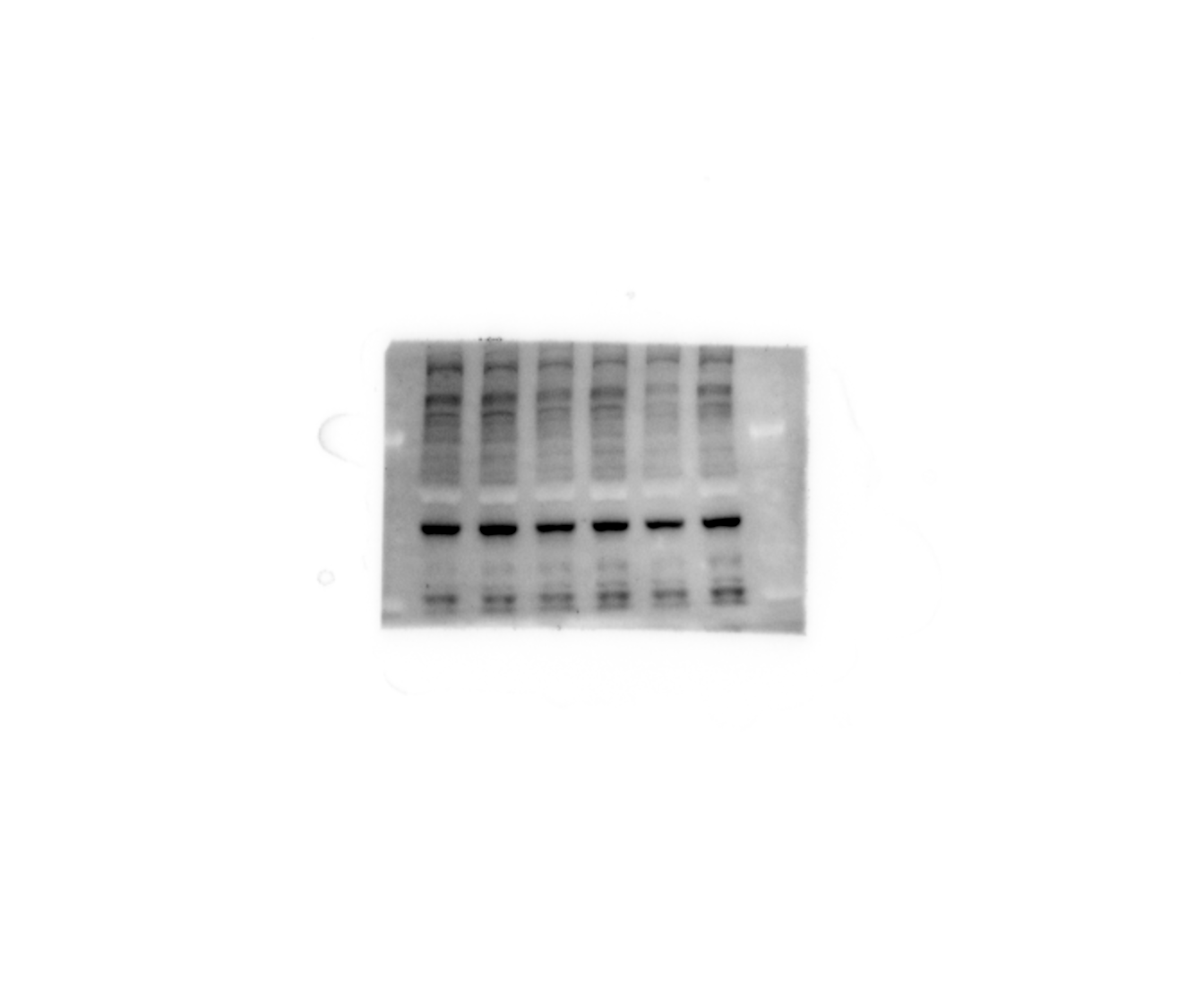

Supplement: Supplementary file 7 [file Data_Sheet_1.ZIP › PI3K-AKT-mTOR/PI3K/2.Tif]

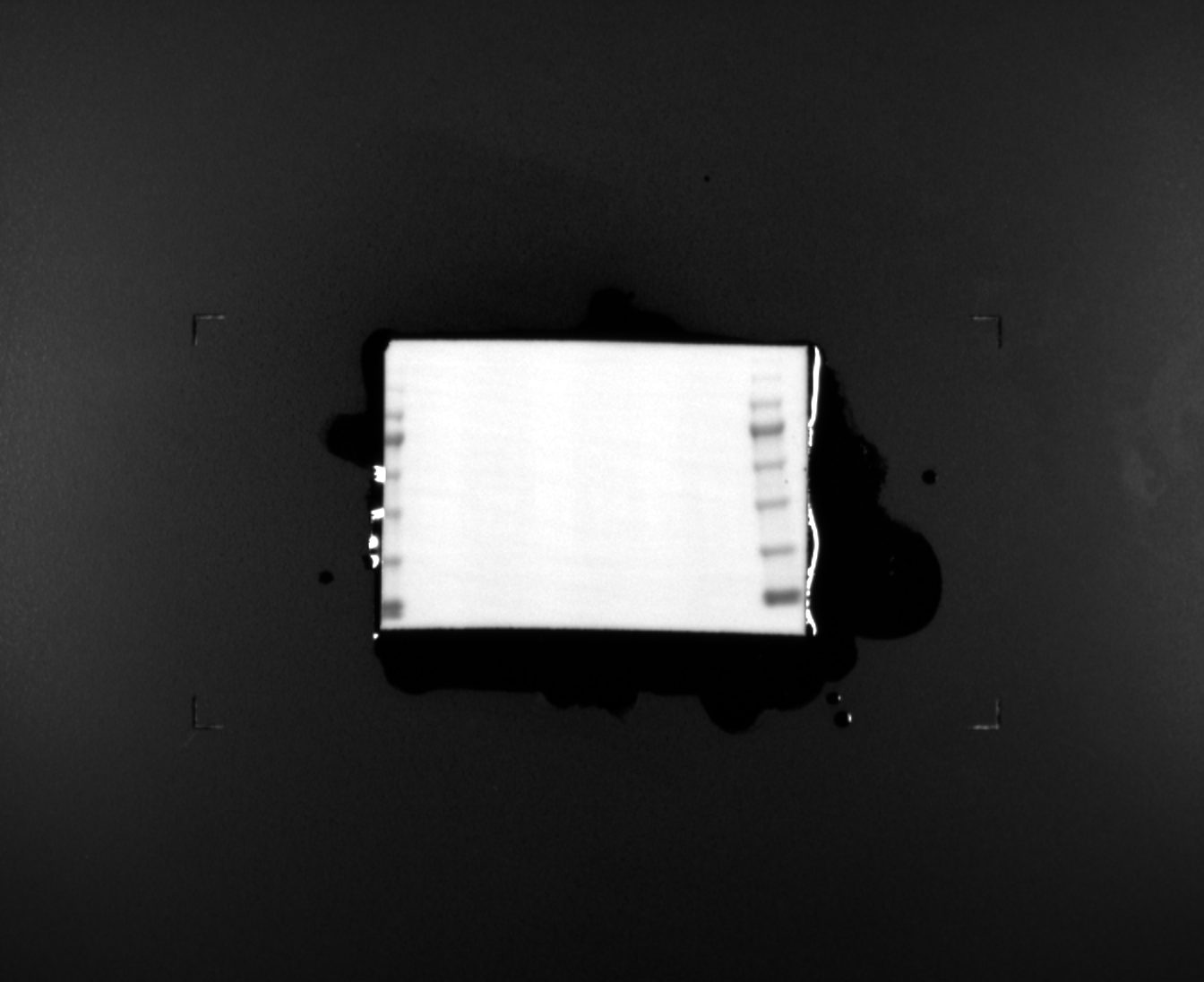

Supplement: Supplementary file 7 [file Data_Sheet_1.ZIP › PI3K-AKT-mTOR/PI3K/2-t.Tif]

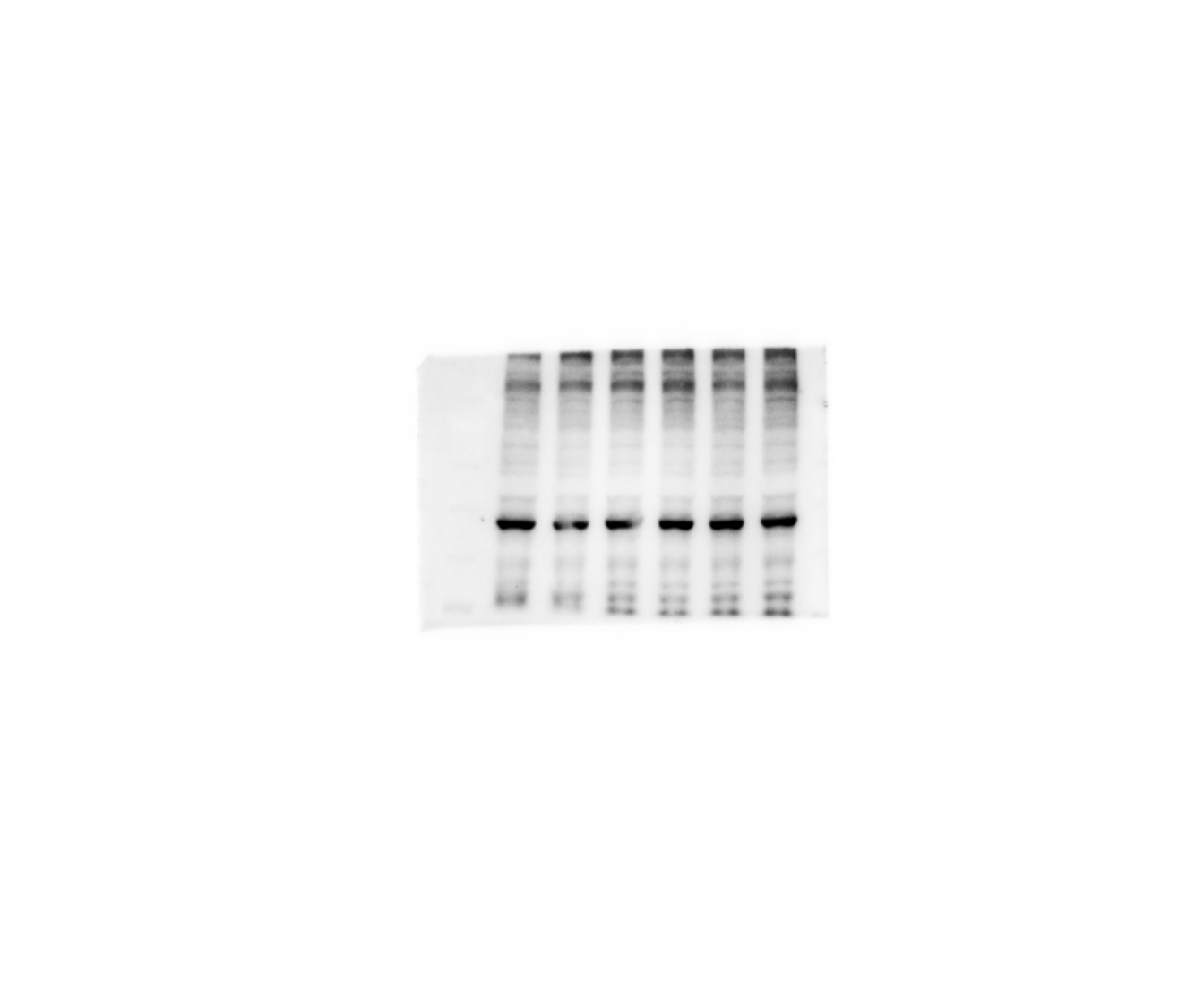

Supplement: Supplementary file 7 [file Data_Sheet_1.ZIP › PI3K-AKT-mTOR/PI3K/3.tif]

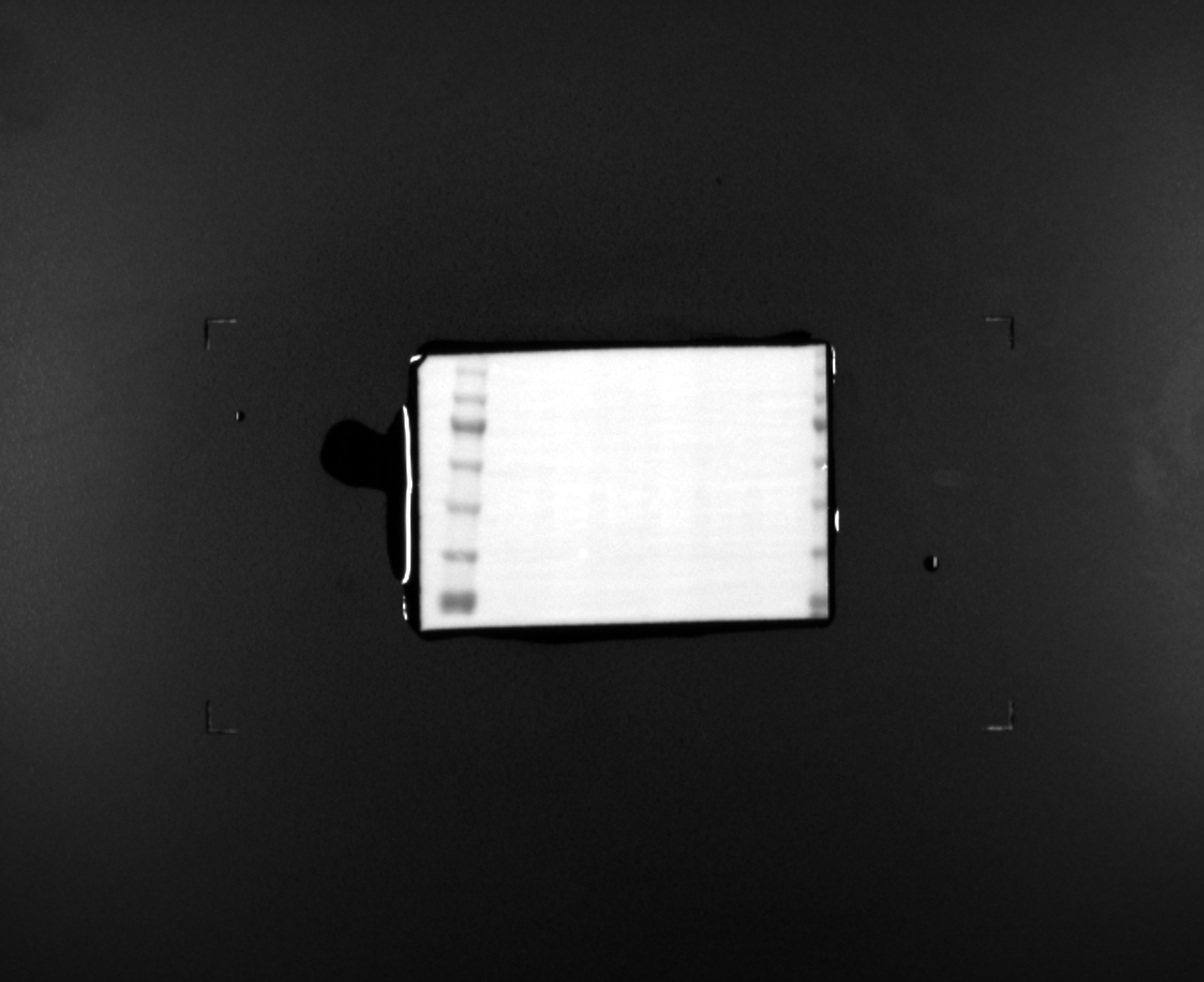

Supplement: Supplementary file 7 [file Data_Sheet_1.ZIP › PI3K-AKT-mTOR/PI3K/3-t.Tif]

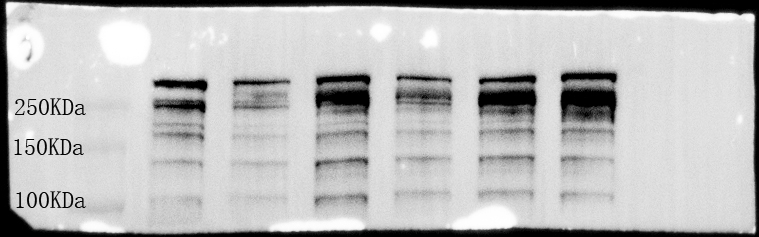

Supplement: Supplementary file 7 [file Data_Sheet_1.ZIP › PI3K-AKT-mTOR/P-MTOR/P-mtor-1-╘¡═╝ú¿┤°▒Ω╫óú⌐.jpg]

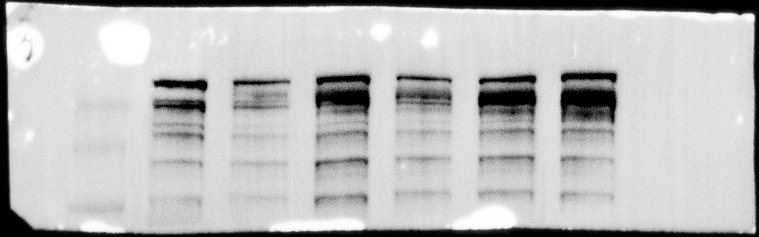

Supplement: Supplementary file 7 [file Data_Sheet_1.ZIP › PI3K-AKT-mTOR/P-MTOR/P-mtor-1-╘¡═╝.jpg]

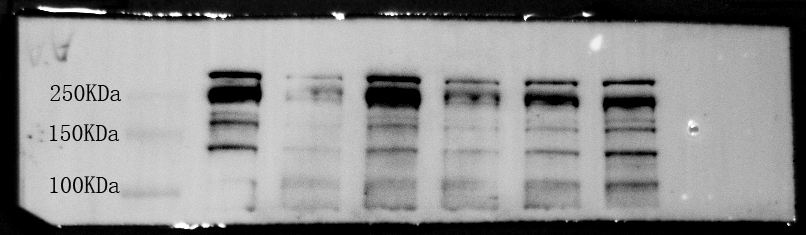

Supplement: Supplementary file 7 [file Data_Sheet_1.ZIP › PI3K-AKT-mTOR/P-MTOR/P-mtor-2-╘¡═╝(┤°▒Ω╫ó).jpg]

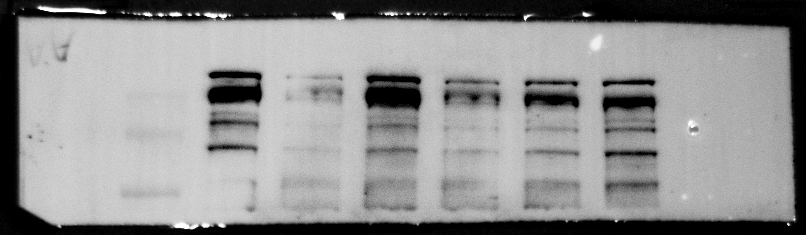

Supplement: Supplementary file 7 [file Data_Sheet_1.ZIP › PI3K-AKT-mTOR/P-MTOR/P-mtor-2-╘¡═╝.jpg]

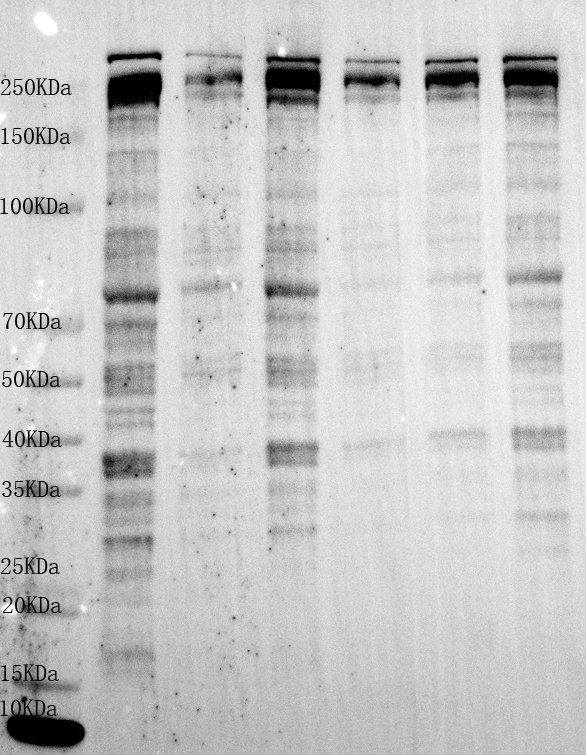

Supplement: Supplementary file 7 [file Data_Sheet_1.ZIP › PI3K-AKT-mTOR/P-MTOR/P-mtor-3-╘¡═╝(┤°▒Ω╫ó).jpg]

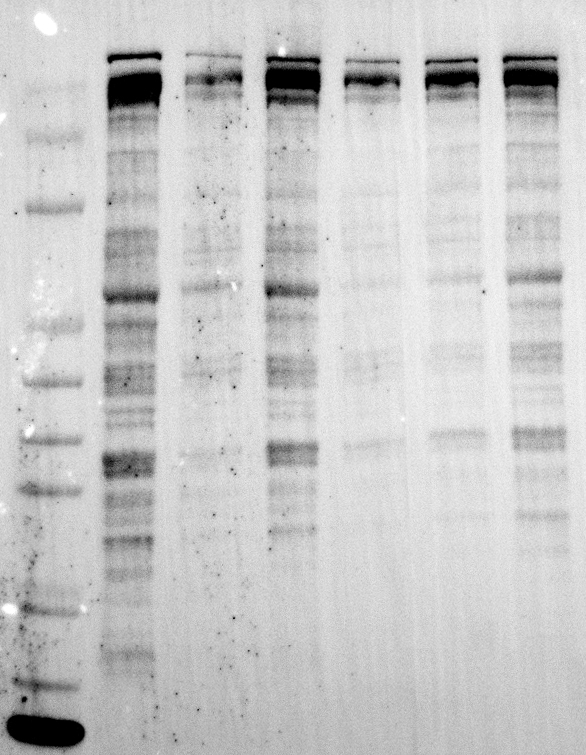

Supplement: Supplementary file 7 [file Data_Sheet_1.ZIP › PI3K-AKT-mTOR/P-MTOR/P-mtor-3-╘¡═╝.jpg]

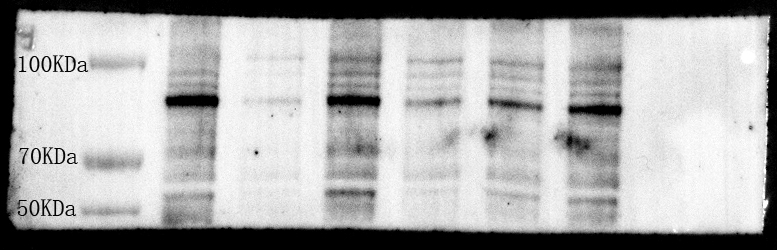

Supplement: Supplementary file 7 [file Data_Sheet_1.ZIP › PI3K-AKT-mTOR/P-PI3K/P-PI3K-1-╘¡═╝(┤°▒Ω╫ó).jpg]

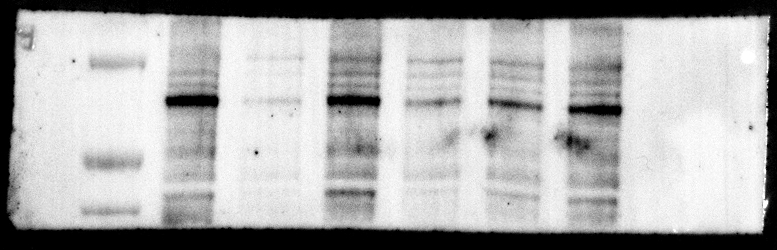

Supplement: Supplementary file 7 [file Data_Sheet_1.ZIP › PI3K-AKT-mTOR/P-PI3K/P-PI3K-1-╘¡═╝.jpg]

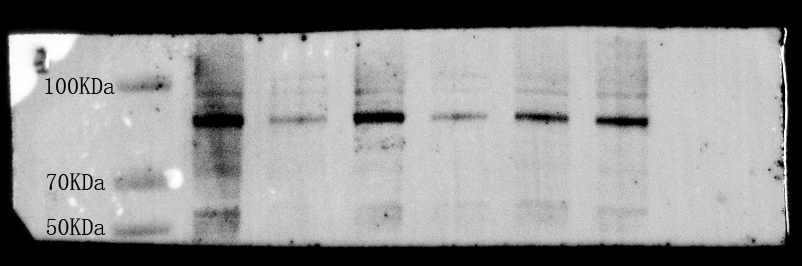

Supplement: Supplementary file 7 [file Data_Sheet_1.ZIP › PI3K-AKT-mTOR/P-PI3K/P-PI3K-2-╘¡═╝ú¿┤°▒Ω╫óú⌐.jpg]

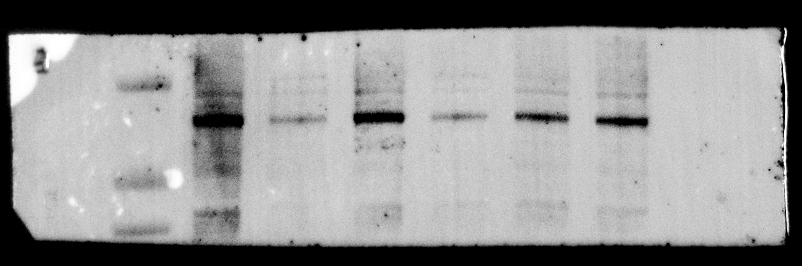

Supplement: Supplementary file 7 [file Data_Sheet_1.ZIP › PI3K-AKT-mTOR/P-PI3K/P-PI3K-2-╘¡═╝.jpg]

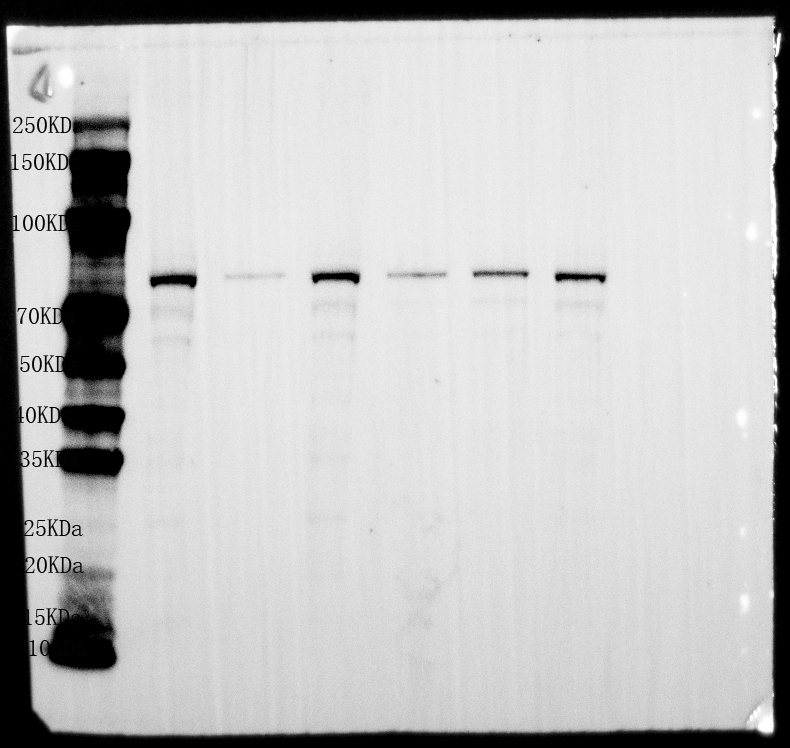

Supplement: Supplementary file 7 [file Data_Sheet_1.ZIP › PI3K-AKT-mTOR/P-PI3K/P-PI3K-3-╘¡═╝(┤°▒Ω╫ó).jpg]

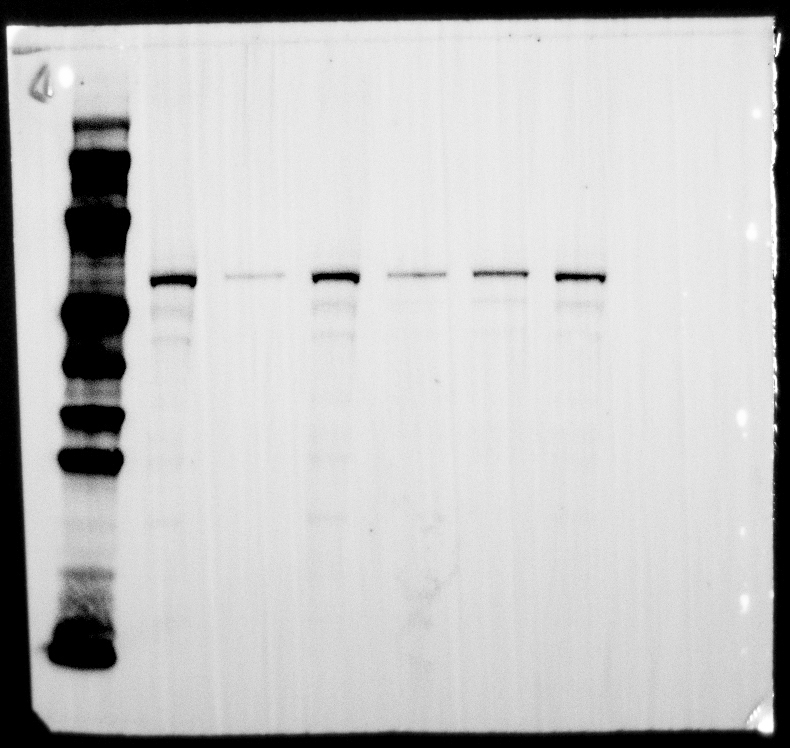

Supplement: Supplementary file 7 [file Data_Sheet_1.ZIP › PI3K-AKT-mTOR/P-PI3K/P-PI3K-3-╘¡═╝.jpg]

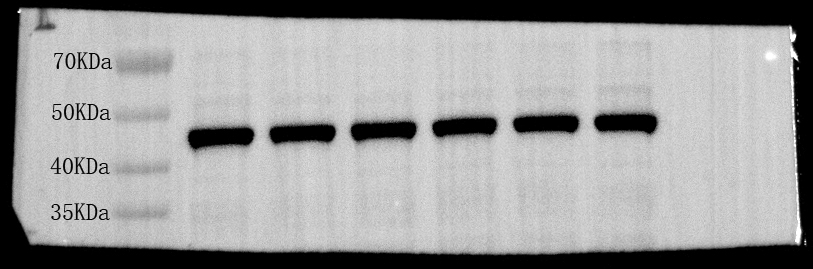

Supplement: Supplementary file 7 [file Data_Sheet_1.ZIP › PI3K-AKT-mTOR/P-a┬-actin/actin-1-╘¡═╝(┤°▒Ω╫ó).jpg]

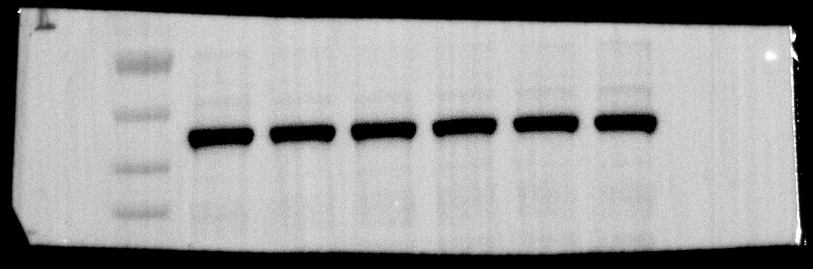

Supplement: Supplementary file 7 [file Data_Sheet_1.ZIP › PI3K-AKT-mTOR/P-a┬-actin/actin-1-╘¡═╝.jpg]

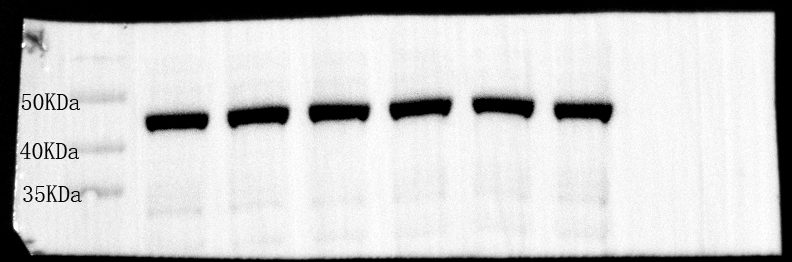

Supplement: Supplementary file 7 [file Data_Sheet_1.ZIP › PI3K-AKT-mTOR/P-a┬-actin/actin-2-╘¡═╝(┤°▒Ω╫ó).jpg]

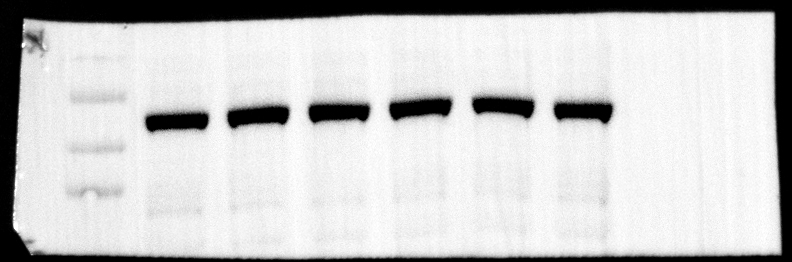

Supplement: Supplementary file 7 [file Data_Sheet_1.ZIP › PI3K-AKT-mTOR/P-a┬-actin/actin-2-╘¡═╝.jpg]

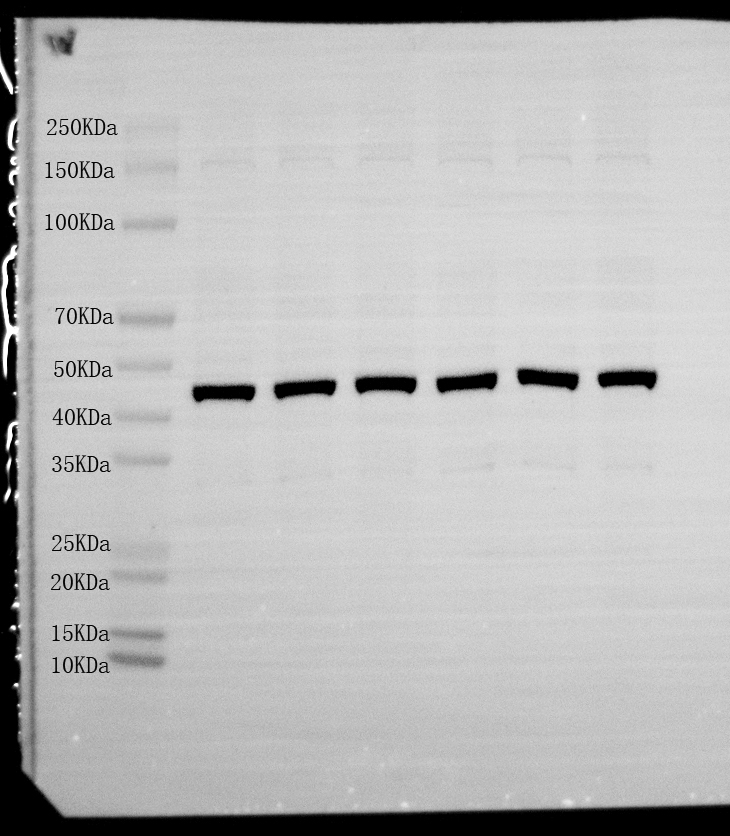

Supplement: Supplementary file 7 [file Data_Sheet_1.ZIP › PI3K-AKT-mTOR/P-a┬-actin/actin-3-╘¡═╝(┤°▒Ω╫ó).jpg]

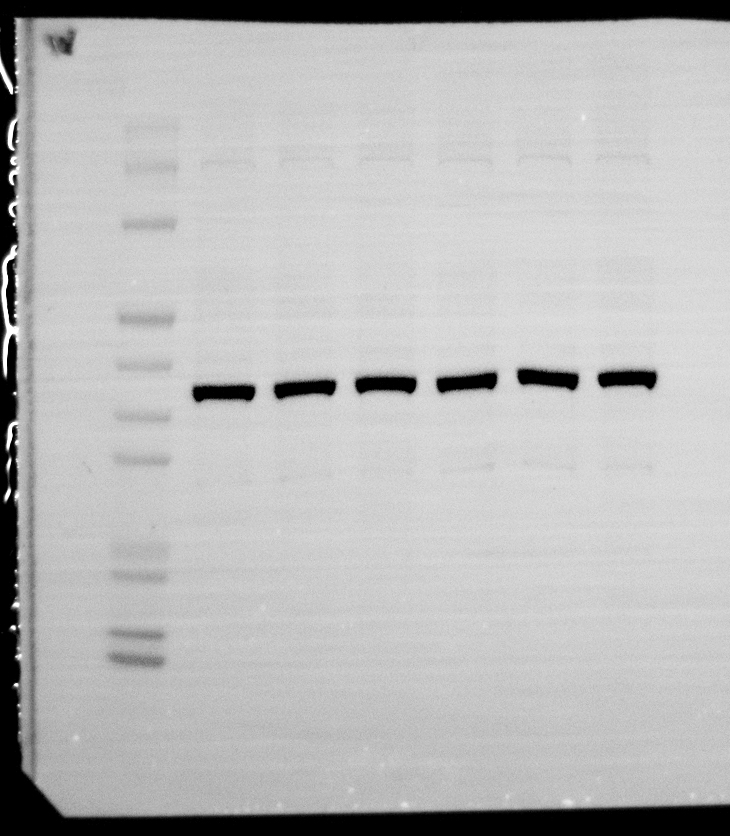

Supplement: Supplementary file 7 [file Data_Sheet_1.ZIP › PI3K-AKT-mTOR/P-a┬-actin/actin-3-╘¡═╝.jpg]

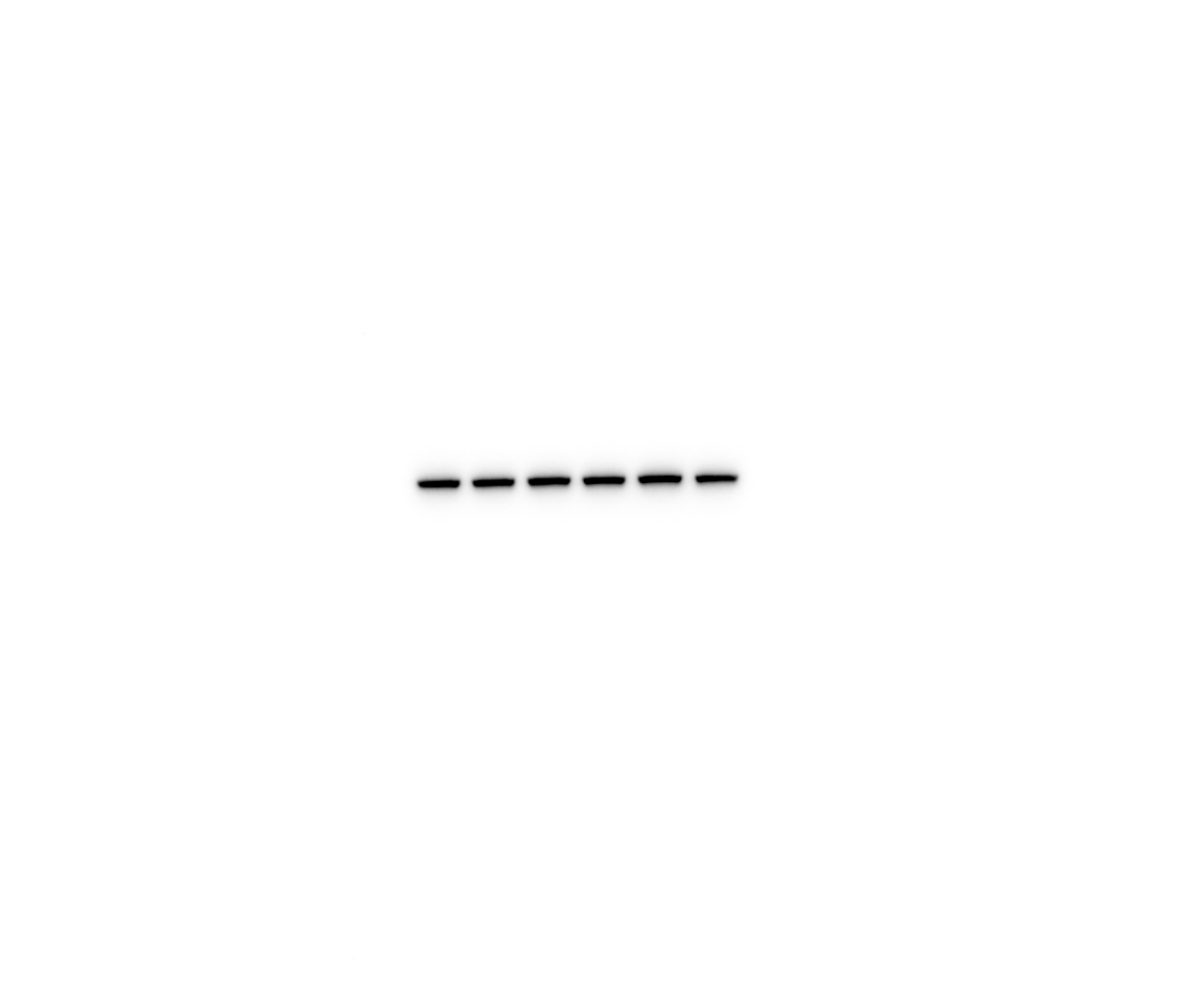

Supplement: Supplementary file 7 [file Data_Sheet_1.ZIP › PI3K-AKT-mTOR/a┬-actin/1.Tif]

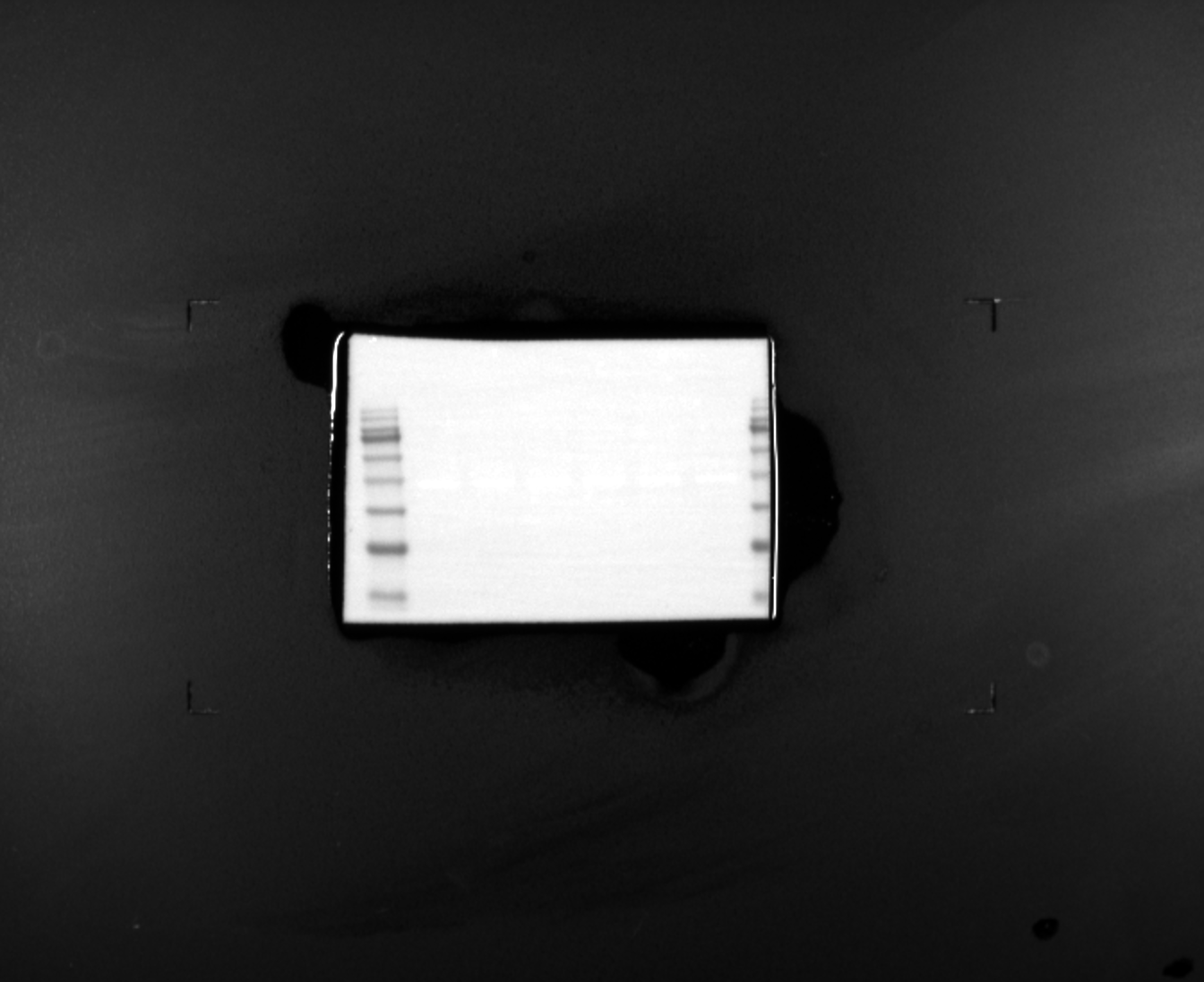

Supplement: Supplementary file 7 [file Data_Sheet_1.ZIP › PI3K-AKT-mTOR/a┬-actin/1-t.Tif]

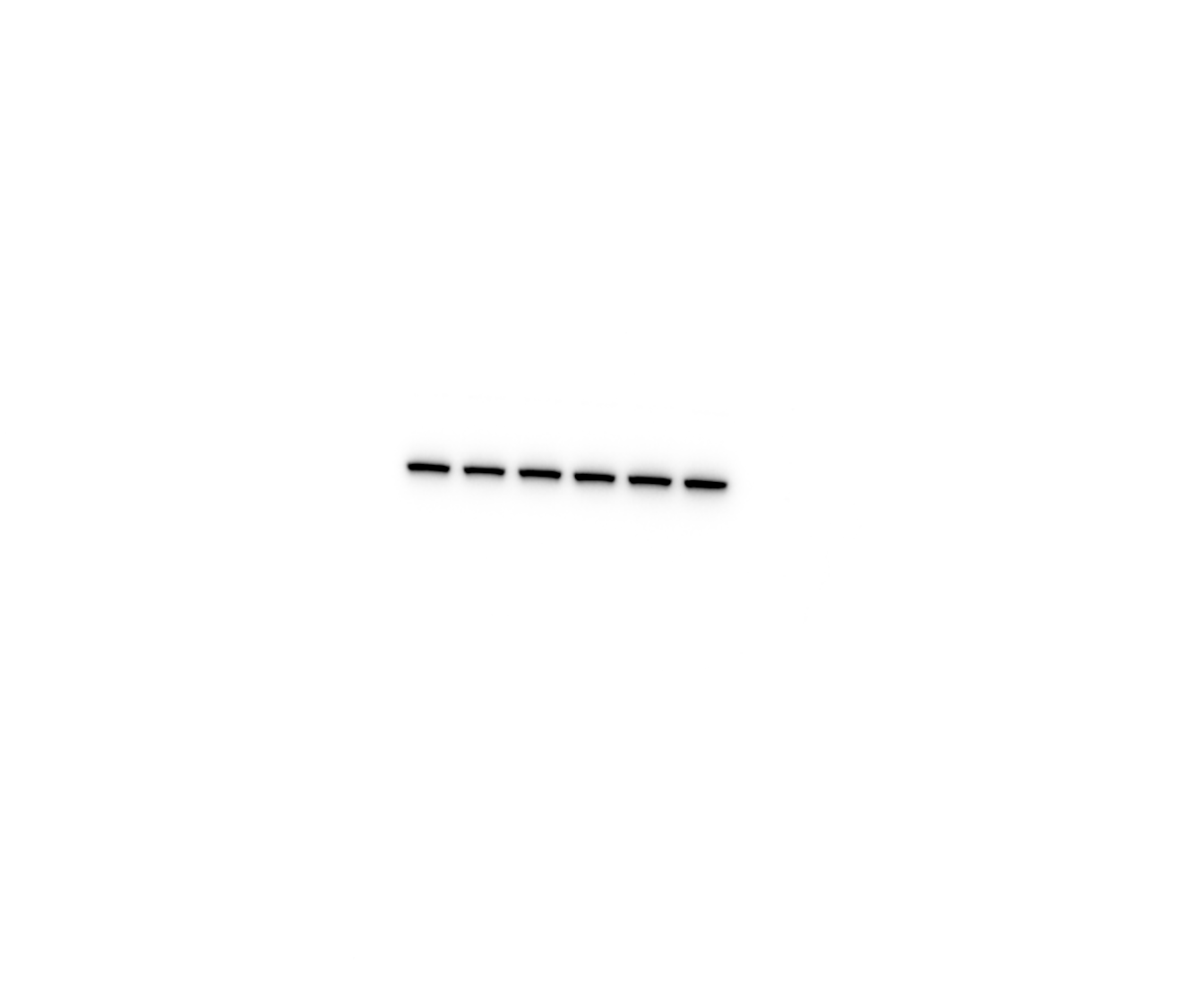

Supplement: Supplementary file 7 [file Data_Sheet_1.ZIP › PI3K-AKT-mTOR/a┬-actin/2.Tif]

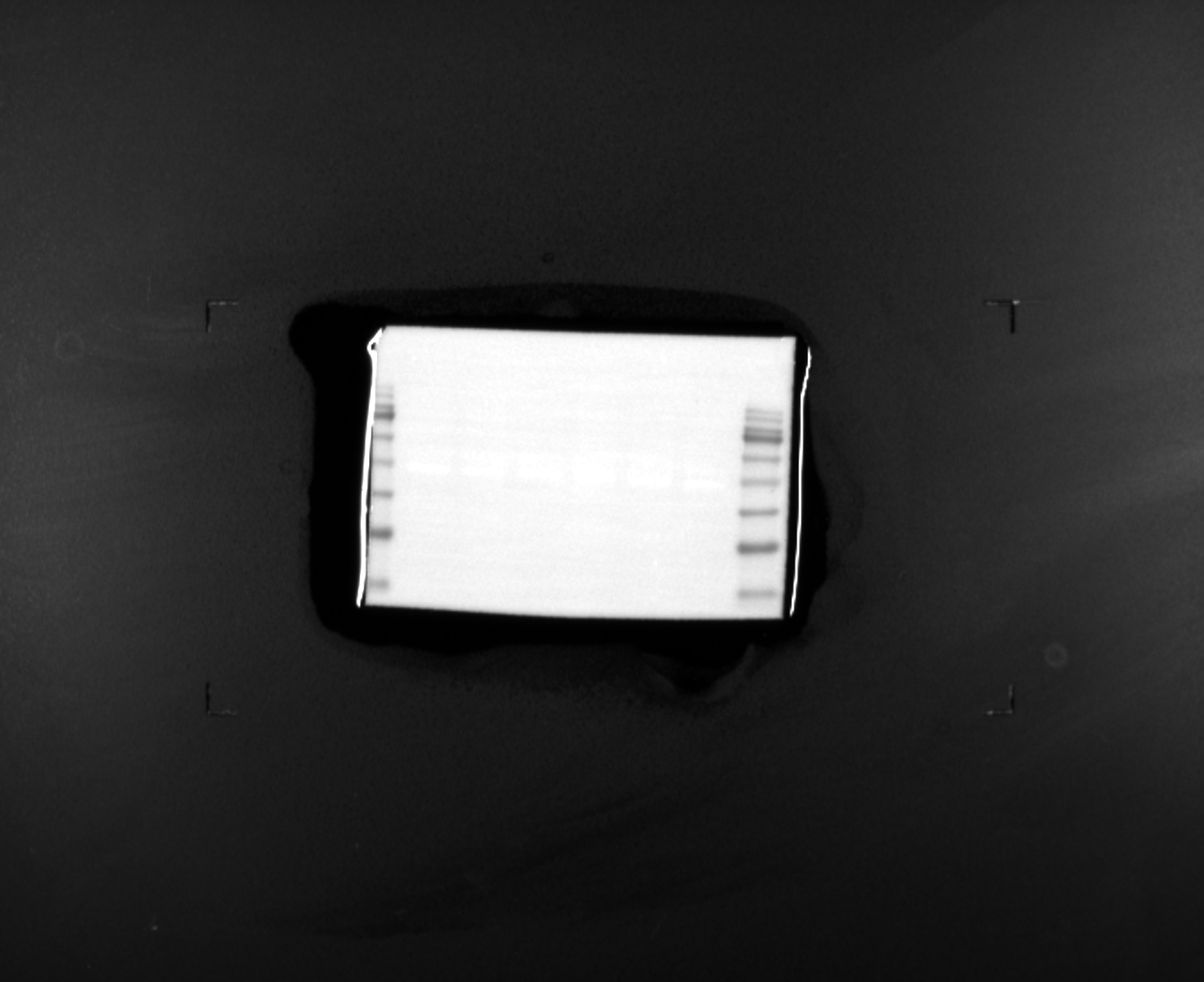

Supplement: Supplementary file 7 [file Data_Sheet_1.ZIP › PI3K-AKT-mTOR/a┬-actin/2-t.Tif]

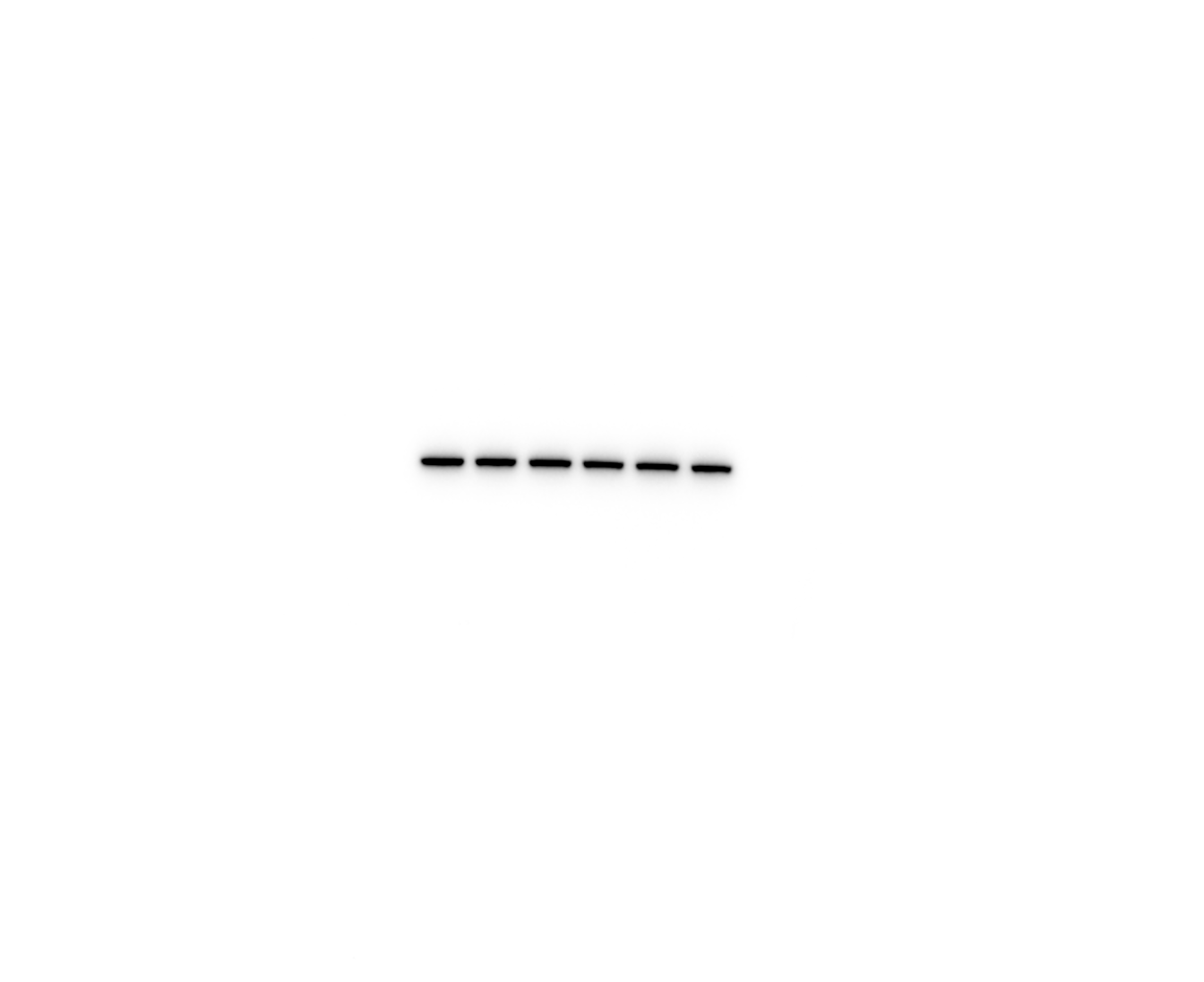

Supplement: Supplementary file 7 [file Data_Sheet_1.ZIP › PI3K-AKT-mTOR/a┬-actin/3.Tif]

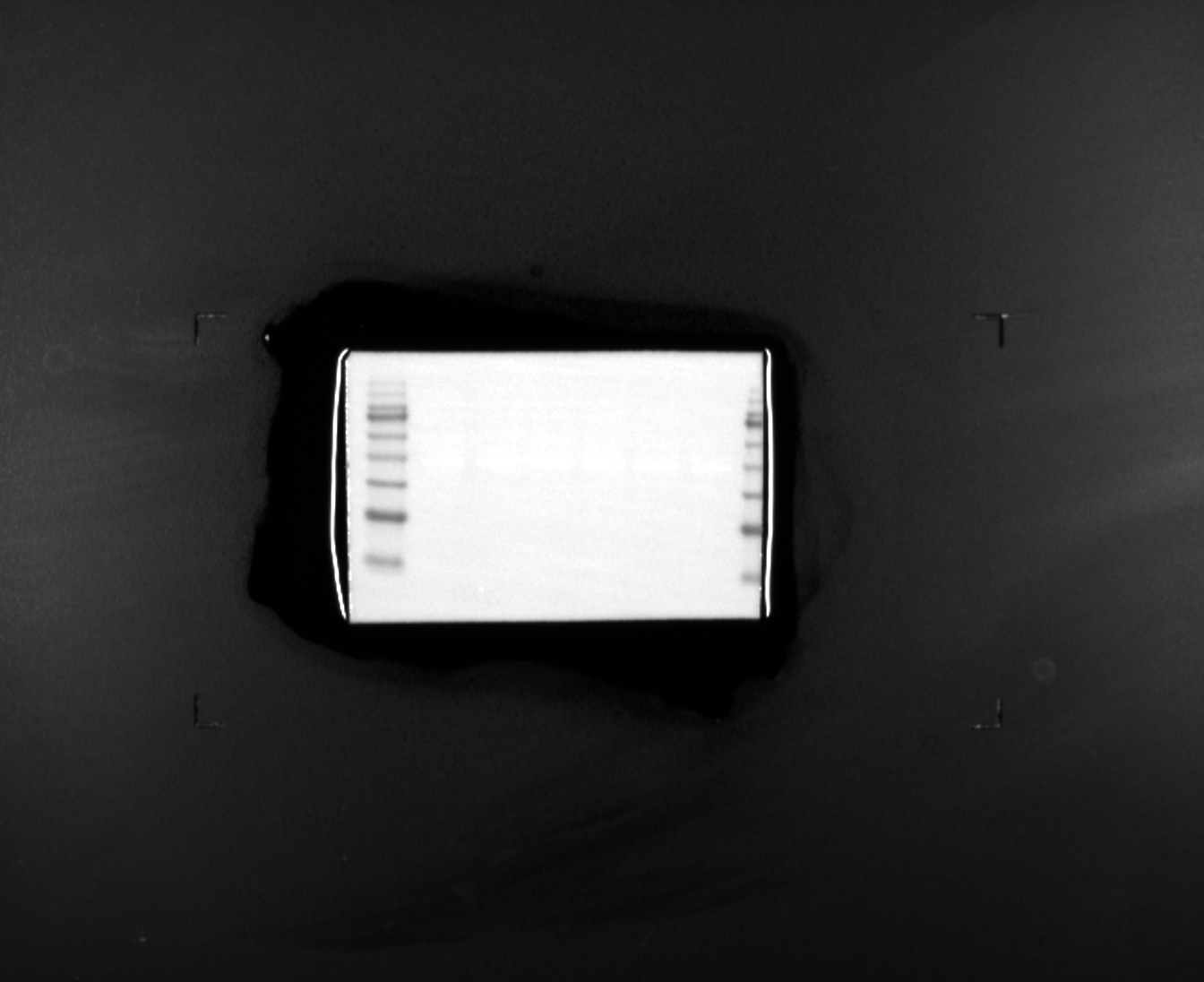

Supplement: Supplementary file 7 [file Data_Sheet_1.ZIP › PI3K-AKT-mTOR/a┬-actin/3-t.Tif]
